# Chelating Rotaxane Ligands as Fluorescent Sensors for Metal Ions

**DOI:** 10.1002/anie.201712931

**Published:** 2018-04-06

**Authors:** Mathieu Denis, Jessica Pancholi, Kajally Jobe, Michael Watkinson, Stephen M. Goldup

**Affiliations:** ^1^ Chemistry University of Southampton Highfield Southampton SO17 1BJ UK; ^2^ School of Biological and Chemical Sciences Queen Mary University of London Mile End Road London E1 4NS UK

**Keywords:** fluorescent probes, rotaxanes, sensors, supramolecular chemistry, zinc

## Abstract

Although metal‐ion‐binding interlocked molecules have been under intense investigation for over three decades, their application as scaffolds for the development of sensors for metal ions remains underexplored. In this work, we demonstrate the potential of simple rotaxanes as metal‐ion‐responsive ligand scaffolds through the development of a proof‐of‐concept selective sensor for Zn^2+^.

Small‐molecule fluorescent probes are powerful tools for visualizing metal ions in living systems due to their rapid response times and potential for non‐invasive, high resolution, and quantitative imaging.^1^ In particular, the development of small‐molecule sensors^2^, ^3^ for the detection and quantification of Zn^2+^ in vivo has attracted considerable recent attention due to the spectroscopically silent nature of the d^10^ Zn^2+^ ion, combined with the recognition that changes in zinc homeostasis are associated with high‐morbidity diseases such as Alzheimer's disease,^4^ Type II diabetes,^5^ and age‐related macular degeneration.^6^

Such small‐molecule probes are generally composed of a multidentate chelating ligand linked to a fluorophore whose output is modulated by the metal binding event. Mechanically interlocked molecules,^7^ particularly those synthesized using metal‐mediated approaches,^8^ often possess a well‐defined binding pocket containing multiple donor atoms for metal ions.^9^ Such multidentate “mechanically chelating” ligands^10^ seem ideal for the development of metal‐selective ligands and related metal‐ion sensors by exploiting the size and shape of the three dimensional cavity formed by the mechanical bond. However, almost all interlocked molecules that display a fluorescent response^11^ upon metal binding rely on large‐amplitude motion in relatively structurally complex molecular shuttles.^12^, ^13^, ^14^ Furthermore, in most cases, selectivity between competing analytes is not reported. Indeed, to our knowledge, only one example has been reported in which the mechanical bond is used to generate a metal binding pocket to report the binding of competing analytes; in 2004 Hiratani and co‐workers disclosed a [1]rotaxane that selectively binds Li^+^ over Na^+^ and K^+^ and reports metal binding through a “switch on” fluorescence response.^15^, ^16^

Given that the synthesis of mechanically chelating ligands is now relatively simple, it is perhaps surprising that these scaffolds have been overlooked in the development of cation sensors, particularly since a related strategy for the sensing of anions has been developed by Beer and co‐workers.^17^ We thus set out to demonstrate the potential of the mechanical bond as a structural motif in the development of selective metal‐ion sensors through the demonstration of a proof‐of‐concept selective sensor for Zn^2+^. Herein, we report not only that is this approach successful, but that relatively small structural changes in the axle component lead to large changes in the photophysical response to divalent metal ions.

We synthesized rotaxane **4** 18 in excellent yield (88 %) using the active‐template^19^ Cu‐mediated alkyne–azide cycloaddition (AT‐CuAAC) reaction^20^, ^21^ between azido fluorophore **3** and acetylene **2** in the presence of readily available bipyridine macrocycle **1** 22 and [Cu(MeCN)_4_]PF_6_. Addition of one equivalent of Zn(ClO_4_)_2_⋅6 H_2_O to a solution of **4** in CD_3_CN resulted in large changes in the ^1^H NMR spectrum (see Figure S39 in the Supporting Information), which is consistent with the binding of the metal ion into the cavity of the macrocycle, confirming that **4** is capable of acting as a ligand. The behavior of rotaxane **4** as a metal‐responsive sensor for Zn^2+^ was investigated by fluorescence titration with Zn(ClO_4_)_2_⋅6 H_2_O. Portion‐wise addition of Zn^2+^ to rotaxane **4** in MeCN led to monotonic quenching of the emission at 560 nm^23^ that plateaued once a full equivalent had been added (Figure 1 a). In contrast, titration of the non‐interlocked axle with Zn^2+^ revealed no change by UV/Vis or fluorescence spectroscopy (Figure S61).^24^

**Figure 1:**
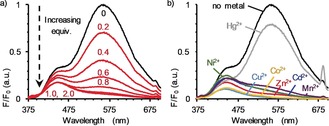
Emission profile of rotaxane **4** (MeCN, 100 μm, *λ*_ex_=343 nm) in the presence of varying amounts of Zn(ClO_4_)_2_⋅6 H_2_O (a), and in the presence of 5 equiv M(ClO_4_)_2_ (b). No emission was observed with Fe(ClO_4_)_2_.

Rotaxane **4** is a candidate “switch off” fluorescent sensor for Zn^2+^. However, examination of the selectivity of this response revealed a complete lack of discrimination between Zn^2+^ and selected divalent metal cations; addition of M(ClO_4_)_2_ (M=Mn^2+^, Fe^2+^, Co^2+^, Ni^2+^, Cu^2+^, Cd^2+^, Hg^2+^) to a solution of **4** led to quenching of the emission to a greater or lesser extent than that observed with Zn^2+^ (Figure 1 b).^23^ Thus, **4** cannot be classed as a metal‐ion sensor since, although it responds to metal‐ion binding, it cannot discriminate between competing analytes.

Having confirmed that the binding of metal ions within the cavity of the rotaxane can, in principle, lead to an optical response, we extended our investigation to heteroatom‐substituted naphthalimide rotaxanes **5**–**7** (Figure 2), which were readily synthesized in good to excellent yield (62 %, 72 % and 86 % respectively, see the Supporting Information). These were selected because heteroatoms can potentially interact directly with the metal ion and are known to significantly alter the photophysical properties of naphthalimide fluorophores.^25^ ^1^H NMR spectroscopy (Figures S39–41) confirmed that rotaxanes **5**–**7** act as ligands for Zn^2+^, and UV/Vis titration revealed excellent goodness of fit to a 1:1 binding isotherm; addition of Zn(ClO_4_)_2_⋅6 H_2_O resulted in the appearance of absorbance bands at 312 and 322 nm corresponding to the metal‐bound bipyridine moiety (Figures S46–48).^26^

**Figure 2:**
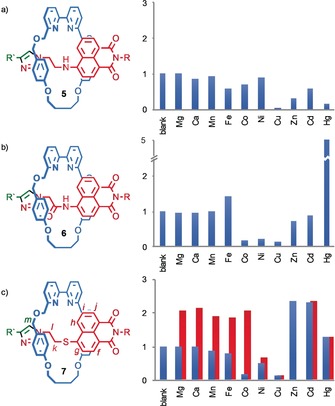
Rotaxanes a) **5** (*λ*_ex_=435 nm), b) **6** (*λ*_ex_=380 nm), and c) **7** (*λ*_ex_=379 nm) and their fluorescence response to 5 equiv M(ClO_4_)_2_ (MeCN, 100 μm; blue bars). Red bars in (c) refer to the fluorescence response upon sequential addition of M^2+^ followed by Zn^2+^ (5 equiv each). R=CH_2_C(H)Ph_2_, R′=3,5‐di‐^*t*^Bu‐C_6_H_3_.

The fluorescence response of rotaxanes **5**–**7** to the binding of selected divalent metal ions revealed very different behavior. Similar to rotaxane **4**, rotaxane **5** displays a simple switch‐off response to Zn^2+^ and the majority of other metal ions investigated (Figure 2 a), although the extent of quenching varied considerably depending on the metal ion. In contrast, rotaxane **6** (Figure 2 b) displays little or no response to Mg^2+^, Ca^2+^, or Mn^2+^, a switch‐off response of varying degree to Co^2+^, Ni^2+^, Cu^2+^ Zn^2+^, and Cd^2+^ and a weak switch‐on response to Fe^2+^. Strikingly, Hg^2+^ produced a significant switch‐on response, suggesting that rotaxane **6** is a good starting point for the development of a selective sensor for Hg^2+^. Pleasingly, addition of Zn^2+^ to rotaxane **7** triggers a switch‐on response with a concomitant blue shift in the emission of 15 nm. All other metal ions, with the exception of Cd^2+^, which produces a switch‐on response and a blue shift of 8 nm, produce a switch‐off or no response (Figure 2 c). Competition experiments demonstrate that in many cases, Zn^2+^ is also able to displace metal ions from ligand **7**; addition of M^2+^ followed by Zn^2+^ to **7** resulted in recovery of fluorescence in the case of Mg^2+^, Ca^2+^, Mn^2+^, Fe^2+^, and Co^2+^.

Having identified rotaxane **7** as a switch‐on sensor for Zn^2+^, albeit with Cd^2+^ as a confounding analyte,^27^ we investigated its behavior in more detail. The ^1^H NMR spectrum of **7** (Figure 3 b and Scheme 1) displays a number of differences to the corresponding non‐interlocked axle (Figure 3 a). In particular triazole proton H_*m*_ appears significantly deshielded in the interlocked structure by Δ*δ*=1.36 ppm, which is consistent with an expected C−H⋅⋅⋅N hydrogen bond with the bipyridine moiety,^28^ and alkyl protons H_*k*_ and H_*l*_ appear at lower ppm, suggesting that they engage in C−H⋅⋅⋅π contacts with the aromatic rings of the macrocycle. Crystals of **7** suitable for X‐ray analysis were grown by slow evaporation from MeCN. The solid‐state structure is largely consistent with the interactions proposed to be present in solution (Figure 4 a); triazole proton H_*m*_ is engaged in a C−H⋅⋅⋅N hydrogen bond with the bipyridine nitrogens, as is one of H_*k*_, and one each of protons H_*k*_ and H_*l*_ are in close contact with the phenyl rings of the macrocycle.

**Figure 3:**
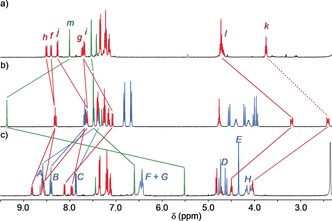
Partial ^1^H NMR spectra (CD_3_CN, 400 MHz, 298 K) of a) the non‐interlocked axle of rotaxane **7**, b) rotaxane **7**, and c) rotaxane **7**+Zn(ClO_4_)_2_⋅6 H_2_O. For labelling scheme, see Scheme 1 (macrocycle) and Figure 2 (axle).

**Figure 4:**
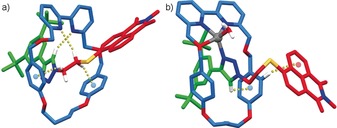
a) Solid‐state structure of rotaxane **7** (selected distances in Å: C−H_*m*_⋅⋅⋅N=2.46, C‐H_*k*_⋅⋅⋅N=2.71, C−H_*k*_⋅⋅⋅π=2.66, C‐H_*l*_⋅⋅⋅π=2.79; dihedral angle C_*k*_‐S‐C‐C_*ipso*_=8.2°). b) Solid‐state structure of [Zn(**7**)]^2+^ (selected distances: C−O⋅⋅⋅Zn=2.28, C−H_*m*_⋅⋅⋅π=2.74, C−H_*F*_⋅⋅⋅π=2.86; dihedral angle C_*k*_‐S‐C‐C_*ipso*_=29.9°).

**Scheme 1:**
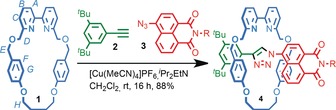
Synthesis of fluorescent rotaxane **4** using the AT‐CuAAC reaction. R=CH_2_C(H)Ph_2_.

Portion‐wise addition of Zn(ClO_4_)_2_⋅6 H_2_O to **7** in CD_3_CN led to broadening of the ^1^H NMR resonances corresponding to **7** and the appearance of a new set of resonances assigned to [Zn(**7**)]^2+^ (Figure S42). Once one equivalent of Zn^2+^ had been added (Figure 3 c), no further change was observed. Strikingly, in addition to the expected deshielding of the bipyridine resonances H_*A*_, H_*B*_, and H_*C*_, the triazole and alkyl resonances shift significantly upon Zn^2+^ binding. H_*m*_ is observed at 5.52 ppm in the metal complex (Δ*δ*=−3.84 ppm), suggesting it is considerably shielded relative to the non‐interlocked axle, while H_*k*_ and H_*l*_ shift to higher ppm, suggesting that the shielding C−H⋅⋅⋅π interactions are interrupted.

The solid‐state structure of [Zn(**7**)](OTf)_2_ obtained by slow evaporation of a MeCN solution confirms coordination of Zn^2+^ within the macrocycle cavity and exhibits a number of features consistent with the solution‐state ^1^H NMR data. The Zn^2+^ ion is coordinated by the bipyridine and triazole N‐donors, alongside one of the aliphatic ether O‐donors and a water molecule.^29^ This coordination interrupts the triazole C−H⋅⋅⋅N hydrogen bond and this proton is now engaged in a shielding C−H⋅⋅⋅π interaction with one of the macrocycle phenyl rings. The shielding C−H⋅⋅⋅π interactions of H_*k*_ and H_*l*_ are also interrupted. Interestingly, in the solid state, coordination leads to the appearance of a C−H⋅⋅⋅π interaction between one of the aromatic phenyl ring protons and the naphthalene rings and a large change in the dihedral angle about the S‐naphthyl bond. With the obvious caveat that the solid‐state structure of [Zn(**7**)]^2+^ is not necessarily representative of the solution‐state (co)conformation, the observed changes in the interactions between the macrocycle and fluorophore component, along with the changes in the conjugation between the S‐donor and the naphthalene core and the altered dihedral angle, suggest that the changes in emission properties of **7** on metal binding may be due to changes in the (co)conformation of the ensemble.

The binding of rotaxane **7** with Zn^2+^ was determined to be extremely strong (*K*_d_<10^−8^ m ^−1^) by UV/Vis titration^30^ with non‐linear regression analysis (Figure 5 a), stronger than that of macrocycle **1** alone (*K*_d_=8.9×10^−8^ m ^−1^; Figure S57), suggesting that the mechanically chelating triazole ligand significantly enhances Zn^2+^ binding in **7**.^31^ Conversely, the binding of rotaxane **7** to Cd^2+^ (*K*_d_=3.6×10^−5^ m ^−1^; Figure 5 b) is about three orders of magnitude weaker than that of macrocycle **1** alone (7.5×10^−8^ m ^−1^; Figure S59). The difference in binding strength for **7** with Zn^2+^ and Cd^2+^ was further corroborated by ^1^H NMR titration. In the presence of 1 equiv of both Zn^2+^ and Cd^2+^, [Zn(**7**)]^2+^ is observed to form selectively (Figure S43), whereas the same experiment with macrocycle **1** produced a 4:1 mixture of Zn^2+^ and Cd^2+^ complexes (Figure S45). These results demonstrate that the mechanical bond imparts a significant degree of selectivity to the binding of otherwise similar metal ions, perhaps due to the different sizes of the Zn^2+^ and Cd^2+^ ions (88 pm vs. 109 pm, respectively)^32^ or by sterically excluding additional ligands from the coordination sphere of the metal ion.

**Figure 5:**
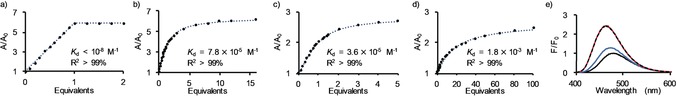
UV/Vis (*λ*=322 nm) titrations of **7** (100 μm) with M(ClO_4_)_2_ as a function of a) Zn^2+^, b) Zn^2+^ (2 % H_2_O/MeCN), c) Cd^2+^ (MeCN), and d) Cd^2+^ (2 % H_2_O‐MeCN). e) Emission spectra (*λ*_ex_=380 nm) of **7** (black), **7**+5 equiv Cd^2+^ (blue), **7**+5 equiv Zn^2+^ (red), **7**+5 equiv each Zn^2+^ and Cd^2+^ (black dashed) in 2 % H_2_O/MeCN.

The stronger binding of Zn^2+^ compared with Cd^2+^ allowed us to use a more competitive solvent mixture (2 % water in MeCN) to impart selectivity to sensor **7**. In the presence of H_2_O (Figures 5 c,d), the binding of both Zn^2+^ and Cd^2+^ to **7** was diminished (*K*_d_=7.8×10^−5^ and 1.8×10^−3^ m ^−1^ respectively). As a result, whereas 1 equiv of Zn^2+^ achieved 50 % of *F*_max_ and saturation was achieved at approximately 12 equiv, Cd^2+^ required around 20 equiv to achieve 50 % switch on and around 100 equiv to achieve saturation, demonstrating that under these conditions, Cd^2+^ is not bound by **7** to a significant extent. Thus, whereas addition of 5 equivalents of Cd^2+^ to **7** in MeCN/H_2_O leads to a weak response, when Zn^2+^ is added to the same solution, the expected switching on of luminescence is observed, thus demonstrating selectivity for Zn^2+^ over Cd^2+^ (Figure 5 e).

In conclusion, we have demonstrated that relatively simple interlocked molecules can provide an excellent scaffold for the design of metal ion sensors. Importantly we show that the binding pocket provided by the mechanical bond can impart not only an optical response but also a degree of binding selectivity, as in the case of rotaxane **7**. It is also noteworthy that, in addition to sensor **7**, which shows the desired Zn^2+^‐selective response in MeCN/H_2_O, rotaxane **6** also appears to show a selective switch‐on response, in this case to Hg^2+^.^33^ The mode of switching, at least in the case of **7**, appears to be reorientation of the components upon metal binding, thereby altering the relative positions of the fluorophore and the macrocycle and leading to an enhancement of fluorescence, but this requires more detailed investigation. The origin of the different behaviors of **4**–**6** also requires further investigation; since it appears that the different linker units are not directly involved in the binding event, it seems likely that the specific photophysical properties of the fluorophore unit are important.^25^ From a practical viewpoint, the next step in the development of interlocked hosts for the detection of metal ions in biological systems is to render them water soluble, a task that is ongoing in our laboratories and is greatly facilitated by the synthetic flexibility of the AT‐CuAAC reaction. More generally, the results presented here suggest that, although interlocked molecular machines remain an exciting and important direction for the field, the use of the mechanical bond as a structural feature, for instance in the design of mechanically chelating ligands, has the potential to lead to new developments in a range of areas.

## Conflict of interest

The authors declare no conflict of interest.

## Supporting information

As a service to our authors and readers, this journal provides supporting information supplied by the authors. Such materials are peer reviewed and may be re‐organized for online delivery, but are not copy‐edited or typeset. Technical support issues arising from supporting information (other than missing files) should be addressed to the authors.

Supplementary

## References

[1] Reviews:

[1a] D. W. Domaille , E. L. Que , C. J. Chang , Nat. Chem. Biol. 2008, 4, 168; 18277978 10.1038/nchembio.69

[1b] J. Chan , S. C. Dodani , C. J. Chang , Nat. Chem. 2012, 4, 973; 23174976 10.1038/nchem.1500 PMC4096518

[1c] K. P. Carter , A. M. Young , A. E. Palmer , Chem. Rev. 2014, 114, 4564; 24588137 10.1021/cr400546e PMC4096685

[1d] Y. L. Pak , K. M. K. Swamy , J. Yoon , Sensors 2015, 15, 24374; 26402684 10.3390/s150924374 PMC4610470

[1e] X. H. Qian , Z. C. Xu , Chem. Soc. Rev. 2015, 44, 4487; 25556818 10.1039/c4cs00292j

[1f] J. Li , D. Yim , W.-D. Jang , J. Yoon , Chem. Soc. Rev. 2017, 46, 2437. 27711665 10.1039/c6cs00619a

[2] Review of Zn^2+^ sensing in biology: W. Maret , Metallomics 2015, 7, 202. 25362967 10.1039/c4mt00230j

[3] Review of genetically encoded sensors: S. J. A. Aper , P. Dieriekx , M. Merkx , ACS Chem. Biol. 2016, 11, 2854. 27547982 10.1021/acschembio.6b00453 PMC5080634

[4] K. J. Barnham , A. I. Bush , Curr. Opin. Chem. Biol. 2008, 12, 222. 18342639 10.1016/j.cbpa.2008.02.019

[5] G. A. Rutter , Islets 2010, 2, 49. 21099294 10.4161/isl.2.1.10259

[6] I. Lengyel , J. M. Flinn , T. Peto , D. H. Linkous , K. Cano , A. C. Bird , A. Lanzirotti , C. J. Frederickson , F. J. G. M. van Kuijk , Exp. Eye Res. 2007, 84, 772. 17313944 10.1016/j.exer.2006.12.015

[7a] E. A. Neal , S. M. Goldup , Chem. Commun. 2014, 50, 5128; 10.1039/c3cc47842d 24434901

[7b] M. Xue , Y. Yang , X. Chi , X. Yan , F. Huang , Chem. Rev. 2015, 115, 7398; 25734835 10.1021/cr5005869

[7c] C. J. Bruns , J. F. Stoddart , The Nature of the Mechanical Bond: From Molecules to Machines, Wiley, Hoboken, 2016.

[8a] J. E. Beves , B. A. Blight , C. J. Campbell , D. A. Leigh , R. T. McBurney , Angew. Chem. Int. Ed. 2011, 50, 9260; 10.1002/anie.201007963 21928462

[8b] J. E. M. Lewis , P. D. Beer , S. J. Loeb , S. M. Goldup , Chem. Soc. Rev. 2017, 46, 2577. 28447678 10.1039/c7cs00199a

[9a] A. M. Albrecht-Gary , Z. Saad , C. O. Dietrich-Buchecker , J.-P. Sauvage , J. Am. Chem. Soc. 1985, 107, 3205;

[9b] A. J. Blake , C. O. Dietrich-Buchecker , T. I. Hyde , J.-P. Sauvage , M. Schröder , Chem. Commun. 1989, 1663;

[9c] C. O. Dietrich-Buchecker , J.-M. Kern , J.-P. Sauvage , Chem. Commun. 1985, 760;

[9d] C. Dietrich-Buchecker , J.-P. Sauvage , J. M. Kern , J. Am. Chem. Soc. 1989, 111, 7791;

[9e] A. Masood , P. S. Zacharias , Polyhedron 1991, 10, 811;

[9f] D. A. Leigh , P. J. Lusby , A. M. Z. Slawin , D. B. Walker , Angew. Chem. Int. Ed. 2005, 44, 4557; 10.1002/anie.200500004 15973751

[9g] G. Baggi , S. J. Loeb , Angew. Chem. Int. Ed. 2016, 55, 12533; 10.1002/anie.201607281 27592565

[9h] G. Baggi , S. J. Loeb , Chem. Eur. J. 2017, 23, 14163; 28851068 10.1002/chem.201703485

[9i] T. H. Ngo , J. Labuta , G. N. Lim , W. A. Webre , F. D'Souza , P. A. Karr , J. E. M. Lewis , J. P. Hill , K. Ariga , S. M. Goldup , Chem. Sci. 2017, 8, 6679. 10.1039/c7sc03165c PMC6103255 30155230

[10] “Mechanically chelating” is used to describe ligands in which donor atoms bridge the mechanical bond: J. E. M. Lewis , M. Galli , S. M. Goldup , Chem. Commun. 2017, 53, 298.

[11a] X. Ma , H. Tian , Chem. Soc. Rev. 2010, 39, 70; 20023838 10.1039/b901710k

[11b] M. J. Langton , P. D. Beer , Acc. Chem. Res. 2014, 47, 1935. 24708030 10.1021/ar500012a

[12a] W. Zhou , J. Li , X. He , C. Li , J. Lv , Y. Li , S. Wang , H. Liu , D. Zhu , Chem. Eur. J. 2008, 14, 754; 17960552 10.1002/chem.200701105

[12b] S. Y. Hsueh , C. C. Lai , S. H. Chiu , Chem. Eur. J. 2010, 16, 2997. 20157908 10.1002/chem.200903304

[13] Examples where the response is not due to shuttling:

[13a] N. Armaroli , L. De Cola , V. Balzani , J.-P. Sauvage , C. O. Dietrich-Buchecker , J.-M. Kern , A. Bailalb , J. Chem. Soc. Dalton Trans. 1993, 3241;

[13b] N. Armaroli , L. De Cola , V. Balzani , F. Barigelletti , L. Flamigni , J. P. Sauvage , C. Hemmert , J. Am. Chem. Soc. 1994, 116, 5211;

[13c] M. J. MacLachlan , A. Rose , T. M. Swager , J. Am. Chem. Soc. 2001, 123, 9180; 11552834 10.1021/ja016228j

[13d] P. H. Kwan , M. J. MacLachlan , T. M. Swager , J. Am. Chem. Soc. 2004, 126, 8638; 15250701 10.1021/ja048506v

[13e] P. H. Kwan , T. M. Swager , J. Am. Chem. Soc. 2005, 127, 5902; 15839689 10.1021/ja042535o

[13f] Y. Nakatani , Y. Furusho , E. Yashima , Angew. Chem. Int. Ed. 2010, 49, 5463; 10.1002/anie.201002382 20583022

[14] S. Erbas-Cakmak , D. A. Leigh , C. T. McTernan , A. L. Nussbaumer , Chem. Rev. 2015, 115, 10081. 26346838 10.1021/acs.chemrev.5b00146 PMC4585175

[15] K. Hiratani , M. Kaneyama , Y. Nagawa , E. Koyama , M. Kanesato , J. Am. Chem. Soc. 2004, 126, 13568. 15493885 10.1021/ja046929r

[16] An example of selective discrimination of alkali metal ions by a rotaxane host using ^1^H NMR: N.-C. Chen , P.-Y. Huang , C.-C. Lai , Y.-H. Liu , Y. Wang , S.-M. Peng , S.-H. Chiu , Chem. Commun. 2007, 4122. 10.1039/b706461f 17925949

[17] See Ref. [11b] and:

[17a] D. Curiel , P. D. Beer , Chem. Commun. 2005, 1909; 10.1039/b418878k 15795784

[17b] S. R. Bayly , T. M. Gray , M. J. Chmielewski , J. J. Davis , P. D. Beer , Chem. Commun. 2007, 2234; 10.1039/b701796k 17534501

[17c] M. J. Chmielewski , J. J. Davis , P. D. Beer , Org. Biomol. Chem. 2009, 7, 415; 19156302 10.1039/b818351a

[17d] N. H. Evans , P. D. Beer , Org. Biomol. Chem. 2011, 9, 92; 21063586 10.1039/c0ob00458h

[17e] N. H. Evans , C. J. Serpell , N. G. White , P. D. Beer , Chem. Eur. J. 2011, 17, 12347; 21953676 10.1002/chem.201101811

[17f] N. H. Evans , C. J. Serpell , P. D. Beer , Chem. Commun. 2011, 47, 8775; 10.1039/c1cc13247d 21743929

[17g] L. M. Hancock , E. Marchi , P. Ceroni , P. D. Beer , Chem. Eur. J. 2012, 18, 11277; 22847976 10.1002/chem.201201422

[17h] J. Lehr , T. Lang , O. A. Blackburn , T. A. Barendt , S. Faulkner , J. J. Davis , P. D. Beer , Chem. Eur. J. 2013, 19, 15898; ; 24127251 10.1002/chem.201302886 PMC4517173

[17i] M. J. Langton , S. W. Robinson , I. Marques , V. Félix , P. D. Beer , Nat. Chem. 2014, 6, 1039; 25411880 10.1038/nchem.2111

[17j] M. J. Langton , I. Marques , S. W. Robinson , V. Félix , P. D. Beer , Chem. Eur. J. 2016, 22, 185. 26626866 10.1002/chem.201504018 PMC4832824

[18] Rotaxane **4** is a derivative of previously reported fluorescent rotaxane **S15** (R=Et, Ref. [22a]). The metal binding behaviors of **4** and **S15** are identical (see the Supporting Information). However, whereas **S15** is stable, analogues of rotaxanes **5**–**7** with R=Et de-threaded on standing at RT in MeCN. Presumably due to the increased axle flexibility of **5**–**7**.

[19a] J. D. Crowley , S. M. Goldup , A.-L. Lee , D. A. Leigh , R. T. McBurney , Chem. Soc. Rev. 2009, 38, 1530; 19587949 10.1039/b804243h

[19b] S. Saito , J. Inclusion Phenom. Macrocyclic Chem. 2015, 82, 437;

[19c] M. Denis , S. M. Goldup , Nat. Rev. Chem. 2017, 1, 0061.

[20a] V. Aucagne , K. D. Hänni , D. A. Leigh , P. J. Lusby , D. B. Walker , J. Am. Chem. Soc. 2006, 128, 2186; 16478152 10.1021/ja056903f

[20b] V. Aucagne , J. Berna , J. D. Crowley , S. M. Goldup , K. D. Hänni , D. A. Leigh , P. J. Lusby , V. E. Ronaldson , A. M. Z. Slawin , A. Viterisi , D. B. Walker , J. Am. Chem. Soc. 2007, 129, 11950. 17845039 10.1021/ja073513f

[21] Selected applications of the AT-CuAAC reaction:

[21a] S. M. Goldup , D. A. Leigh , T. Long , P. R. McGonigal , M. D. Symes , J. Wu , J. Am. Chem. Soc. 2009, 131, 15924; 19807083 10.1021/ja9070317

[21b] S. M. Goldup , D. A. Leigh , P. R. McGonigal , V. E. Ronaldson , A. M. Z. Slawin , J. Am. Chem. Soc. 2010, 132, 315; 19968281 10.1021/ja9080716

[21c] P. E. Barran , H. L. Cole , S. M. Goldup , D. A. Leigh , P. R. McGonigal , M. D. Symes , J. Wu , M. Zengerle , Angew. Chem. Int. Ed. 2011, 50, 12280; 10.1002/anie.201105012 21919173

[21d] A. Fernandes , A. Viterisi , V. Aucagne , D. A. Leigh , S. Papot , Chem. Commun. 2012, 48, 2083; 10.1039/c2cc17458h 22227715

[21e] A. Noor , S. C. Moratti , J. D. Crowley , Chem. Sci. 2014, 5, 4283;

[21f] A. Noor , W. K. C. Lo , S. C. Moratti , J. D. Crowley , Chem. Commun. 2014, 50, 7044; 10.1039/c4cc03077j 24850165

[21g] M. Denis , L. Qin , P. Turner , K. A. Jolliffe , S. M. Goldup , Angew. Chem. Int. Ed. 2018, https://doi.org/10.1002/anie.201713105; 10.1002/anie.201713105 PMC5947583 29393993

[22a] H. Lahlali , K. Jobe , M. Watkinson , S. M. Goldup , Angew. Chem. Int. Ed. 2011, 50, 4151; 10.1002/anie.201100415 21462287

[22b] E. A. Neal , S. M. Goldup , Chem. Sci. 2015, 6, 2398; 29308153 10.1039/c4sc03999h PMC5645920

[22c] J. E. M. Lewis , R. J. Bordoli , M. Denis , C. J. Fletcher , M. Galli , E. A. Neal , E. M. Rochette , S. M. Goldup , Chem. Sci. 2016, 7, 3154; 10.1039/c6sc00011h PMC6005271 29997807

[22d] E. A. Neal , S. M. Goldup , Angew. Chem. Int. Ed. 2016, 55, 12488; 10.1002/anie.201606640 PMC5113769 27600208

[22e] J. E. M. Lewis , J. Winn , L. Cera , S. M. Goldup , J. Am. Chem. Soc. 2016, 138, 16329; 27700073 10.1021/jacs.6b08958

[22f] J. E. M. Lewis , J. Winn , S. M. Goldup , Molecules 2017, 22, 89. 10.3390/molecules22010089 PMC6155830 28075366

[23] The second emission of **4** is tentatively assigned to intramolecular exciplex formation. A similar effect was observed previously, including metal-induced quenching of the exciplex emission (Refs [13c–f]). This unexpected effect is the subject of ongoing investigations.

[24] 1,2,3-Triazole ligands are known as ligands for metal ions: H. Struthers , T. L. Mindt , R. Schibli , Dalton Trans. 2010, 39, 675; 20066208 10.1039/b912608b

[25] S. Banerjee , E. B. Veale , C. M. Phelan , S. A. Murphy , G. M. Tocci , L. J. Gillespie , D. O. Frimannsson , J. M. Kelly , T. Gunnlaugsson , Chem. Soc. Rev. 2013, 42, 1601. 23325367 10.1039/c2cs35467e

[26] Titration of **1** with Zn(ClO_4_)_2_⋅6 H_2_O (Figure S55) gave a similar change, confirming that this is the result of Zn^2+^ coordination to the bipyridine.

[27] Selected examples in which the selectivity of sensors for Cd^2+^ over Zn^2+^ is investigated:

[27a] H. Yuasa , N. Miyagawa , T. Izumi , M. Nakatani , M. Izumi , H. Hashimoto , Org. Lett. 2004, 6, 1489; 15101774 10.1021/ol049628v

[27b] Z. Zhong , Y. Zhao , Org. Lett. 2007, 9, 2891; 17585772 10.1021/ol071130g

[27c] S. Huang , R. J. Clark , L. Zhu , Org. Lett. 2007, 9, 4999; 17956110 10.1021/ol702208y

[27d] S. Y. Park , J. H. Yoon , C. S. Hong , R. Souane , J. S. Kim , S. E. Matthews , J. Vicens , J. Org. Chem. 2008, 73, 8212; 18817447 10.1021/jo8012918

[27e] Z. Xu , K.-H. Baek , H. N. Kim , J. Cui , X. Qian , D. R. Spring , I. Shin , J. Yoon , J. Am. Chem. Soc. 2010, 132, 601; 20000765 10.1021/ja907334j

[27f] K. Jobe , C. H. Brennan , M. Motevalli , S. M. Goldup , M. Watkinson , Chem. Commun. 2011, 47, 6036; 10.1039/c1cc11213a 21528144

[27g] X. Zhou , P. Li , Z. Shi , X. Tang , C. Chen , W. Liu , Inorg. Chem. 2012, 51, 9226; 22905728 10.1021/ic300661c

[27h] H. Mehdi , W. Gong , H. Guo , M. Watkinson , H. Ma , A. Wajahat , G. Ning , Chem. Eur. J. 2017, 23, 13067. 28612518 10.1002/chem.201701948

[28] C−H⋅⋅⋅N interactions are commonly observed in rotaxanes synthesized using the AT-CuAAC reaction with bipyridine macrocycles. See Refs [22].

[29] Similar coordination mode with Zn^2+^: J. D. Crowley , K. D. Hänni , D. A. Leigh , A. M. Z. Slawin , J. Am. Chem. Soc. 2010, 132, 5309. 20334379 10.1021/ja101029u

[30] UV/Vis values allow comparison with macrocycle **1** Fluorescence titration gave *K* _d_=1.09×10^−7^ m ^−1^ with a lower goodness of fit (R^2^=98.2 %; Figures S49,50). The discrepancy may be due to the water associated with the metal salt; the same procedure in the presence of 2 % H_2_O gave comparable values for *K* _d_ (Figures S51-2).

[31] **4**, **5**, and **6** exhibit similar or weaker binding with Zn^2+^ than **1** In the case of **4**, this be caused by the sterically crowded binding motif. The weaker binding of **5** and **6** probably reflects the N−H⋅⋅⋅N H-bond interactions (implied by ^1^H NMR) to be overcome in binding the metal ion.

[32] Radii given are for 6 coordinate metal ions: F. A. Cotton , G. Wilkinson , Advanced Inorganic Chemistry, 5th ed., Wiley, New York, 1988.

[33a] Y. H. Lau , P. J. Rutledge , M. Watkinson , M. H. Todd , Chem. Soc. Rev. 2011, 40, 2848; 21380414 10.1039/c0cs00143k

[33b] L. N. Neupane , J. M. Kim , C. R. Lohani , K.-H. Lee , J. Mater. Chem. 2012, 22, 4003;

[33c] Y. Wu , Y. Dong , J. Li , X. Huang , Y. Cheng , C. Zhu , Chem. Asian J. 2011, 6, 2725; 22043499 10.1002/asia.201100534

[33d] H. F. Wang , S. P. Wu , Tetrahedron 2013, 69, 1965.

