# Supplementary material for: Chelating Rotaxane Ligands as Fluorescent Sensors for Metal Ions
Source: Angew Chem Int Ed Engl. 2018 Apr 6;57(19):5310–4. doi: 10.1002/anie.201712931 (PMC5947674; doi:10.1002/anie.201712931)
Supplement: Supplementary file 1 — Supplementary [file ANIE-57-5310-s001.pdf]

Angewandte Chemie
Eine Zeitschrift der Gesellschaft Deutscher Chemiker

Supporting Information

Chelating Rotaxane Ligands as Fluorescent Sensors for Metal Ions

Mathieu Denis, Jessica Pancholi, Kajally Jobe, Michael Watkinson, and Stephen M. Goldup**

anie_201712931_sm_miscellaneous_information.pdf

Contents

1. General Experimental 2
2. General procedure for the synthesis of rotaxanes 3
3. Synthesis of Rotaxane 4 3
4. Synthesis of Rotaxane 5 9
5. Synthesis of Rotaxane 6 16
6. Synthesis of Rotaxane 7 26
7. NMR stack plots 35
8. UV-vis / Fluorescence titrations 38
9. X-ray Data 48
10. References 50

1. General Experimental

Unless otherwise stated, all reagents and anhydrous solvents were purchased from commercial sources and used without further purification. All reactions were carried out under an atmosphere of N_2 using anhydrous solvents unless otherwise stated. Petrol refers to the fraction of petroleum ether boiling in the range 40-60 °C. DIPEA refers to *N*,*N*-diisopropylethylamine. NH_3-EDTA solution refers to an aqueous solution of NH_3 (17% w/w) with sodium-ethylenediaminetetraacetate (0.1 M). Flash column chromatography was performed using a Biotage Isolera-4 automated chromatography system, employing Biotage SNAP or ZIP cartridges. Analytical TLC was performed on precoated silica gel plates (0.25 mm thick, 60F254, Merck, Germany) and observed under UV light. NMR spectra were recorded on Bruker AV400, AV3-400, AV500 or Bruker AV600 instrument, at a constant temperature of 298 K. Chemical shifts are reported in parts per million from low to high field and referenced to residual solvent. Coupling constants are reported in Hertz. Standard abbreviations indicating multiplicity were used as follows: m = multiplet, quint = quintet, q = quartet, t = triplet, d = doublet, s = singlet, app. = apparent, br = broad. ^{13}C data were typically collected as phased JMOD experiments. Signal assignment was carried out using 2D NMR methods (HSQC, HMBC, COSY, NOESY) where necessary. In the case of some complex multiplets with contributions from more than one signal, absolute assignment was not possible, hence indicative assignments (e.g., H_A or H_B) are provided. All melting points were determined using a Griffin apparatus and are uncorrected. Low resolution mass spectrometry was carried out either by the mass spectrometry services at the Queen Mary University of London using an Agilent SL Ion Trap MSD instrument or at the University of Southampton using a Waters TQD mass spectrometer equipped with a triple quadrupole analyser with UHPLC injection (BEH C18 column, acetonitrile/hexane gradient with 0.2% formic acid). High resolution mass spectrometry (HRMS) was carried out either by the EPSRC UK National Mass Spectrometry Facility (NMSF) Swansea University or by the mass spectrometry services at the University of Southampton with samples analysed using a MaXis (Bruker Daltonics) with a Time of Flight (TOF) analyser; samples were introduced into the mass spectrometer via a Dionex Ultimate 3000 autosampler and a UHPLC pump using a 20-100% acetonitrile/hexane gradient with 0.2% formic acid over 5 min at 0.6 mL/min (column: Acquity UPLC BEH C18 (Waters) 1.7 μm 50 × 2.1 mm).

The following compounds were synthesised according to literature procedures:
Macrocycle **1**,[1] ethynyl-3,5-di-*tert*-butylbenzene (**2**),[2] and rotaxane **S15**.[3]

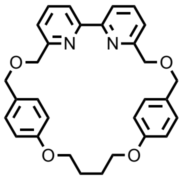

1

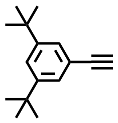

2

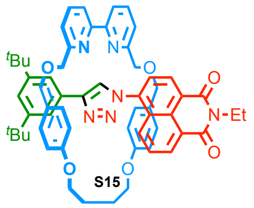

2. General procedure for the synthesis of rotaxanes

The macrocycle **1** (1 eq.), azide (1 eq.), alkyne **2** (1 eq.), $[Cu(MeCN)_4]PF_6$ (0.96 eq.) and DIPEA (1 eq.) were weighed dry into a sealed flask and purged with N_2. EtOH was added to make a 0.02 M solution (wrt. macrocycle), and the mixture stirred at r.t. for 16 h. The solvent was evaporated, and the resulting residue diluted with CH_2Cl_2 and washed with 16% aqueous EDTA tetrasodium-saturated ammonia solution. The organic layer was retained and the aqueous layer extracted twice further with CH_2Cl_2. The organic extracts were combined, dried over $MgSO_4$, filtered and dried *in vacuo*. The crude mixture was purified by flash column chromatography (1:1 Petrol/ CH_2Cl_2 to 10% MeCN/1:1 Petrol-CH_2Cl_2) to yield the desired [2]rotaxane.

3. Synthesis of Rotaxane 4

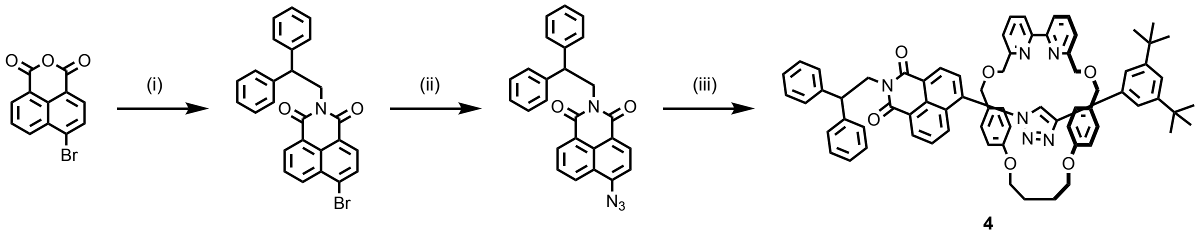

S1 S2 4

Scheme S1 Synthesis of rotaxane **4**. Conditions: (i) diphenyl ethylamine, EtOH, reflux, 18 h, 97%. (ii) NaN_3, NMP, r.t., 24 h, 59%. (iii) **1**, **2**, $[Cu(MeCN)_4]PF_6$, $^iPr_2EtN$, EtOH, r.t., 16 h, 40%.

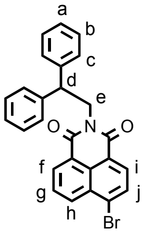

6-Bromo-2-(2,2-diphenylethyl)-1H-benzo[de]isoquinoline-1,3(2H)-dione (S1)

4-Bromo-1,8-naphthalic anhydride (1.5 g, 5.4 mmol) was dissolved in EtOH (45 mL). Diphenyl ethylamine (1.3 g, 6.5 mmol) was added to the solution and refluxed at 80 °C for 18 h. After cooling the reaction mixture to r.t., and then on ice, the resulting precipitate was collected by suction filtration and dried *in vacuo* to give bromide **S1** as a light brown solid (2.4 g, 97%). ^{1}H NMR ($CDCl_3$, 400 MHz, 298 K) δ 8.56 (dd, J = 7.4, 0.8, 1H, H_f) 8.52 (dd, J = 8.4, 0.8, 1H, H_h), 8.32 (d, J = 7.8, 1H, H_i), 7.99 (d, J = 7.8, 1H, H_j), 7.79 (dd, J = 8.4, 7.4, 1H, H_g), 7.38-7.32 (m, 4H, H_c), 7.28-7.20 (m, 4H, H_b), 7.17-7.12 (m, 2H, H_c), 4.88-4.78 (m, 3H, H_d and H_e). ^{13}C NMR ($CDCl_3$, 101 MHz, 298 K) δ 163.6, 163.6, 141.6, 133.2, 132.0, 131.2, 131.0, 130.5, 130.2, 128.9, 128.5, 128.4, 128.0 126.7, 122.9, 122.0, 48.7, 44.6. IR: (ν_{max}/cm^{-1}) 3120, 1704, 1650, 1570, 1320, 1009, 960, 740. M.p. (°C) 136-140. HRMS (ESI+) m/z = 478.0419 $[M+Na]^+$ (calc. for $C_{26}H_{18}NNaO_2$ 478.0413). UV: λ_{max}(MeCN)/nm (ε / mol^{-1}cm^{-1}dm^3) 341 (11395).

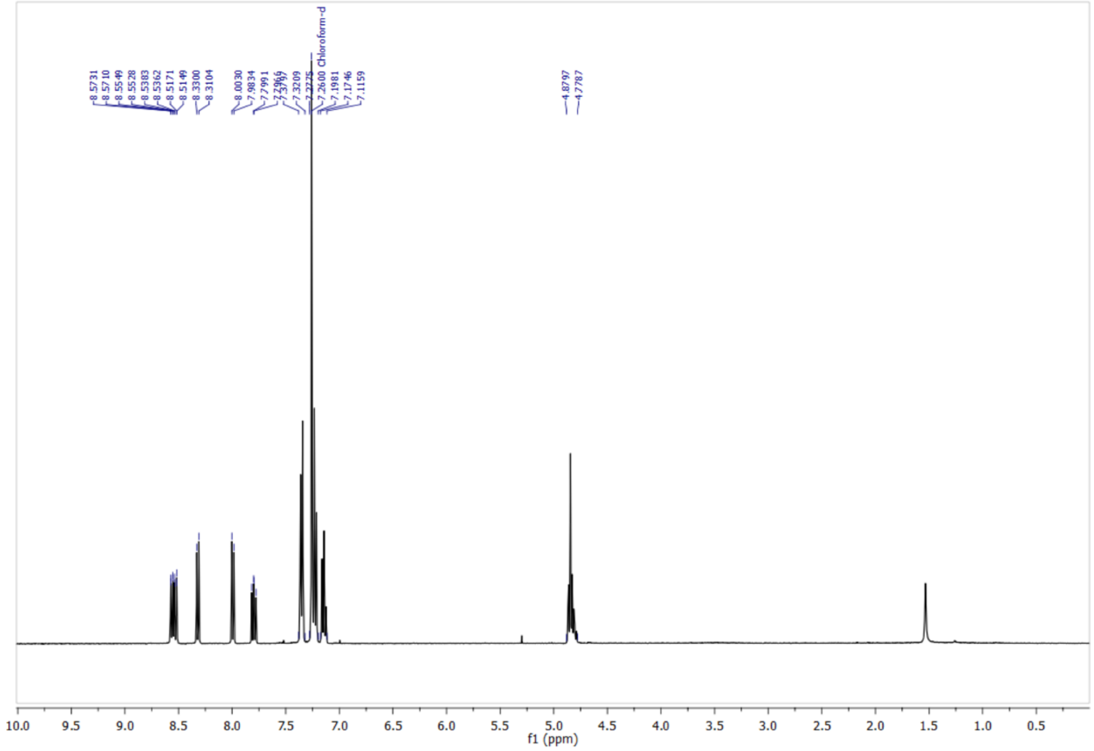

Figure S1 ^{1}H NMR ($CDCl_3$, 400 MHz, 298 K) of **S1**.

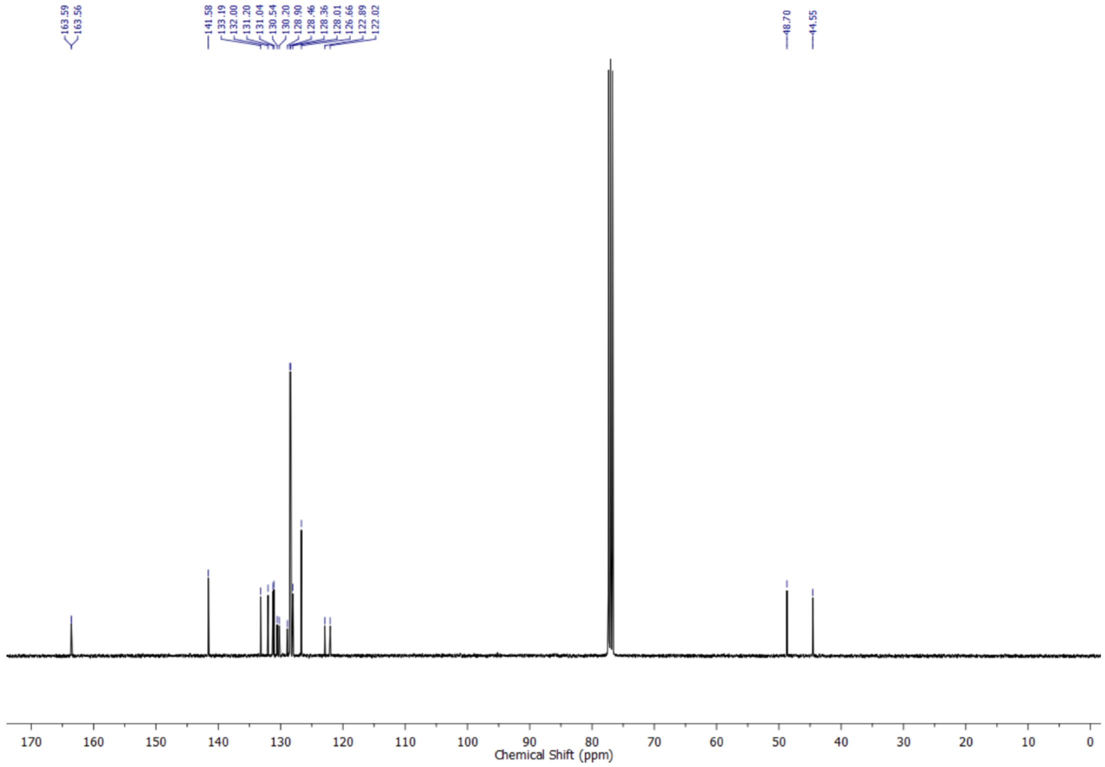

Figure S2 ^{13}C NMR ($CDCl_3$, 101 MHz, 298 K) of **S1**.

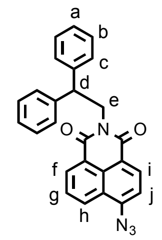

6-Azido-2-(2,2-diphenylethyl)-1H-benzo[de]isoquinoline-1,3(2H)-dione (S2)

Bromide **S1** (0.82 g, 1.8 mmol) was stirred with NaN_3 (0.23 g, 3.6 mmol) in NMP (20 mL) at r.t. for 24 h. After reducing the volume to ~5 mL *in vacuo*, the reaction was diluted with EtOAc (30 mL) and washed with brine (3 x 30 mL). The organic layer was dried with $MgSO_4$, filtered and concentrated *in vacuo*. The crude product was purified by flash column chromatography (8:2 Petrol/EtOAc) to yield azide **S2** as a brown solid (0.45 g, 59%). ^{1}H NMR ($CDCl_3$, 400 MHz, 298 K) δ 8.54 (dd, J = 7.3, 1.2, 1H, H_f), 8.49 (d, J = 8.0, 1H, H_i), 8.39 (dd, J = 8.4, 1.2, 1H, H_h), 7.69 (dd, J = 8.4, 7.3, 1H, H_g), 7.42 (d, J = 8.0, 1H, H_j), 7.38-7.33 (m, 4H, H_c), 7.27-7.20 (m, 4H, H_b), 7.17-7.11 (m, 2H, H_a), 4.87-4.78 (m, 3H, H_d and H_e). ^{13}C NMR ($CDCl_3$, 101 MHz, 298 K) δ 164.0, 163.6, 143.4, 141.7, 132.2, 131.7, 129.1, 128.7, 128.5, 128.3, 126.8, 126.6, 124.3, 122.4, 118.7, 114.6, 48.8, 44.5. IR: (ν_{max}/cm^{-1}) 3140, 2923, 2110, 1650, 1560, 1290, 1233, 1046. M.p. (°C) 162-165. HRMS (EI) m/z = 491.1121 $[M+Na]^+$ (calc. for $C_{26}H_{18}N_4NaO_2$ 491.1133). UV: λ_{max}(MeCN)/nm (ε / mol^{-1}cm^{-1}dm^3) 364 (3937).

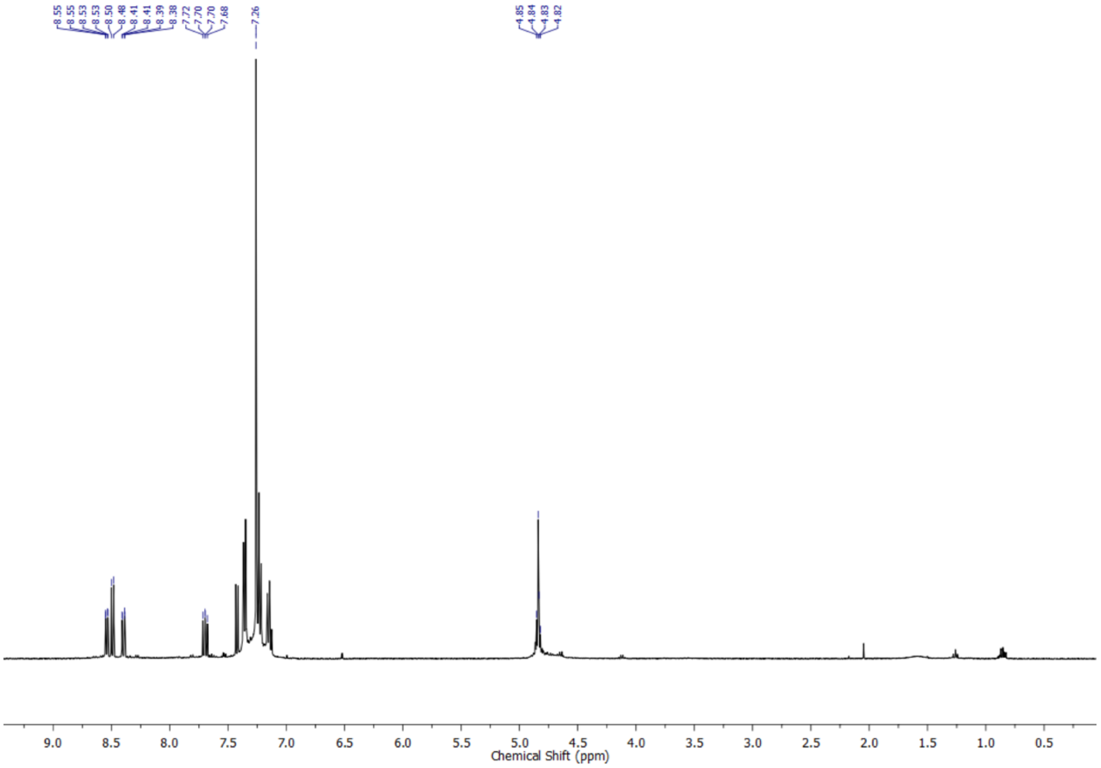

Figure S3 ^{1}H NMR ($CDCl_3$, 400 MHz, 298 K) of **S2**.

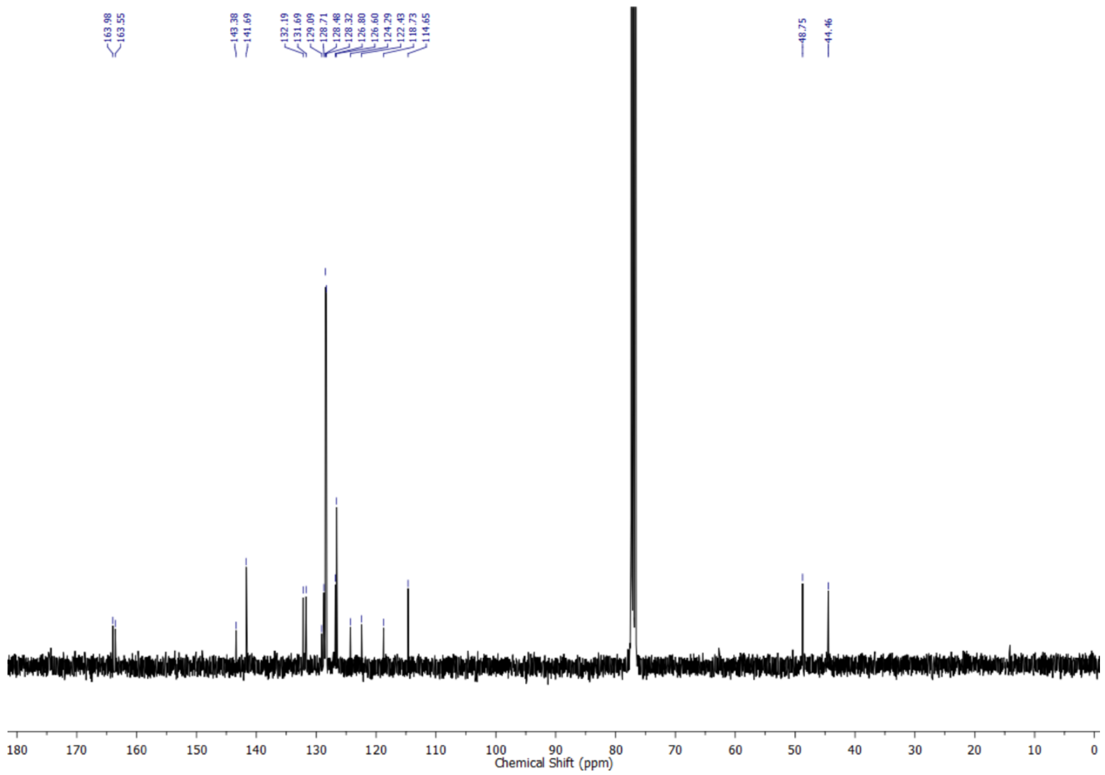

Figure S4 ^{13}C NMR ($CDCl_3$, 101 MHz, 298 K) of **S2**.

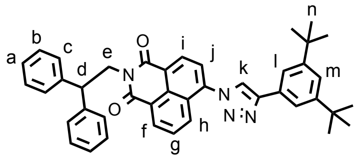

Axle (S3)

Azide **S2** (20 mg, 0.05 mmol), alkyne **2** (12 mg, 0.05 mmol) and $[Cu(MeCN)_4]PF_6$ (3.7 mg, 0.01 mmol) were weighed dry into a sealed flask and purged with N_2. CH_2Cl_2 (0.5 mL) was added, followed by DIPEA (1 drop) and the mixture stirred at r.t. for 4 h. The solvent was diluted with CH_2Cl_2 (2 mL) and washed with 16% aqueous EDTA tetrasodium-saturated ammonia solution (2.5 mL). The organic layer was retained and the aqueous layer extracted twice further with CH_2Cl_2. The organic extracts were combined, dried over $MgSO_4$, filtered and dried *in vacuo*. The crude mixture was purified by flash column chromatography (1:1 Petrol/CH_2Cl_2 to 5% EA/1:1 Petrol-CH_2Cl_2) to yield the product **S3** as a pale orange solid (12 mg, 40%).^{1}H NMR ($CDCl_3$, 400 MHz, 298 K) δ 8.65 – 8.59 (m, 2H, H_j and H_f), 8.29 (dd, *J* = 8.6, 1.1 Hz, 1H, H_h), 8.22 (s, 1H, H_k), 7.86 (d, *J* = 7.7, 1H, H_j), 7.84 – 7.77 (m, 3H, H_g and H_l), 7.50 (t, *J* = 1.8, 1H, H_m), 7.41 – 7.33 (m, 4H, H_c), 7.30 – 7.21 (m, 4H, H_b), 7.21 – 7.13 (m, 2H, H_a), 4.94 – 4.87 (m, 2H, H_e), 4.73 (app dd, *J* = 9.3, 6.8, 1H, H_d), 1.31 (s, 18H, H_n). ^{13}C NMR ($CDCl_3$, 101 MHz, 298 K) δ 163.8, 163.2, 151.9, 149.5, 141.6, 138.4, 132.4, 130.8, 129.7, 129.1, 129.0, 128.7, 128.6, 128.5, 126.9, 126.7, 123.8, 123.6, 123.3, 122.9, 121.9, 120.5, 48.8, 44.8, 35.2, 31.6. HRMS (ESI+) m/z = 633.3226 $[M+H]^+$ (calc. for $C_{42}H_{41}N_4O_2$ 633.3224).

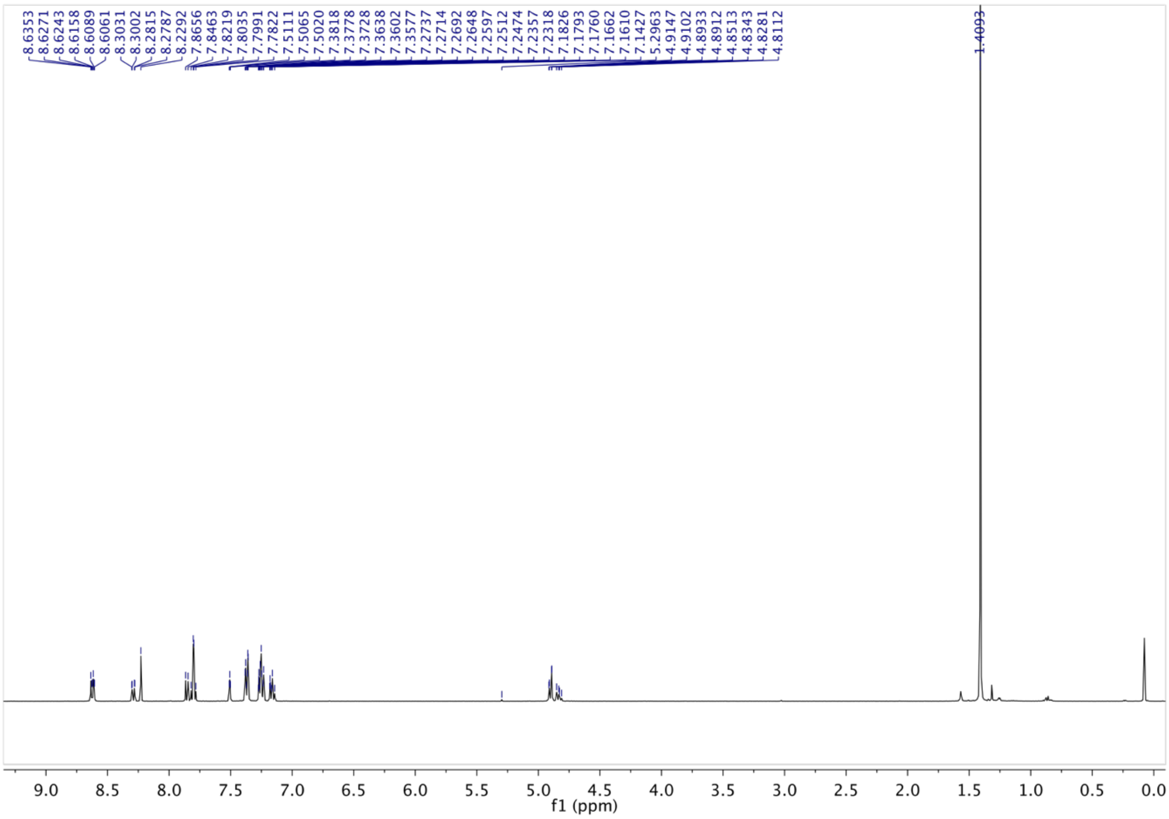

Figure S5 ^{1}H NMR ($CDCl_3$, 400 MHz, 298 K) of **S3**.

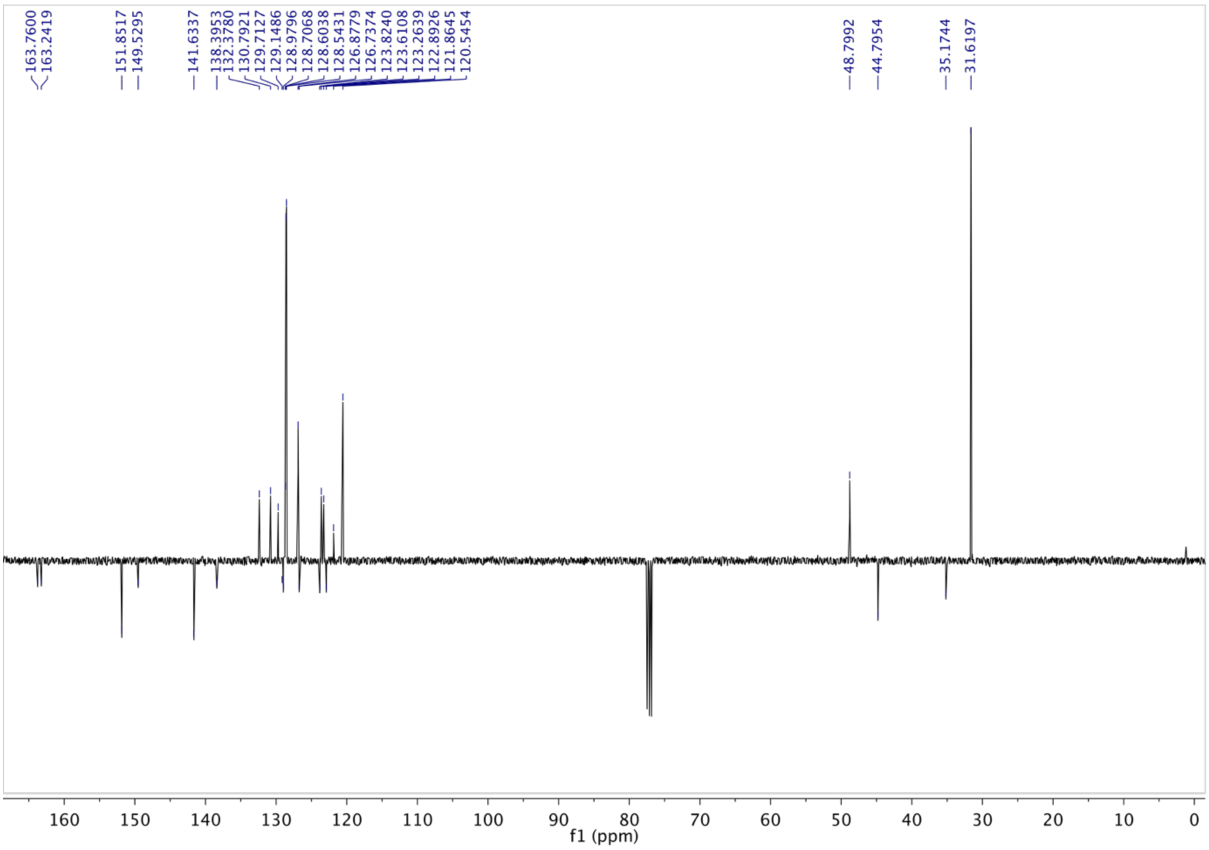

Figure S6 JMOD NMR ($CDCl_3$, 101 MHz, 298 K) of **S3**.

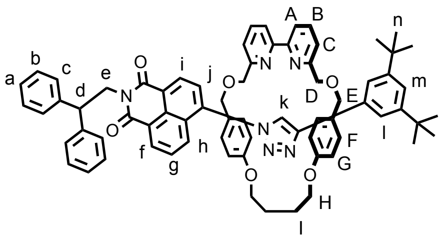

Rotaxane (4)

General procedure was employed with macrocycle **1** (0.20 g, 0.41 mmol) acetylene **2** (0.09 g, 0.41 mmol), azide **S2** (0.19 g, 0.41 mmol), $[Cu(MeCN)_4]PF_6$ (0.15 g, 0.40 mmol), and DIPEA (72 µL, 0.41 mmol) in EtOH (20 mL). Purification as noted gave rotaxane **4** as a light brown foam (0.41 g, 88%). ^{1}H NMR ($CDCl_3$, 400 MHz, 298 K) δ 10.30 (s, 1H, H_k), 8.43 (dd, J = 7.3, 1.0, 1H, H_f), 8.21 (dd, J = 8.5, 1.0, 1H, H_h), 8.13 (d, J = 7.9, 1H, H_i), 7.84 (d, J = 1.8, 2H, H_l), 7.52 (dd, J = 8.5, 7.3, 1H, H_g), 7.46-7.36 (m, 7H, H_h, H_B and H_c), 7.31 (t, J = 1.8,1H, H_m), 7.29-7.23 (m, 6H, two of H_A/H_C and H_b), 7.20-7.14 (m, 4H, two of H_A/H_C and H_a), 6.71 (d, J = 8.5, 4H, H_F), 6.56 (d, J = 8.5, 4H, H_G), 4.88-4.82 (m, 3H, H_d and H_e), 4.57 (d, J = 12.1, 2H, two of H_E), 4.47 (ap. t, J = 6.6, 4H, H_H), 4.16 (d, J = 12.1, 2H, two of H_E), 3.97 (d, J = 12.3, 2H, two of H_D), 3.90 (d, J = 12.3, 2H, two of H_D), 2.40-2.30 (m, 2H, two of H_I), 2.25-2.13 (m, 2H, two of H_I), 1.32 (s, 18H, H_n). ^{13}C NMR ($CDCl_3$, 101 MHz, 298 K) δ 164.9, 164.4, 158.9, 158.7, 155.6, 151.4, 142.3, 137.8, 130.6, 129.1, 128.7, 128.3, 126.5, 122.2, 121.2, 120.1, 115.0, 49.0, 44.2, 42.5, 35.0, 31.5, 24.8. IR: (ν_{max}/cm^{-1}) 2965, 2902, 2840, 2347, 1713, 1679, 1598, 1253, 1079, 990. M.p. (°C) 45-50. HRMS (EI) m/z = 1115.5429 $[M+H]^+$ (calc. for $C_{72}H_{71}N_6O_6$ 1115.5430. UV: λ_{max}(MeCN)/nm (ε / mol^{-1}cm^{-1}dm^3) 345 (9874).

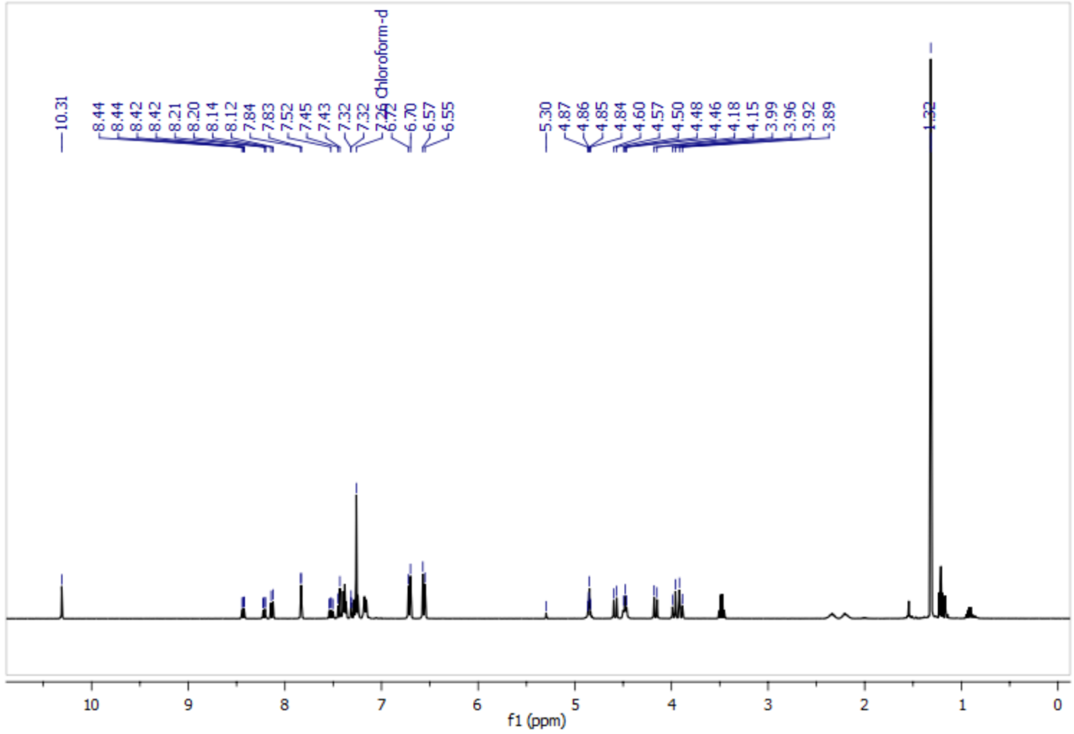

Figure S7 ^{1}H NMR ($CDCl_3$, 400 MHz, 298 K) of **4**.

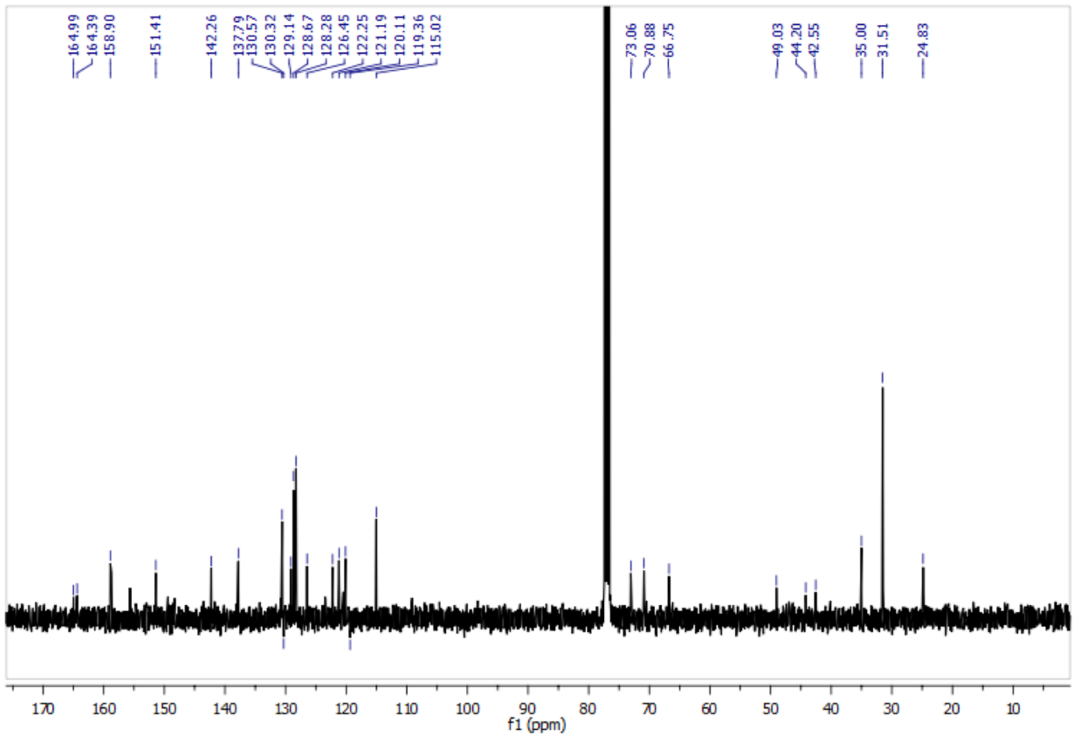

Figure S8 ^{13}C NMR ($CDCl_3$, 101 MHz, 298 K) of **4**.

4. Synthesis of Rotaxane 5

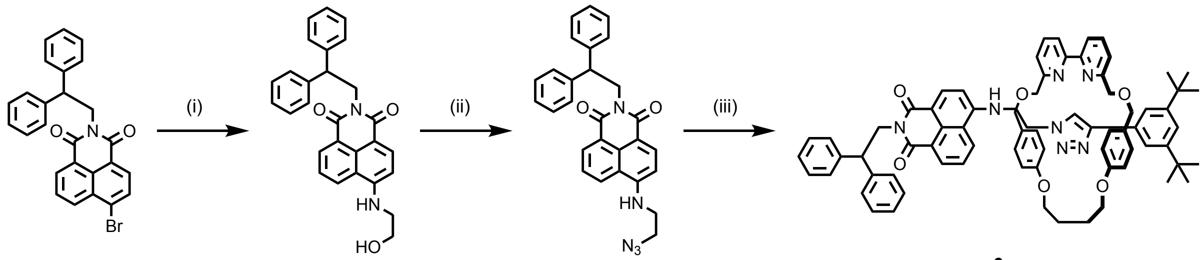

Scheme S2 Synthesis of rotaxane **5**. Conditions: (i) ethanolamine, Cu_2O, K_2CO_3, DMSO, 90 °C, 16 h, 96%. (ii) diphenyl phosphoryl azide, DEAD, PPh_3, THF, r.t., 2 h, 61%. (iii) **1**, **2**, $[Cu(MeCN)_4]PF_6$, $^{i}Pr_2EtN$, EtOH, r.t., 16 h, 72%.

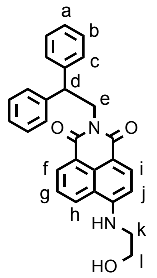

2-(2,2-diphenylethyl)-6-((2-hydroxyethyl)amino)-1H-benzo[de]isoquinoline-1,3(2H)-dione (S4)

To a solution of bromide **S1** (0.3 g, 0.64 mmol) in DMSO (10 mL) was added Cu_2O (1.8 mg, 0.013 mmol), K_2CO_3 (18.0 mg, 0.13 mmol) and ethanolamine (0.39 mL, 6.4 mmol), and was heated at 90 °C overnight. The reaction mixture was diluted with brine (40 mL), and the aqueous portion extracted with EtOAc

(2 x 40 mL). The organic phase was dried with $MgSO_4$, filtered and concentrated *in vacuo*. The resulting oil was purified by flash column chromatography (CH_2Cl_2 to 7:3 CH_2Cl_2/EtOAc) to give alcohol **S4** as an orange foam (0.27 g, 96%). ^1H NMR ($CDCl_3$, 400 MHz, 298 K) δ 8.21 (d, *J* = 7.2, 1H, H_f), 8.14 (d, *J* = 8.4, 1H, H_i), 7.56 (d, *J* = 8.4, 1H, H_h), 7.35-7.30 (m, 4H, H_c), 7.27 (ap. t, *J* = 7.8, 1H, H_g), 7.20-7.14 (m, 4H, H_b), 7.09-7.03 (m, 2H, H_a), 6.44 (d, *J* = 8.4, 1H, H_j), 5.43 (bs, 1H, -*NH*-), 4.83-4.78 (m, 1H, H_d), 4.75-4.69 (m, 2H, H_e), 3.94 (t, *J* = 4.9, 2H, H_l), 3.41 (br t, *J* = 4.4, 2H, H_k). ^{13}C NMR ($CDCl_3$, 101 MHz, 298 K) δ 164.7, 164.4, 149.4, 142.2, 134.3, 131.2, 129.5, 128.7, 128.5, 126.7, 125.8, 124.7, 122.7, 120.1, 110.2, 104.4, 60.6, 49.1, 45.6, 44.5. IR: (ν_{max}/cm^{-1}) 3505, 2950, 2350, 1699, 1635, 1589, 1380, 1340, 1180. M.p. (°C) 43-45. HRMS (ESI+) m/z = 437.1856 $[M+H]^+$ (calc. for $C_{28}H_{25}N_2O_3$ 437.1860). UV: λ_{max}(MeCN)/nm (ε / mol^{-1}cm^{-1}dm^3) 432 (2347).

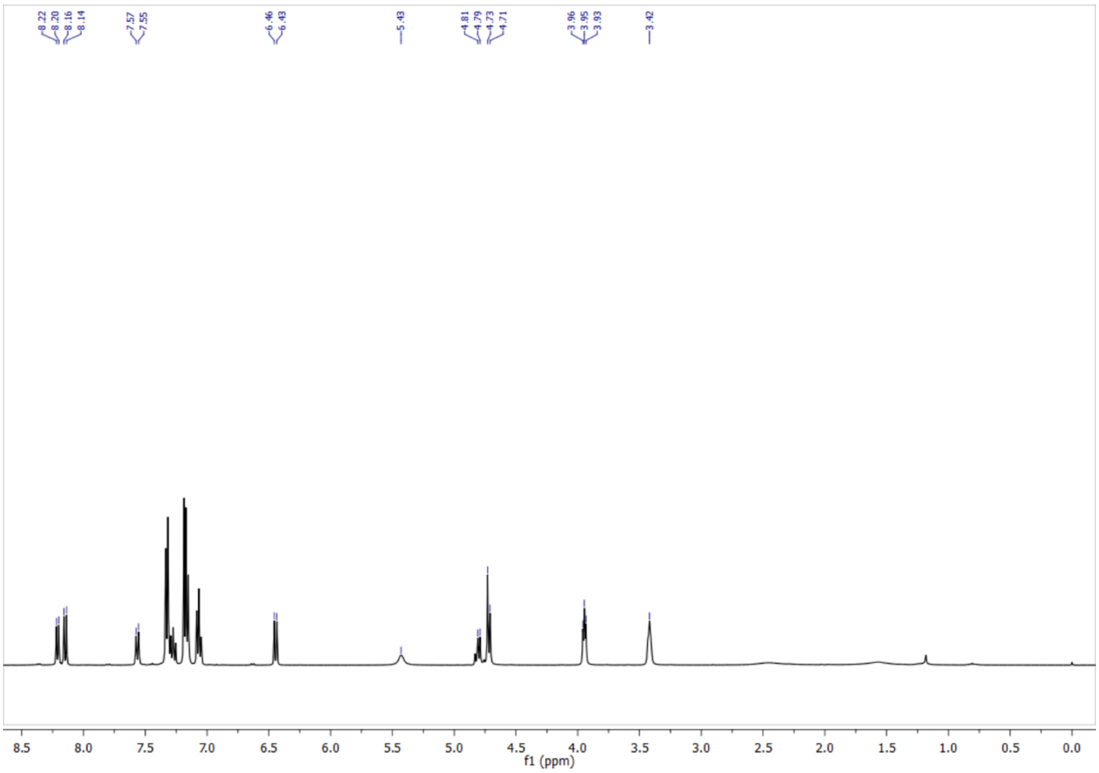

Figure S9 ^1H NMR ($CDCl_3$, 400 MHz, 298 K) of **S4**.

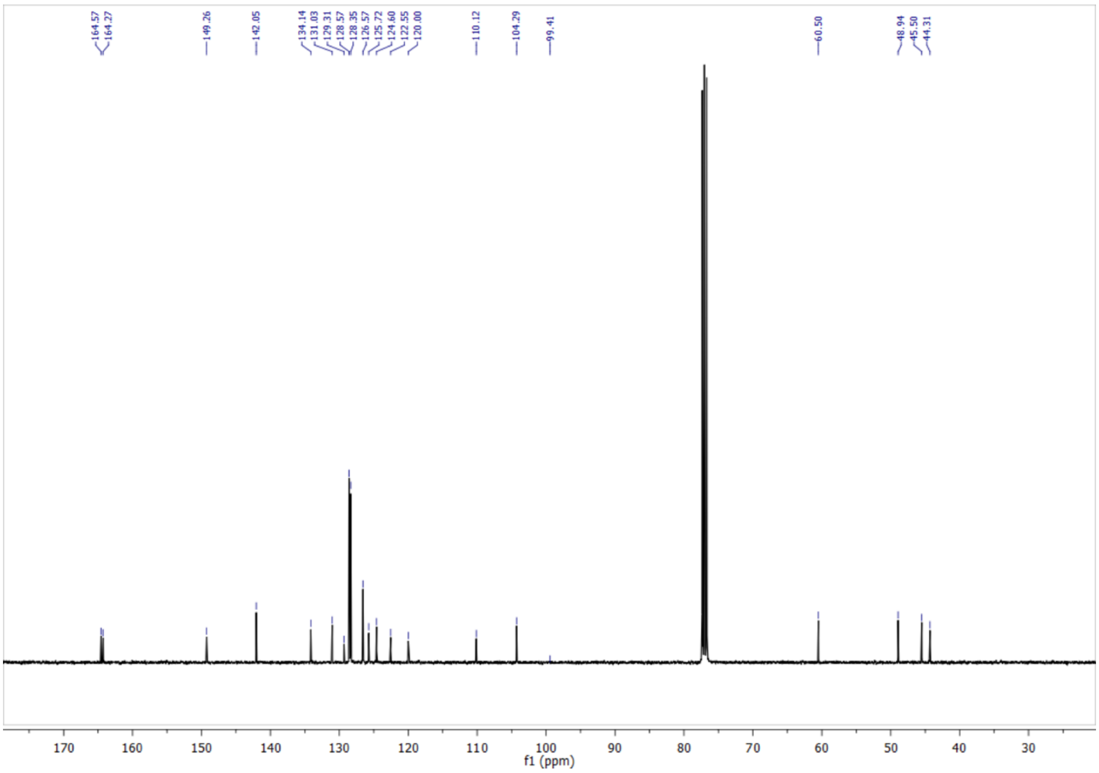

Figure S10 ^{13}C NMR ($CDCl_3$, 101 MHz, 298 K) of **S4**.

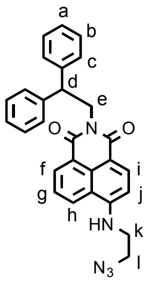

6-((2-azidoethyl)amino)-2-(2,2-diphenylethyl)-1H-benzo[de]isoquinoline-1,3(2H)-dione (S5)

Alcohol **S4** (0.13 g, 0.29 mmol) and triphenylphosphine (0.23 g, 0.87 mmol) were combined in a sealed flask which was purged with N_2. Anhydrous THF (15 mL) was added, and the stirring solution was cooled to 0 °C. DEAD (0.14 mL, 0.87 mmol) was added to the reaction, followed by diphenyl phosphoryl azide (0.20 mL, 0.87 mmol), and stirred for 15 min. The reaction was then stirred at r.t. for 2 h, and then concentrated *in vacuo*. The resulting oil was purified by flash column chromatography (1:1 Petrol/ CH_2Cl_2 to CH_2Cl_2 to 7:3 CH_2Cl_2/EtOAc) to give azide **S5** as an orange solid (0.085 g, 61%). ^{1}H NMR ($CDCl_3$, 400 MHz, 298 K) δ 8.50 (d, *J* = 7.2, 1H, H_f), 8.40 (d, *J* = 8.3, 1H, H_i), 8.05 (d, *J* = 8.4, 1H, H_h), 7.61 (ap. t, *J* = 7.4, 1H, H_g), 7.42-7.34 (m, 4H, H_c), 7.28-7.20 (m, 4H, H_b), 7.16-7.11 (m, 2H, H_a), 6.70 (d, *J* = 8.4, 1H, H_j), 5.39 (br s, 1H, -*NH*-), 4.89-4.80 (m, 3H, H_d and H_e), 3.73 (t, *J* = 5.1, 2H, H_l), 3.65-3.57 (m, 2H, H_k). ^{13}C NMR ($CDCl_3$, 101 MHz, 298 K) δ 164.5, 164.0, 148.4, 142.0, 140.8, 134.0, 131.3, 128.5, 128.3, 126.5, 125.7, 125.1, 123.2, 120.5, 104.5, 49.9, 48.8, 44.3, 42.6. IR: (ν_{max}/cm^{-1}) 3350, 2954, 2101, 1695, 1658, 1580, 1347, 1235, 1120, 1052, 952. M.p. (°C) 90-95. HRMS (ESI+) m/z = 484.1735 $[M+Na]^+$ (calc. for $C_{28}H_{23}N_5NaO$ 484.1744). UV: λ_{max}(MeCN)/nm (ε / mol^{-1}cm^{-1}dm^{3}) 410 (3856).

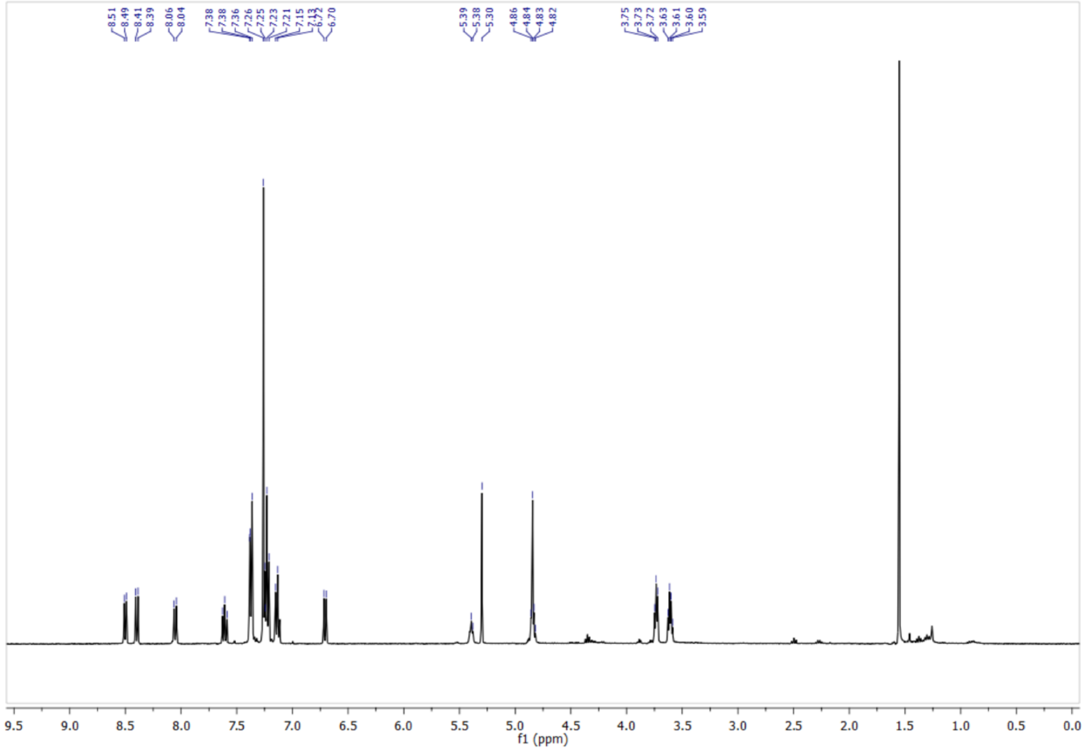

Figure S11 ^{1}H NMR ($CDCl_3$, 400 MHz, 298 K) of **S5**.

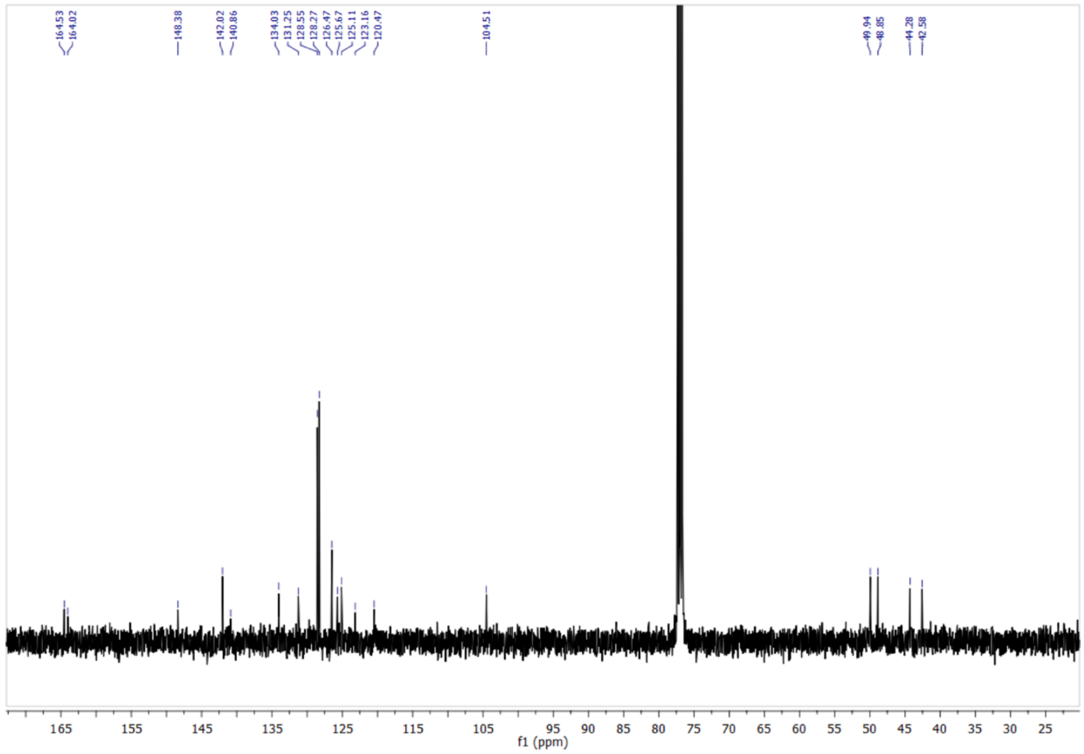

Figure S12 ^{13}C NMR ($CDCl_3$, 101 MHz, 298 K) of **S5**.

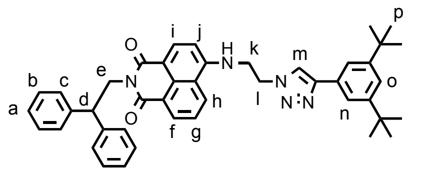

Axle S6

Azide **S5** (26 mg, 0.056 mmol), alkyne **2** (12 mg, 0.056 mmol) and $[Cu(MeCN)_4]PF_6$ (4.2 mg, 0.011 mmol) were weighed dry into a sealed flask and purged with N_2. CH_2Cl_2 (0.5 mL) was added, followed by DIPEA (1 drop) and the mixture stirred at r.t. for 4 h. The solvent was diluted with CH_2Cl_2 (2 mL) and washed with 16% aqueous EDTA tetrasodium-saturated ammonia solution (2.5 mL). The organic layer was retained and the aqueous layer extracted twice further with CH_2Cl_2. The organic extracts were combined, dried over $MgSO_4$, filtered and dried *in vacuo*. The crude mixture was purified by flash column chromatography (1:1 Petrol/CH_2Cl_2 to 10% MeCN/1:1 Petrol-CH_2Cl_2) to yield the product **S6** as a pale yellow residue (32 mg, 70%).^{1}H NMR ($CDCl_3$, 400 MHz, 298 K) δ 8.46 (dd, *J* = 7.3, 1.0, 1H, H_f), 8.38 (d, *J* = 8.3, 1H, H_j), 8.09 (dd, *J* = 8.6, 1.0, 1H, H_h), 7.80 (s, 1H, H_m), 7.61 (d, *J* = 1.8, 2H, H_n), 7.56 (dd, *J* = 8.6, 7.3, 1H, H_g), 7.43 (t, *J* = 1.8, 1H, H_o), 7.40 – 7.32 (m, 4H, H_c), 7.25 – 7.18 (m, 4H, H_b), 7.16 – 7.08 (m, 2H, H_a), 6.69 (d, *J* = 8.4, 1H, H_i), 6.16 (t, *J* = 5.4, 1H, -*NH*-), 4.90 – 4.81 (m, 3H, H_d and H_e), 4.81 – 4.74 (m, 2H, H_l), 3.98 – 3.89 (m, 2H, H_k), 1.34 (s, 18H, H_p). ^{13}C NMR ($CDCl_3$, 101 MHz, 298 K) δ 164.7, 164.2, 151.7, 149.4, 148.4, 142.1, 134.1, 131.5, 129.8, 129.3, 128.7, 128.4, 126.6, 126.3, 125.5, 123.1, 123.0, 120.6, 120.6, 120.4, 111.6, 104.2, 49.0, 48.9, 44.4, 43.4, 35.1, 31.6. HRMS (ESI+) m/z = 676.3636 $[M+H]^+$ (calc. for $C_{44}H_{46}N_5O_2$ 676.3646).

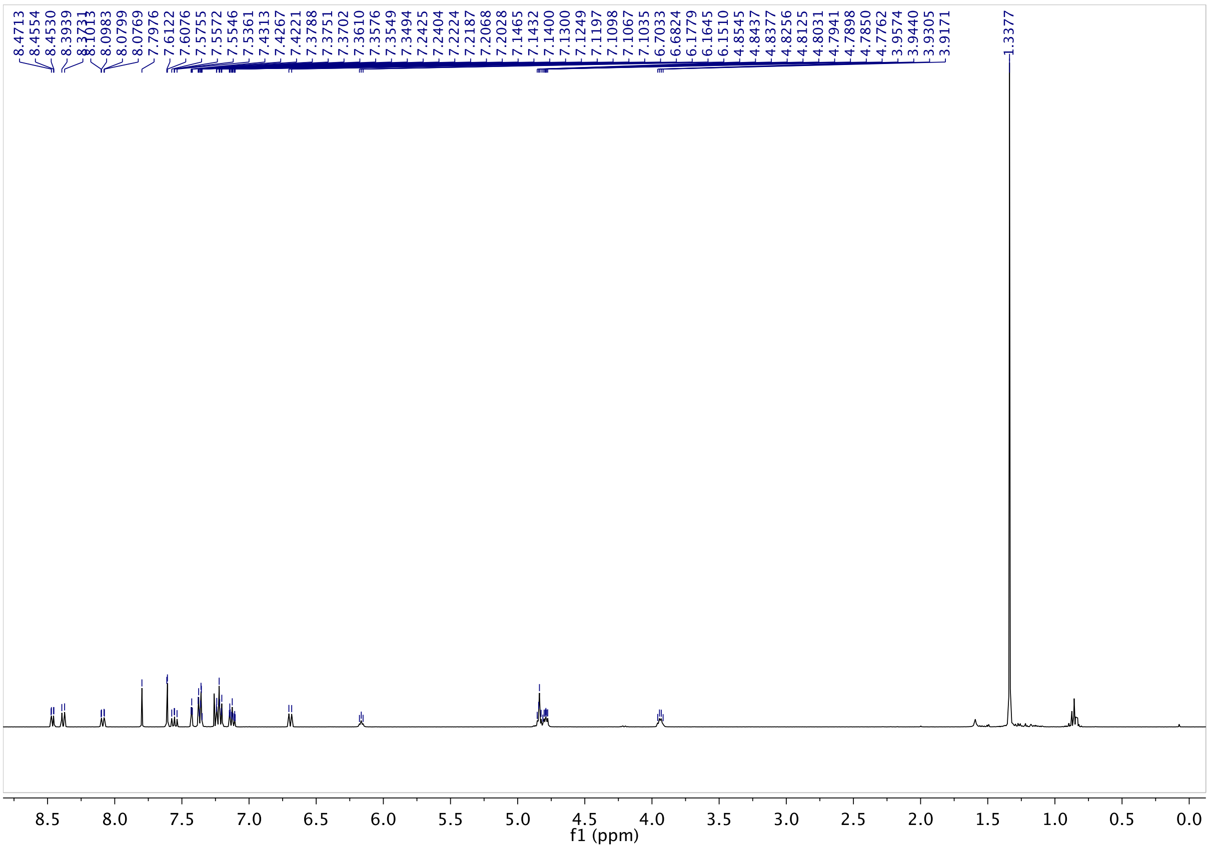

Figure S13 ^{1}H NMR ($CDCl_3$, 400 MHz, 298 K) of **S11**.

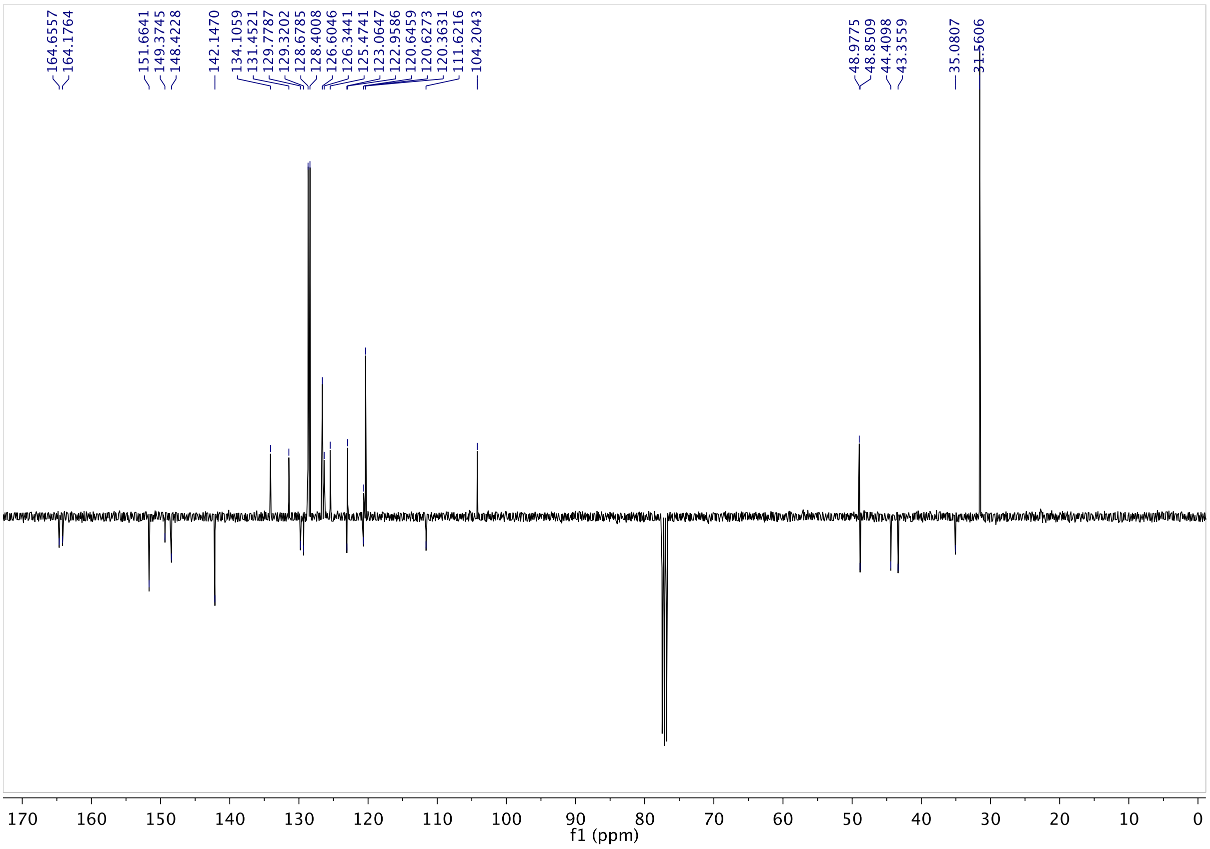

Figure S14 JMOD NMR ($CDCl_3$, 101 MHz, 298 K) of **S11**.

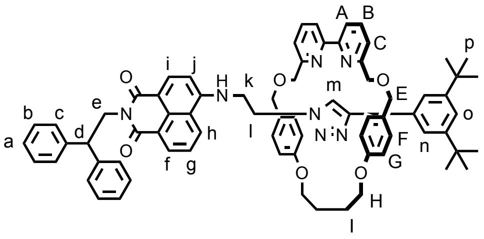

Rotaxane 5

General procedure was employed with macrocycle **1** (0.20 g, 0.41 mmol) acetylene **2** (0.09 g, 0.41 mmol), azide **S5** (0.17 g, 0.41 mmol), $[Cu(MeCN)_4]PF_6$ (0.15 g, 0.40 mmol), and DIPEA (72 µL, 0.41 mmol) in EtOH (20 mL). Purification as noted gave rotaxane **5** as an orange residue (0.35 g, 72%). ^{1}H NMR ($CDCl_3$, 400 MHz, 298 K) δ 8.21-8.13 (m, 2H, H_i and H_f), 7.49-7.43 (m, 3H, H_h and H_B), 7.40-7.36 (m, 2H, H_n), 7.28-7.20 (m, 6H, H_c and two of H_A/H_C), 7.08-7.01 (m, 7H, H_b, H_o and two of H_A/H_C), 6.96-6.91 (m, 2H, H_a), 6.89 (dd, *J* = 8.3, 7.5, 1H, H_g), 6.63 (d, *J* = 8.5, 4H, H_F), 6.40 (d, *J* = 8.5, 4H, H_G), 5.58 (d, *J* = 8.6, 1H, H_j), 4.79-4.73 (m, 1H, H_d), 4.70-4.61 (m, 2H, H_e), 4.36 (d, *J* = 11.8, 2H, two of H_E), 4.11 (d, *J* = 12.0, 2H, two of H_D), 4.02 (d, *J* = 11.8, 2H, two of H_E), 3.90 (d, *J* = 12.0, 2H, two of H_D), 3.89-3.83 (m, 4H, H_H), 3.42-3.35 (m, 2H, H_l), 1.98-1.73 (m, 6H, H_k and H_I), 1.17 (s, 18H, H_p). ^{13}C NMR ($CDCl_3$, 101 MHz, 298 K) δ 159.0, 158.8, 155.8, 151.6, 148.5, 142.4, 137.9, 134.4, 130.7, 130.2, 130.0, 129.3, 128.9, 128.8, 128.4, 126.6, 123.6, 122.5, 122.5, 122.4, 121.4, 120.7, 120.6, 120.3, 115.2, 109.3, 104.7, 73.2, 71.0, 66.9, 49.2, 47.1, 44.3, 42.7, 35.2, 31.7, 24.9. IR: (ν_{max}/cm^{-1}) 2965, 2902, 2840, 2347, 1703, 1660, 1579,

1203, 1191, 1006, 979. HRMS (EI) m/z = 1158.5860 [M+H]$^+$ (calc. for $C_{74}H_{76}N_7O_6$ 1158.5852). UV: λ_{max}(MeCN)/nm (ε / mol^{-1}cm^{-1}dm^3) 436 (15720).

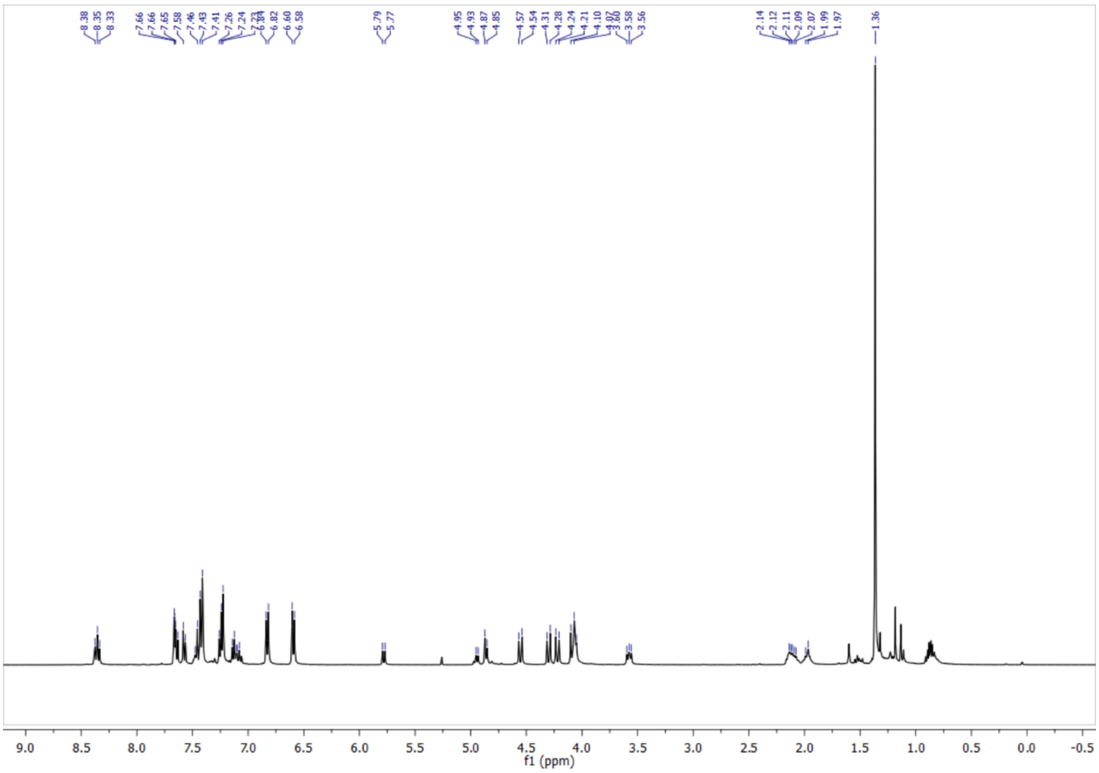

Figure S15 ^{1}H NMR ($CDCl_3$, 400 MHz, 298 K) of **5**.

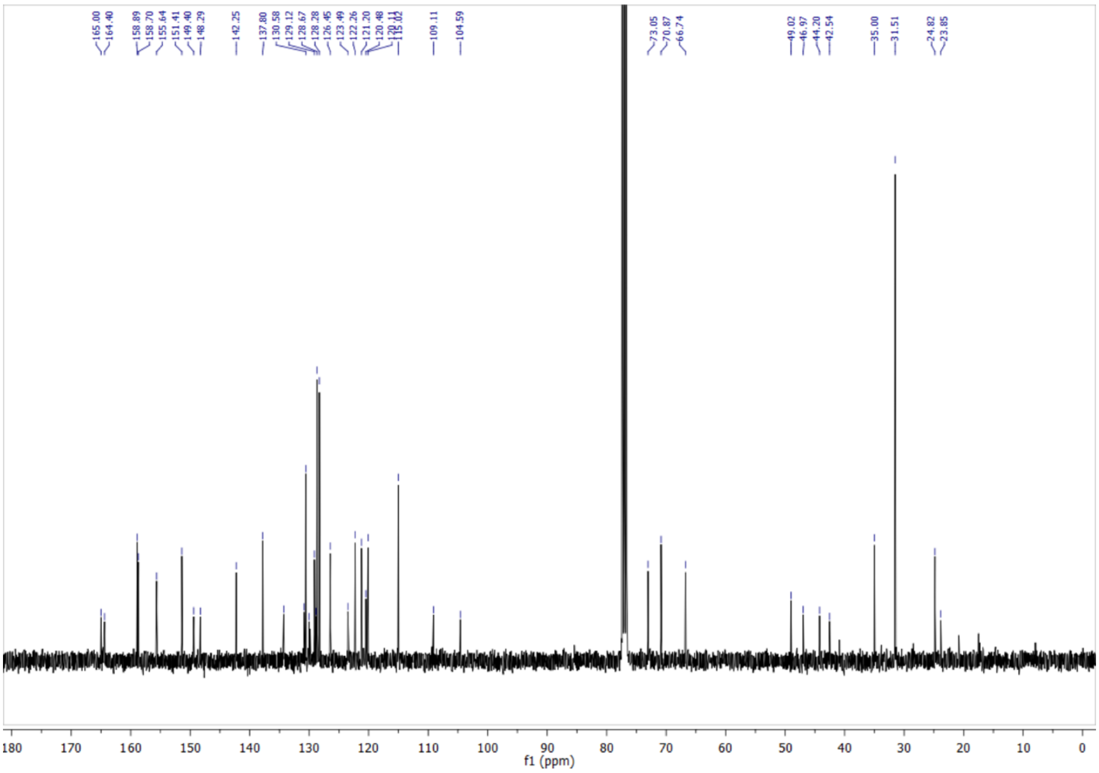

Figure S16 ^{13}C NMR ($CDCl_3$, 101 MHz, 298 K) of **5**.

5. Synthesis of Rotaxane 6

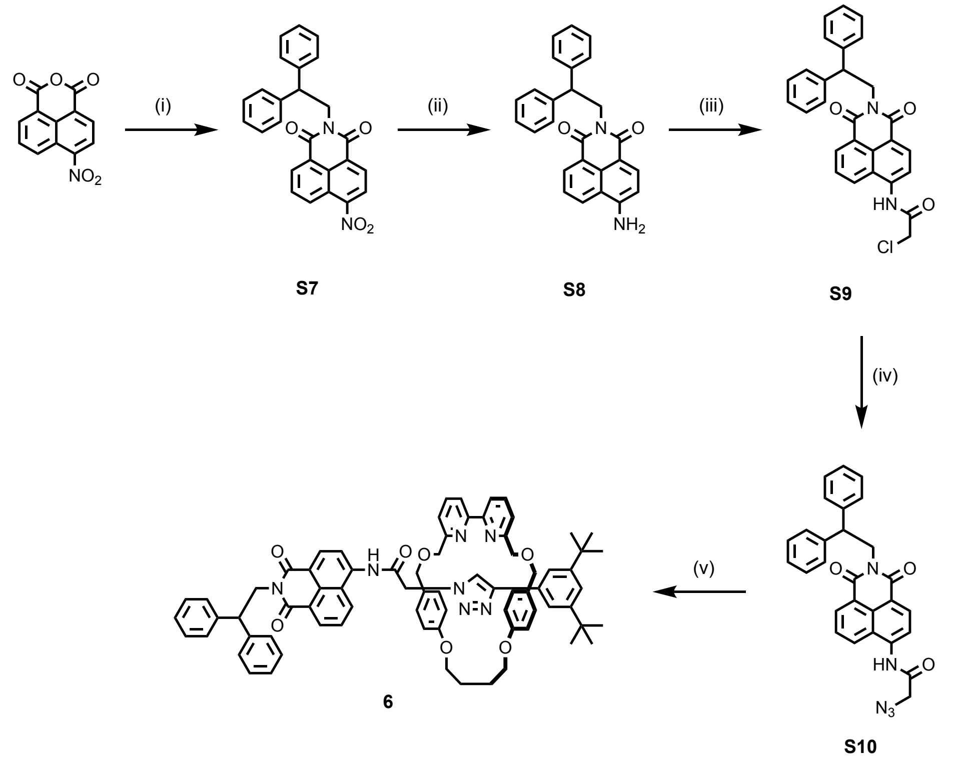

Scheme S3 Synthesis of rotaxane **6**. Conditions: (i) diphenyl ethylamine, EtOH, reflux, 18 h, 86%. (ii) Pd/C, H_2, r.t., 97%. (iii) 2-chloroacetyl chloride, DMAP, THF, r.t., 6 h, 98%. (iv) NaN_3, DMF, 24 h, 84% (v) **1**, **2**, $[Cu(MeCN)_4]PF_6$, $^{i}Pr_2EtN$, EtOH, r.t., 16 h, 60%.

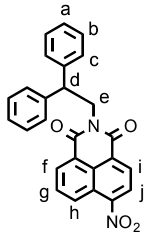

2-(2,2-Diphenylethyl)-6-nitro-1H-benzo[de]isoquinoline-1,3(2H)-dione (S7)

4-Nitro-1,8-naphthalic anhydride (0.5 g, 2.1 mmol) was dissolved in EtOH (15 mL). Diphenyl ethylamine (0.49 g, 2.5 mmol) was added to the solution and refluxed at 80 °C for 18 h. After cooling the reaction mixture to r.t., the solvent was removed *in vacuo* and purified by flash column chromatography (1:1 Petrol/ CH_2Cl_2) to give nitro-compound **S7** as a yellow foam (0.75 g, 86%). ^{1}H NMR ($CDCl_3$, 400 MHz, 298 K) δ 8.79 (dd, *J* = 8.7, 1.0, 1H, H_h), 8.64 (dd, *J* = 7.3, 1.0, 1H, H_f), 8.58 (d, *J* = 8.0, 1H, H_j), 8.34 (d, *J* = 8.0, 1H, H_i), 7.93 (dd, *J* = 8.7, 7.4, 1H, H_g), 7.37-7.32 (m, 4H, H_c), 7.27-7.21 (m, 4H, H_b), 7.18-7.13 (m, 2H, H_a), 4.89-4.85 (m, 2H, H_e), 4.82-4.71 (m, 1H, H_d). ^{13}C NMR ($CDCl_3$, 101 MHz, 298 K) δ 163.3, 162.4, 149.5, 141.3, 132.4, 129.9, 129.7, 129.3, 128.9, 128.4, 128.4, 126.8, 126.7, 123.8, 123.6, 122.8, 48.5, 44.8. IR: (ν_{max}/cm^{-1}) 3405, 2905, 1692, 1620, 1577, 1520, 1375, 1313, 1226, 1181, 989. M.p. (°C) 40-43. LRMS (ESI+) m/z = 423 $[M+H]^+$. UV: λ_{max}(MeCN)/nm (ε / mol^{-1}cm^{-1}dm^{3}) 347 (10978).

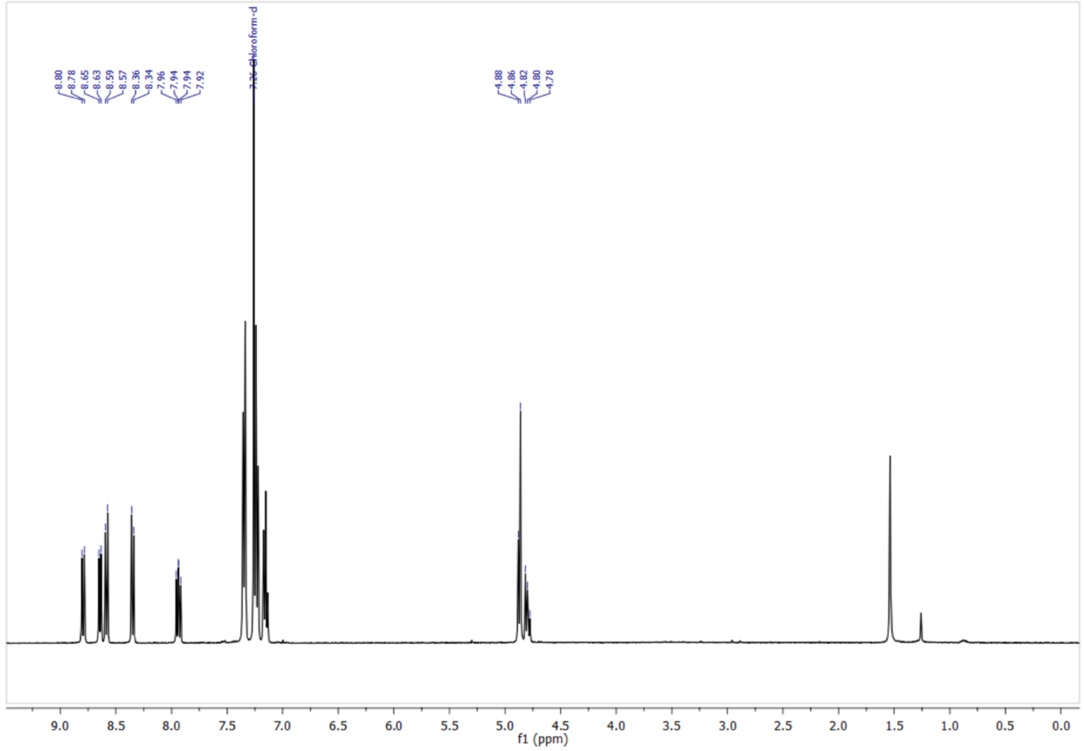

Figure S17 ^{1}H NMR ($CDCl_3$, 400 MHz, 298 K) of **S7**.

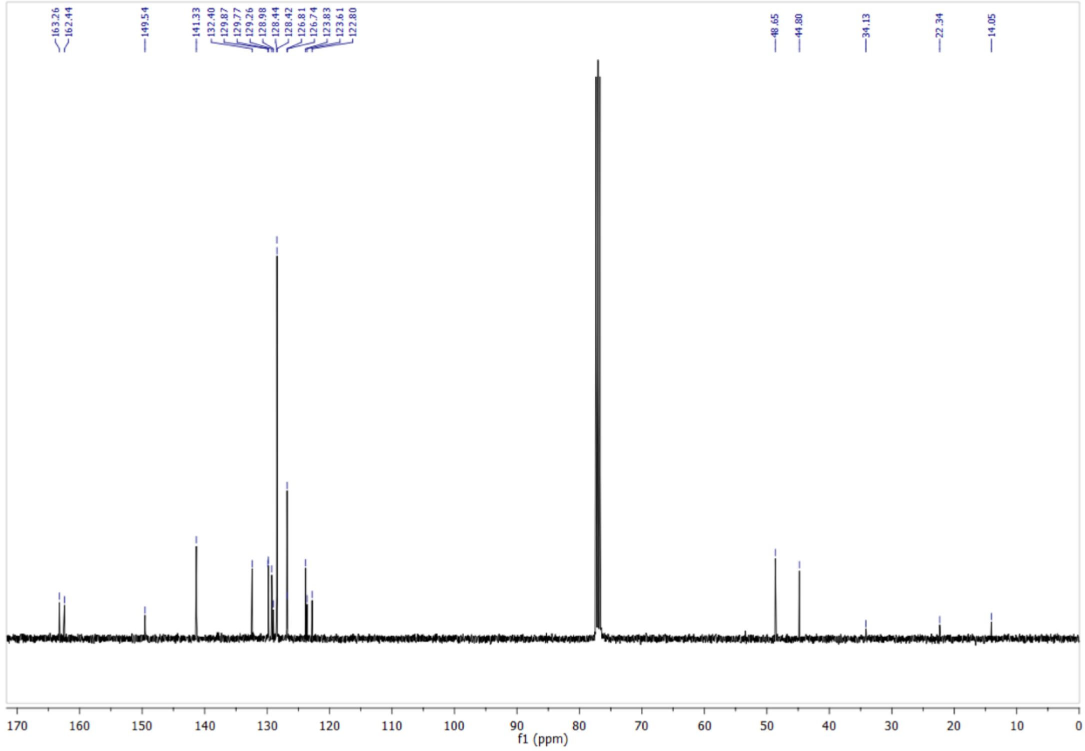

Figure S18 ^{13}C NMR ($CDCl_3$, 101 MHz, 298 K) of **S7**.

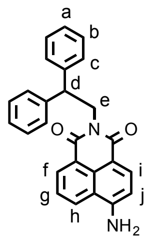

2-(2,2-diphenylethyl)-6-amino-1H-benzo[de]isoquinoline-1,3(2H)-dione (S8)

Nitro-compound **S7** (0.36 g, 0.95 mmol) was hydrogenated in MeOH/EtOAc (1:1, 20 mL) at r.t. under a hydrogen atmosphere, using a Pd/C catalyst (5%wt, 0.20 g, 0.095 mmol). The reaction was monitored by TLC until all starting material had been consumed (~4 h). The reaction mixture was filtered through Celite, washed with MeOH/EtOAc (1:1), and the solvent evaporated *in vacuo* to yield aniline **S8** as a yellow solid (0.36 g, 97%). ^{1}H NMR ((CD_3)$_2$SO, 400 MHz, 298 K) δ 8.57 (dd, *J* = 8.4, 1.1, 1H, H_h), 8.34 (dd, *J* = 7.3, 1.1, 1H, H_f), 8.13 (d, *J* = 8.4, 1H, H_i), 7.60 (dd, *J* = 8.4, 7.3, 1H, H_g), 7.42 (br s, 1H, -NH_2-), 7.35-7.30 (m, 4H, H_c), 7.26-7.20 (m, 4H, H_b), 7.16-7.11 (m, 2H, H_a), 6.80 (d, *J* = 8.4, 1H, H_j), 4.73-4.69 (m, 3H, H_d and H_e).^{13}C NMR ((CD_3)$_2$SO, 101 MHz, 298 K) δ 163.8, 162.9, 152.6, 142.2, 133.9, 130.9, 129.6, 129.3, 128.2, 128.0, 126.4, 123.9, 121.6, 119.2, 108.1, 107.3, 48.4, 43.3. IR: (ν_{max}/cm^{-1}) 3505, 3349, 3225, 2358, 1648, 1592, 1375, 1246, 1017. 982. M.p. (°C) 95-96. HRMS (ESI+) m/z = 393.1599 [M+H$^+$] (calc. for $C_{26}H_{21}N_2O_2$ 393.1598). UV: λ_{max}(MeCN)/nm (ε / mol^{-1}cm^{-1}dm^3) 428 (3226).

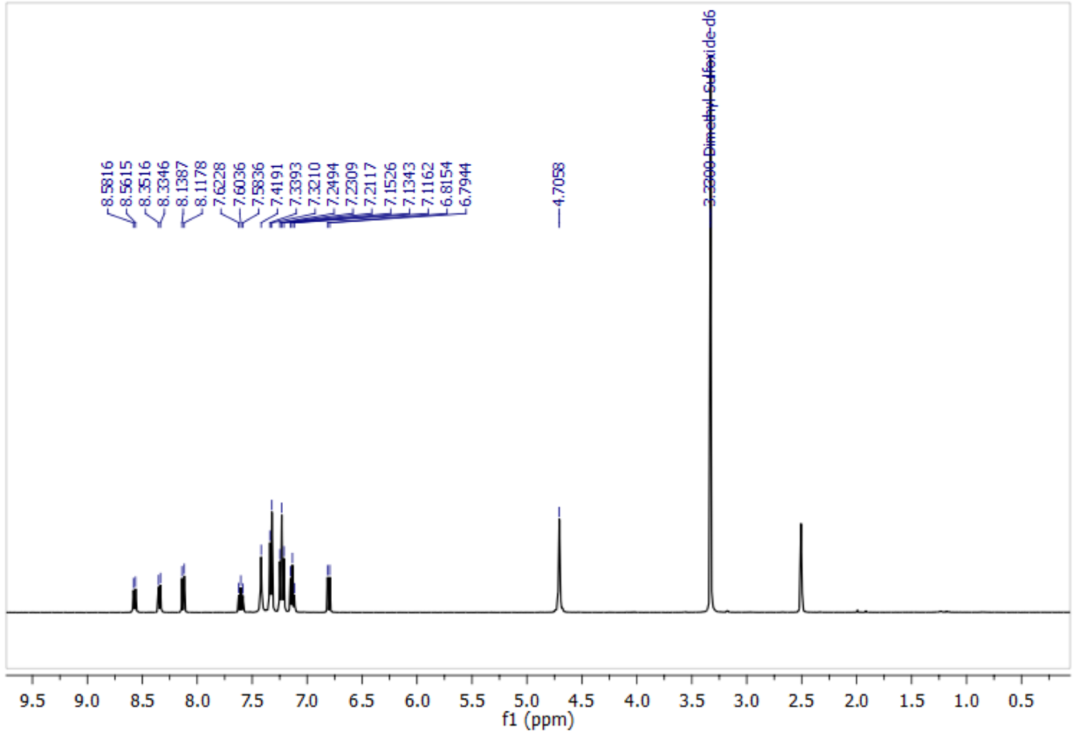

Figure S19 ^{1}H NMR ((CD_3)$_2$SO, 400 MHz, 298 K) of **S8**.

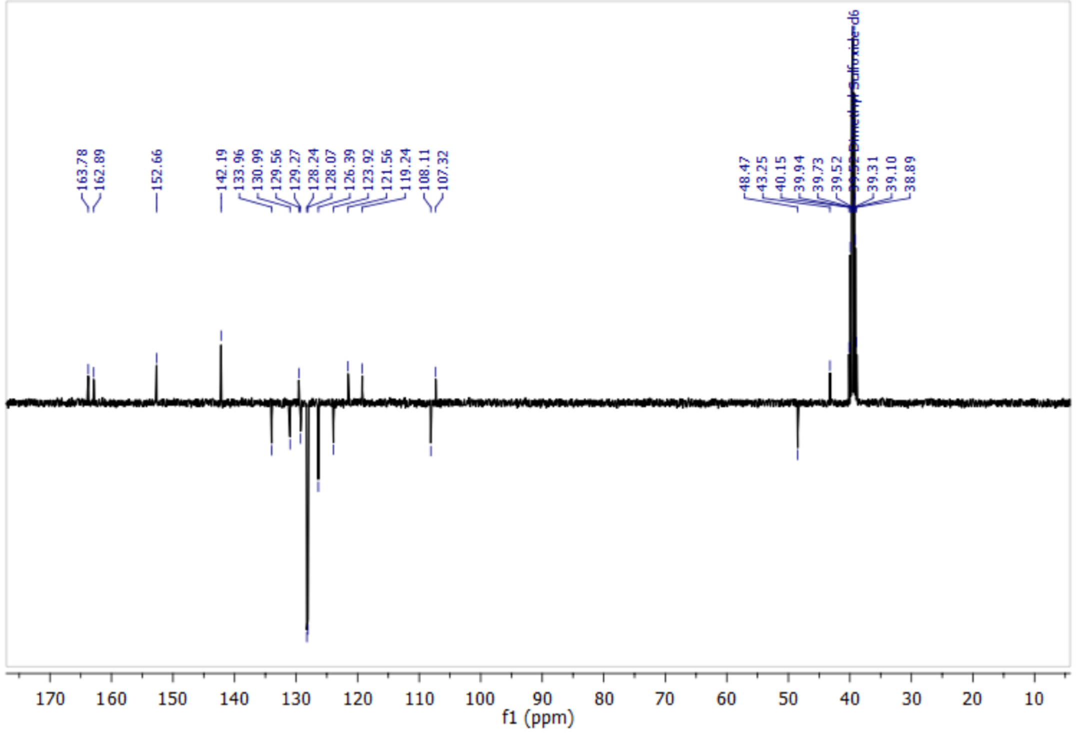

Figure S20 JMOD NMR ($(CD_3)_2SO$, 101 MHz, 298 K) of **S8**.

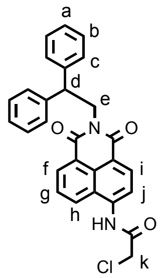

2-Chloro-N-(2-(2,2-diphenylethyl)-1,3-dioxo-2,3-dihydro-1H-benzo[de]isoquinolin-6-yl)acetamide (S9)

Aniline **S8** (0.36 g, 0.92 mmol) and DMAP (56 mg, 0.46 mmol) were combined in a sealed flask which was purged with N_2. Anhydrous THF (15 mL) was added, and the stirring solution was cooled to 0 °C. A solution of 2-chloroacetyl chloride (0.12 mL, 1.4 mmol) in anhydrous THF (10 mL) was added to the cold stirring solution dropwise over 20 min. The solution was then stirred at r.t. for 6 h. The reaction mixture was diluted with H_2O (30 mL) and stirred vigorously, then extracted with EtOAc (3 x 30 mL). The combined organic phase was washed with brine (30 mL) and saturated aqueous $NaHCO_3$ (2 x 30 mL), dried using $MgSO_4$, filtered and concentrated *in vacuo* to yield amide **S9** without further purification as a yellow solid (0.42 g, 98%). ^{1}H NMR ($CDCl_3$, 400 MHz, 298 K) δ 9.08 (s, 1H, -*NH*-), 8.56-8.50 (m, 2H, H_f and H_i), 8.40 (d, *J* = 8.1, 1H, H_j), 8.13 (dd, *J* = 8.5, 0.7, 1H, H_h), 7.76 (dd, *J* = 8.4, 7.4, 1H, H_g), 7.38-7.33 (m, 4H, H_c), 7.26-7.20 (m, 4H, H_b), 7.17-7.11 (m, 2H, H_a), 4.88-4.80 (m, 3H, H_d and H_e), 4.37 (s, 2H, H_k). ^{13}C NMR ($CDCl_3$, 101 MHz, 298 K) δ 164.2, 163.9, 163.4, 141.7, 137.0, 132.1, 131.3, 128.7, 128.5, 128.4, 127.2, 126.6, 125.7, 123.8, 123.3, 119.5, 119.1, 48.7, 44.5, 43.4. IR: (ν_{max}/cm^{-1}) 3465, 3202, 2965, 2345, 1729, 1690, 1594, 1565, 1293, 1203, 1101, 979. M.p. (°C) 139-144. HRMS

(ESI+) m/z = 469.1324 [M+H$^+$] (calc. for $C_{28}H_{22}ClN_2O_3$ 469.1313). UV: λ_{max}(MeCN)/nm (ε / mol^{-1}cm^{-1}dm^3) 360 (10571).

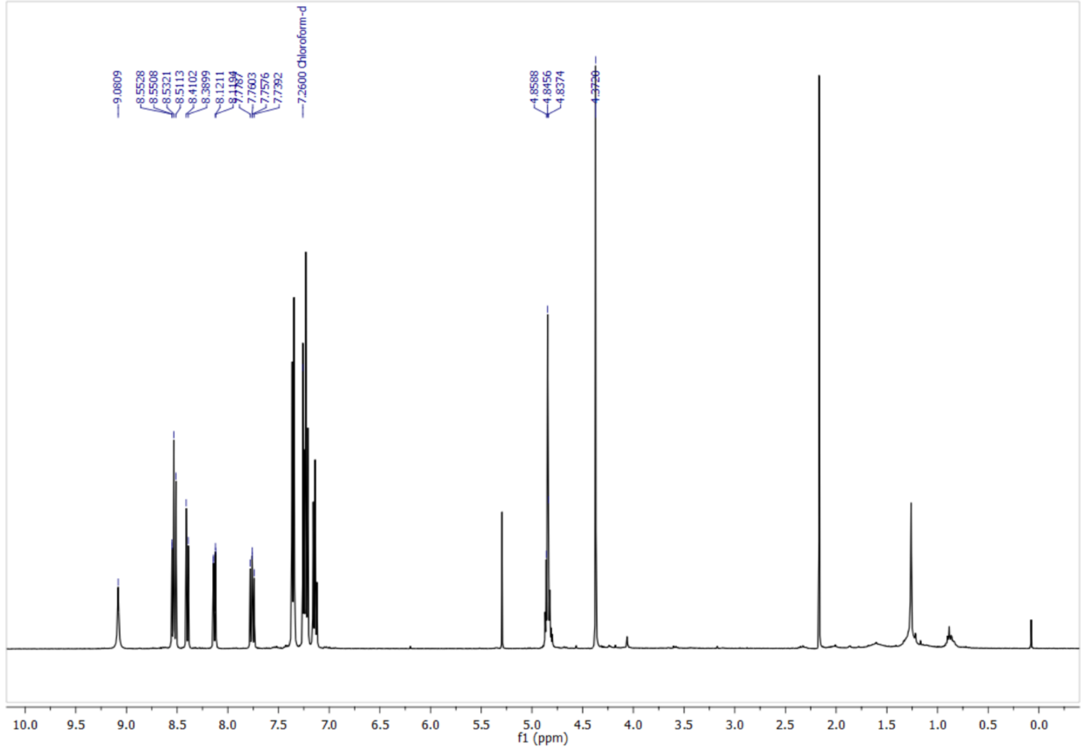

Figure S21 ^{1}H NMR ($CDCl_3$, 400 MHz, 298 K) of **S9**.

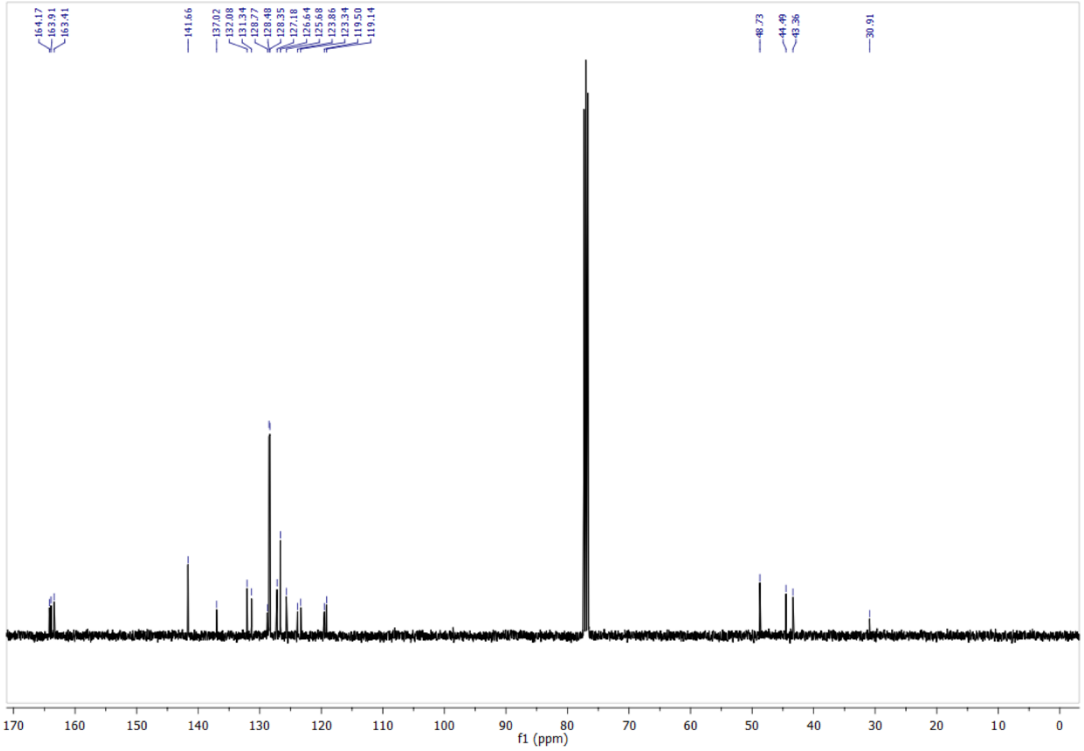

Figure S22 ^{13}C NMR ($CDCl_3$, 101 MHz, 298 K) of **S9**.

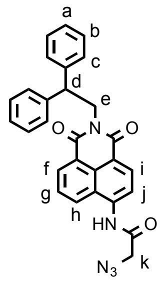

2-Azido-N-(2-(2,2-diphenylethyl)-1,3-dioxo-2,3-dihydro-1H-benzo[de]isoquinolin-6-yl)acetamide (S10)

Chloride **S9** (0.4 g, 0.85 mmol) and NaN_3 (0.11 g, 1.71 mmol) were dissolved in DMF (60 mL) and stirred at r.t. for 24 h. The mixture was diluted with brine (100 mL) and the aqueous layer was extracted with EtOAc (3 x 100 mL). The organic phases were combined and washed with saturated aqueous $NaHCO_3$ (150 mL), dried with $MgSO_4$, filtered and concentrated *in vacuo*. The resulting residue was purified by flash column chromatography (9:1 CH_2Cl_2/EtOAc) to give azide **S10** as a brown solid (0.34 g, 84%). ^{1}H NMR ($CDCl_3$, 400 MHz, 298 K) δ 8.87 (s, 1H, -*NH*-), 8.54-8.47 (m, 2H, H_f and H_i), 8.41 (d, J = 8.2, 1H, H_j), 8.13 (d, J = 8.4, 1H, H_h), 7.76 (ap. t, J = 7.8, 1H, H_g), 7.39-7.32 (m, 4H, H_c), 7.27-7.20 (m, 4H, H_b), 7.17-7.10 (m, 2H, H_a), 4.87-4.80 (m, 3H, H_d and H_e), 4.35 (s, 2H, H_k). ^{13}C NMR ($CDCl_3$, 101 MHz, 298 K) δ 164.8, 163.9, 163.4, 141.6, 137.1, 132.1, 131.3, 128.7, 128.5, 128.4, 127.0, 126.6, 125.7, 123.6, 123.3, 119.2, 118.9, 53.2, 48.7, 44.5. IR: (ν_{max}/cm^{-1}) 3460, 3207, 2966, 2339, 2105, 1730, 1696, 1588, 1306, 1212, 1089 M.p. (°C) 146-149. HRMS (EI) m/z = 476.1710 $[M+H]^+$ (calc. for $C_{28}H_{22}N_5O_3$ $[M+H]^+$ 476.1717).UV: λ_{max}(MeCN)/nm (ε / mol^{-1}cm^{-1}dm^3) 361 (10925).

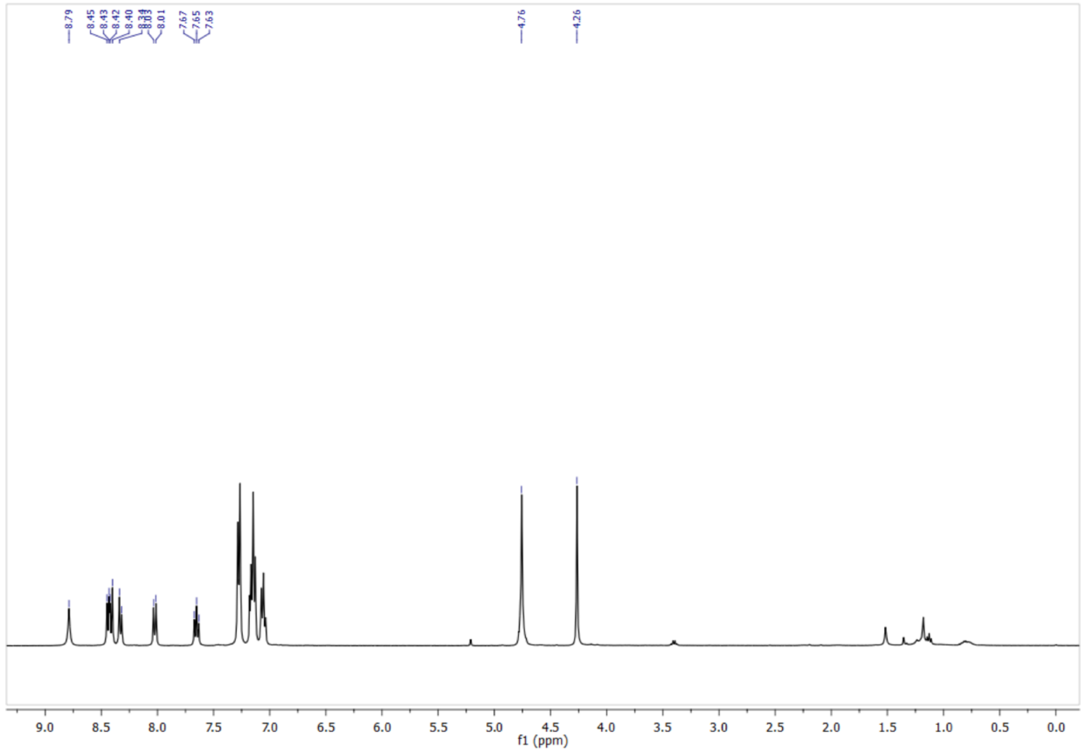

Figure S23 ^{1}H NMR ($CDCl_3$, 400 MHz, 298 K) of **S10**.

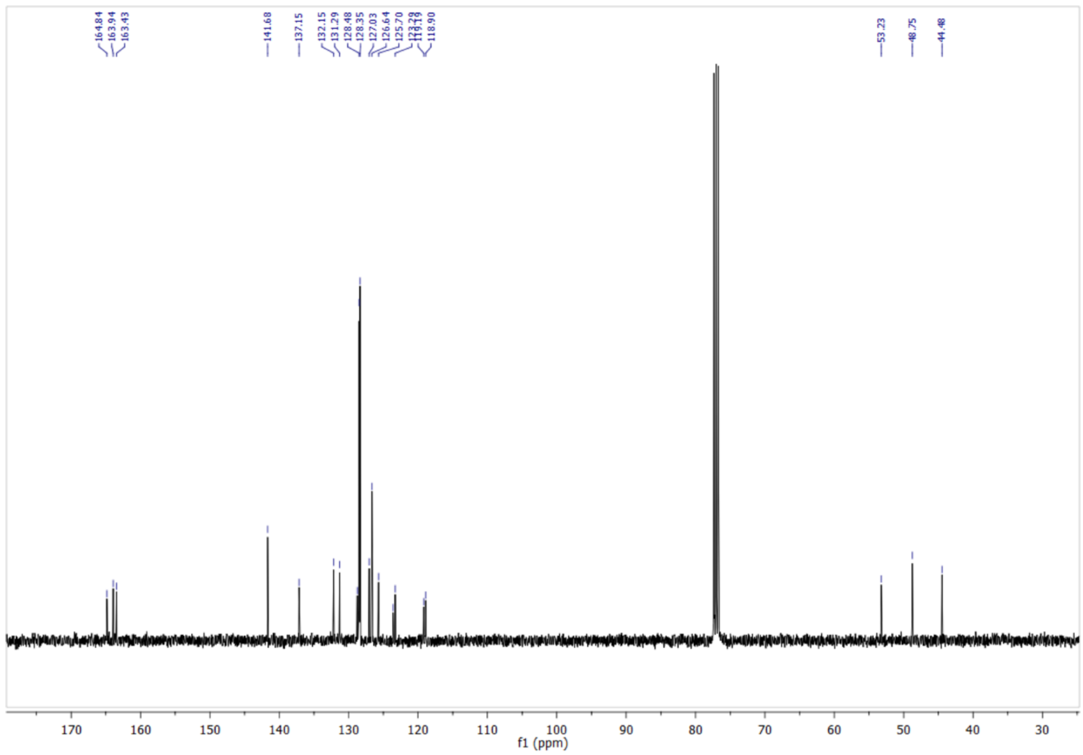

Figure S24 ^{13}C NMR ($CDCl_3$, 101 MHz, 298 K) of **S10**.

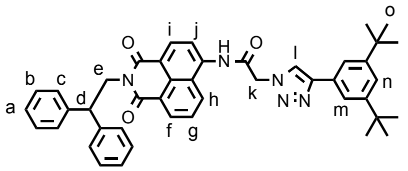

Axle S11

Azide **S10** (44 mg, 0.093 mmol), alkyne **2** (20 mg, 0.093 mmol) and $[Cu(MeCN)_4]PF_6$ (8.7 mg, 0.023 mmol) were weighed dry into a sealed flask and purged with N_2. CH_2Cl_2 (1 mL) was added, followed by DIPEA (1 drop) and the mixture stirred at r.t. for 4 h. The solvent was diluted with CH_2Cl_2 (4 mL) and washed with 16% aqueous EDTA tetrasodium-saturated ammonia solution (5 mL). The organic layer was retained and the aqueous layer extracted twice further with CH_2Cl_2. The organic extracts were combined, dried over $MgSO_4$, filtered and dried *in vacuo*. The crude mixture was purified by flash column chromatography (1:1 Petrol/CH_2Cl_2 to 10% MeCN/1:1 Petrol-CH_2Cl_2) to yield the product **S11** as a yellow residue (57 mg, 89%). ^1H NMR ($CDCl_3$, 400 MHz, 298 K) δ 9.56 (br s, 1H, -NH-), 8.53-8.46 (m, 2H, H_f and H_i), 8.41 (d, J = 8.2, 1H, H_j), 8.12 (d, J = 8.4, 1H, H_h), 8.05 (s, 1H, H_l), 7.73 (ap. t, J = 8.0, 1H, H_g), 7.69 (d, J = 1.7, 2H, H_m), 7.47 (t, J = 1.7, 1H, H_n), 7.37-7.30 (m, 4H, H_c), 7.25-7.18 (m, 4H, H_b), 7.16-7.09 (m, 2H, H_a), 5.43 (s, 2H, H_k), 4.87-4.79 (m, 3H, H_d and H_e), 1.37 (s, 18H, H_p) ^{13}C NMR ($CDCl_3$, 101 MHz, 298 K) δ 164.1, 163.6, 163.6, 151.9, 150.2 (assigned by HMBC analysis), 141.8, 137.5, 132.1, 131.5, 128.8, 128.8, 128.6, 128.5, 127.5, 126.8, 126.3, 123.7 (assigned by HMBC analysis), 123.4, 123.2, 122.1, 120.5, 119.5, 119.0, 54.3, 48.9, 44.6, 35.1, 31.6. IR: (n max/cm-1) 3202, 2956, 2851, 2347, 1740, 1689, 1591, 1313, 1201, 1012. HRMS (EI) m/z = 690.3436 $[M+H]^+$ (calc. for $C_{44}H_{44}N_5O_3$ 690.3439). UV: λ_{max}(MeCN)/nm (ε / $mol^{-1}cm^{-1}dm^3$) 361 (14285).

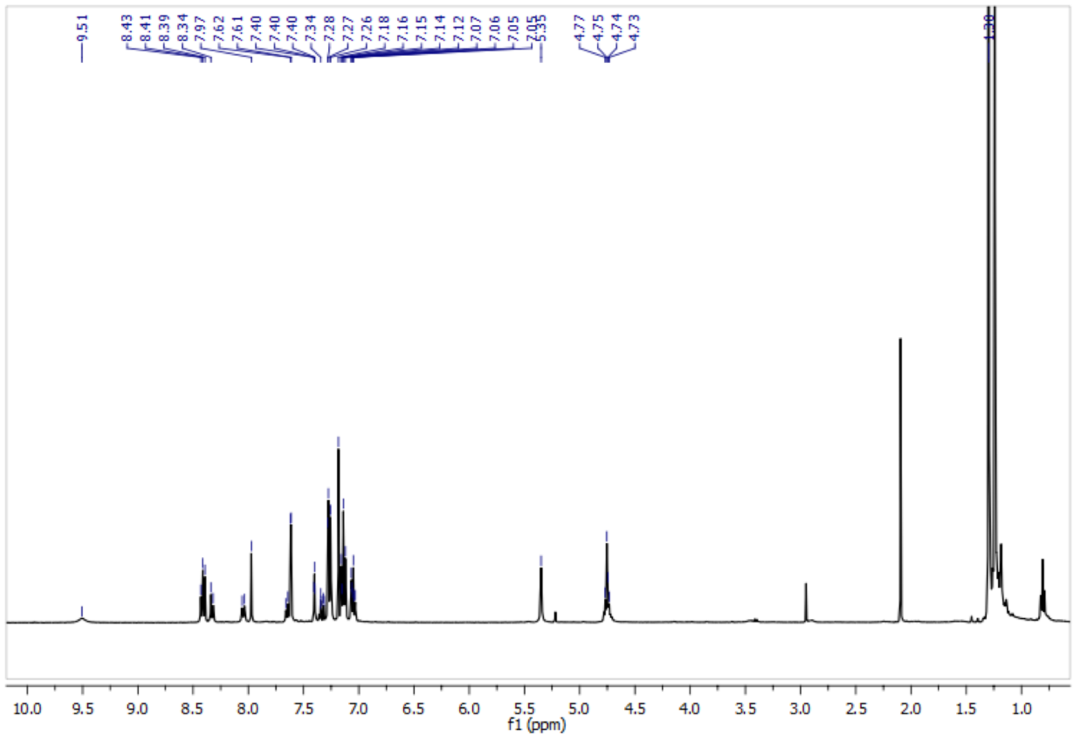

Figure S25 ^{1}H NMR ($CDCl_3$, 400 MHz, 298 K) of **S11**.

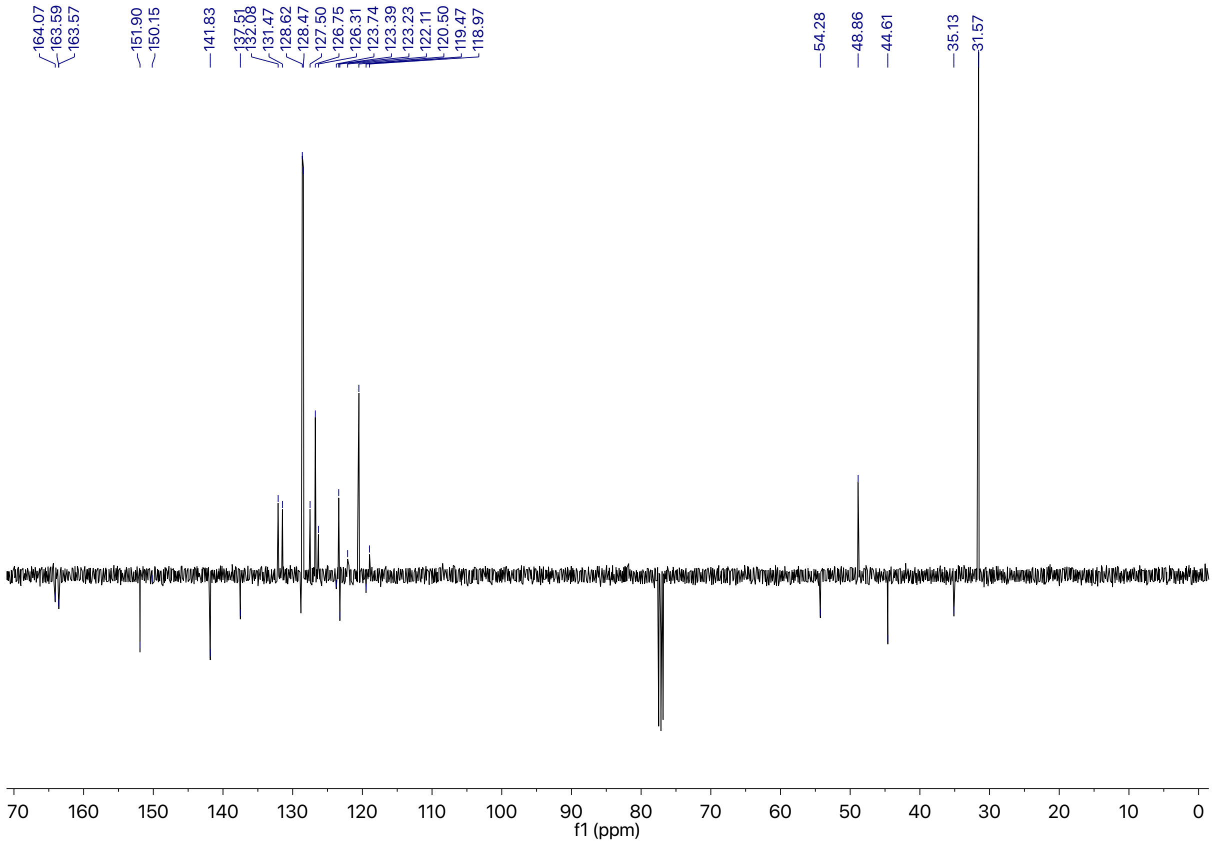

Figure S26 JMOD NMR ($CDCl_3$, 101 MHz, 298 K) of **S11**.

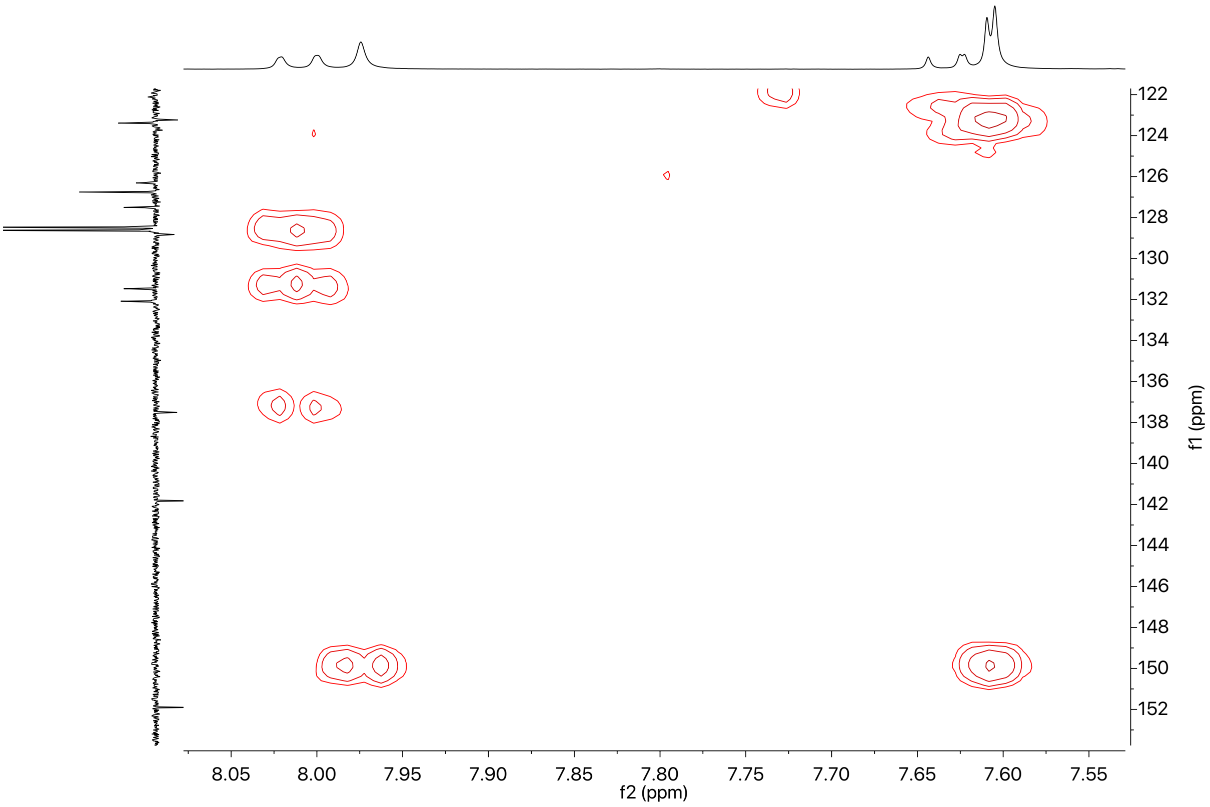

Figure S27 Partial HMBC NMR ($CDCl_3$, 400 MHz, 298 K) of **S11**.

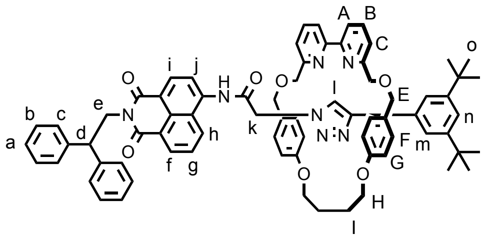

Rotaxane 6

General procedure was employed with macrocycle **1** (0.20 g, 0.41 mmol) acetylene **2** (0.09 g, 0.41 mmol), azide **S10** (0.20 g, 0.41 mmol), $[Cu(MeCN)_4]PF_6$ (0.15 g, 0.40 mmol), and DIPEA (72 µL, 0.41 mmol) in EtOH (20 mL). Purification as noted gave rotaxane **6** as a yellow residue (0.29 g, 60%). ^{1}H NMR ($CDCl_3$, 400 MHz, 298 K) δ 10.59 (s, 1H, -*NH*-), 8.59 (dd, *J* = 8.6, 0.8, 1H, H_f), 8.46 (d, *J* = 8.3, 1H, H_i), 8.35 (dd, *J* = 7.3, 0.8, 1H, H_h), 7.86 (d, *J* = 8.4, 1H, H_j), 7.81-7.77 (m, 4H, H_m and H_B), 7.65 (d, *J* = 7.7, 2H, two of H_A/H_C), 7.58 (s, 1H, H_l), 7.52 (d, *J* = 7.7, 2H, two of H_A/H_C), 7.46 (t, *J* = 1.8, 1H, H_n), 7.43-7.40 (m, 4H, H_c), 7.29-7.24 (m, 4H, H_b), 7.18-7.14 (m, 2H, H_a), 7.09 (dd, *J* = 8.5, 7.3, 1H, H_g), 6.85 (d, *J* = 8.6, 4H, H_F), 6.54 (d, *J* = 8.6, 4H, H_G), 4.92-4.84 (m, 5H, H_d, H_e and H_k), 4.59 (d, *J* = 11.8, 2H, two of H_E), 4.33 (d, *J* = 11.8, 2H, two of H_E), 4.31 (d, *J* = 12.8, 2H, two of H_D), 4.07 (d, *J* = 12.8, 2H, two of H_D), 4.06-4.03 (m, 4H, H_H), 2.11-1.93 (m, 4H, H_I), 1.44 (s, 18H, H_o) ^{13}C NMR ($CDCl_3$, 101 MHz, 298 K) δ 164.5, 163.9, 163.1, 159.4, 159.2, 155.3, 151.4, 148.3, 141.9, 141.7, 139.7, 138.1, 132.4, 130.7, 130.3, 129.8, 128.6, 128.5, 128.3, 128.1, 126.5, 125.1, 122.8, 122.5, 122.2, 122.1, 121.7, 121.4, 120.1, 117.4, 117.0, 115.2,

73.1, 70.3, 66.4, 52.3, 48.9, 44.3, 35.0, 31.5, 31.3, 24.6. IR: (ν_{max}/cm^{-1}) 3207, 2966, 2857, 2339, 1730, 1696, 1588, 1306, 1212, 1093, 983. HRMS (EI) m/z = 1172.5648 [M+H]$^+$ (calc. for $C_{74}H_{73}N_7O_7$ 1172.5644). UV: λ_{max}(MeCN)/nm (ε / mol^{-1}cm^{-1}dm^3) 380 (11905).

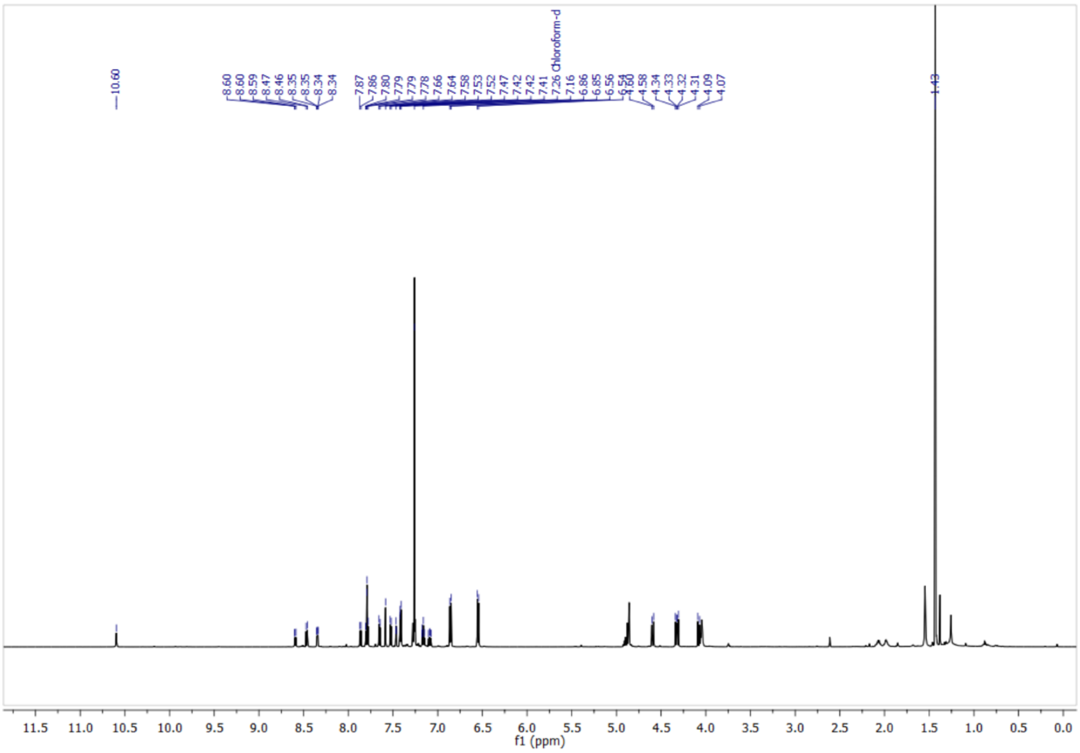

Figure S28 ^{1}H NMR ($CDCl_3$, 400 MHz, 298 K) of **6**.

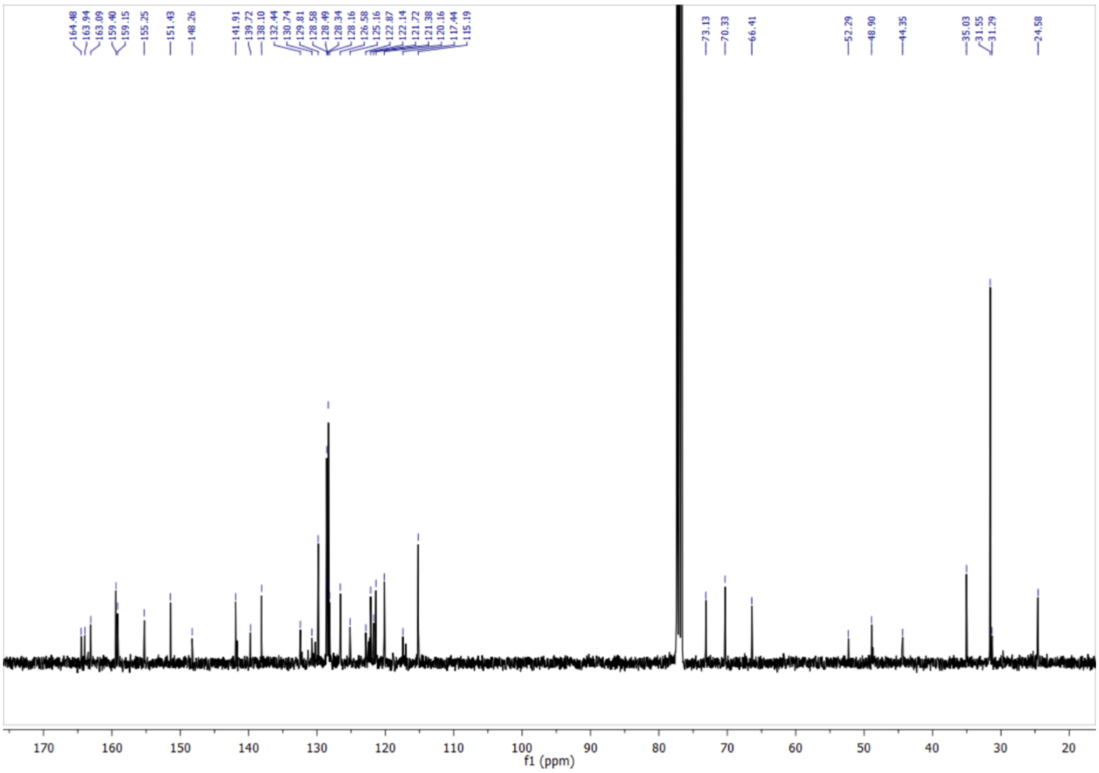

Figure S29 ^{13}C NMR ($CDCl_3$, 101 MHz, 298 K) of 6.

6. Synthesis of Rotaxane 7

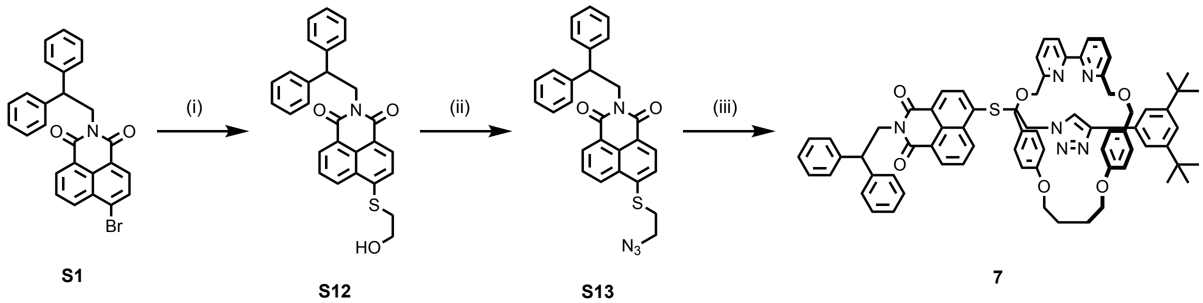

Scheme S4 Synthesis of rotaxane **7**. Conditions: (i) 2-thiolethanol, Cu_2O, K_2CO_3, DMSO, 90 °C, 16 h, 82%. (ii) diphenyl phosphoryl azide, DEAD, PPh_3, THF, r.t., 2 h, 72%. (iii) **1**, **2**, $[Cu(MeCN)_4]PF_6$, $^iPr_2EtN$, EtOH, r.t., 16 h, 86%.

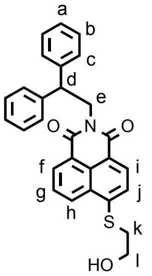

2-(2,2-diphenylethyl)-6-((2-hydroxyethyl)thio)-1H-benzo[de]isoquinoline-1,3(2H)-dione S12

To a solution of bromide **S1** (0.3 g, 0.64 mmol) in DMSO (10 mL) was added Cu_2O (1.8 mg, 0.013 mmol), K_2CO_3 (18.0 mg, 0.13 mmol) and 2-thioethanol (0.46 mL, 6.4 mmol), and was heated at 90 °C overnight. The reaction mixture was diluted with brine (40 mL), and the aqueous portion extracted with EtOAc (2 x 40 mL). The organic phase was dried with $MgSO_4$, filtered and concentrated *in vacuo*. The resulting oil was purified by flash column chromatography (CH_2Cl_2 to 8:2 CH_2Cl_2/EtOAc) to give alcohol **S12** as a yellow foam (0.24 g, 82%). ^1H NMR ($CDCl_3$, 400 MHz, 298 K) δ 8.52-8.47 (m, 2H, H_f and H_h), 8.34 (d, *J* = 7.8, 1H, H_i), 7.67 (dd, *J* = 8.3, 7.5, 1H, H_g), 7.56 (d, *J* = 7.9, 1H, H_j), 7.37-7.35 (m, 4H, H_c), 7.26-7.21 (m, 4H, H_b) 7.16-7.12 (m, 2H, H_a), 4.84-4.83 (m, 3H, H_d and H_e), 3.96-3.93 (m, 2H, H_l), 3.35 (t, *J* = 6.2, 2H, H_k), 2.21 (br s, 1H, -OH). ^{13}C NMR ($CDCl_3$, 101 MHz, 298 K) δ 164.0, 164.0, 143.6, 141.8, 131.7, 130.8, 130.2, 129.9, 128.6, 128.5, 128.4, 126.9, 126.7, 123.9, 123.1, 119.7, 60.5, 48.9, 44.6, 35.6. IR: (ν_{max}/cm^{-1}) 3505, 2940, 2346, 1695, 1640, 1559, 1352, 1245, 973. M.p. (°C) 39-41. HRMS (ESI+) m/z = 454.1462 $[M+H]^+$ (calc. for $C_{28}H_{24}NO_3S$ 491.1133). UV: λ_{max}(MeCN)/nm (ε / $mol^{-1}cm^{-1}dm^3$) 389 (5331).

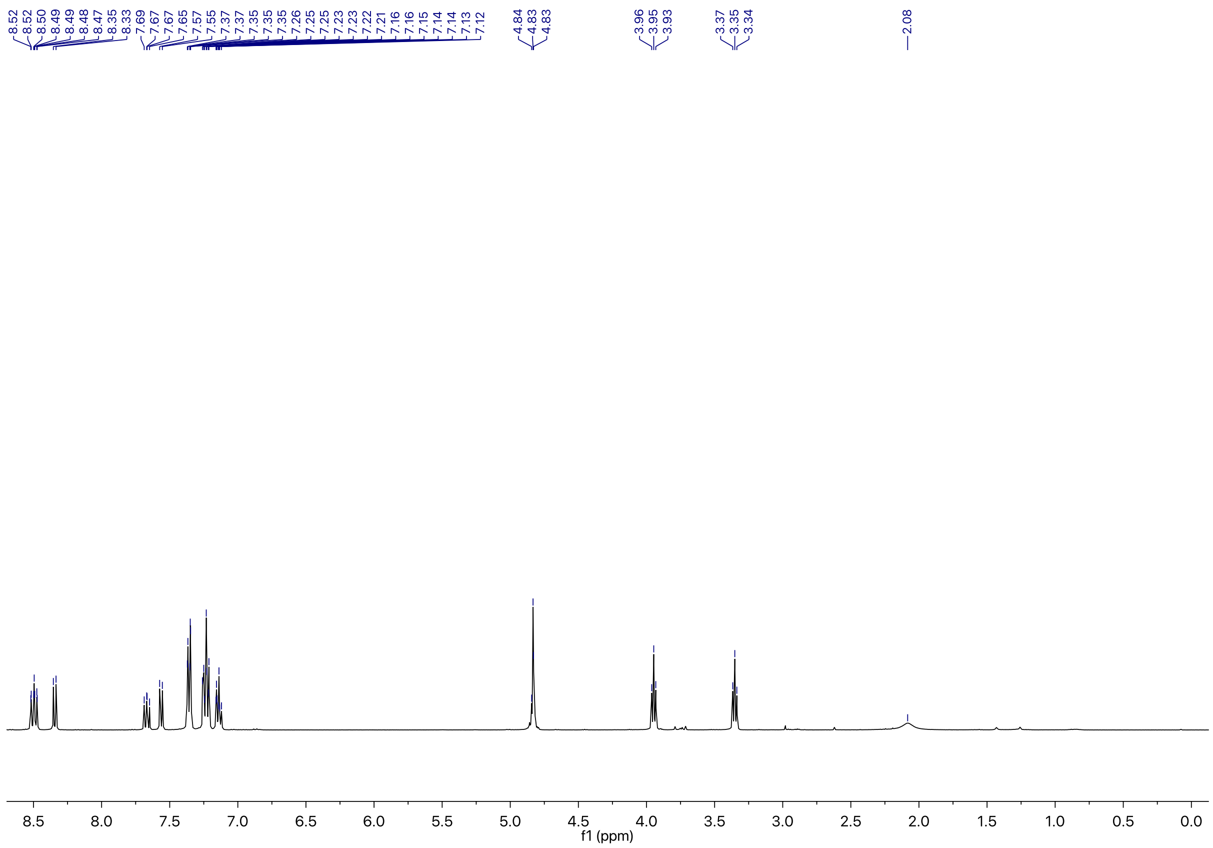

Figure S30 ^{1}H NMR ($CDCl_3$, 400 MHz, 298 K) of **S12**.

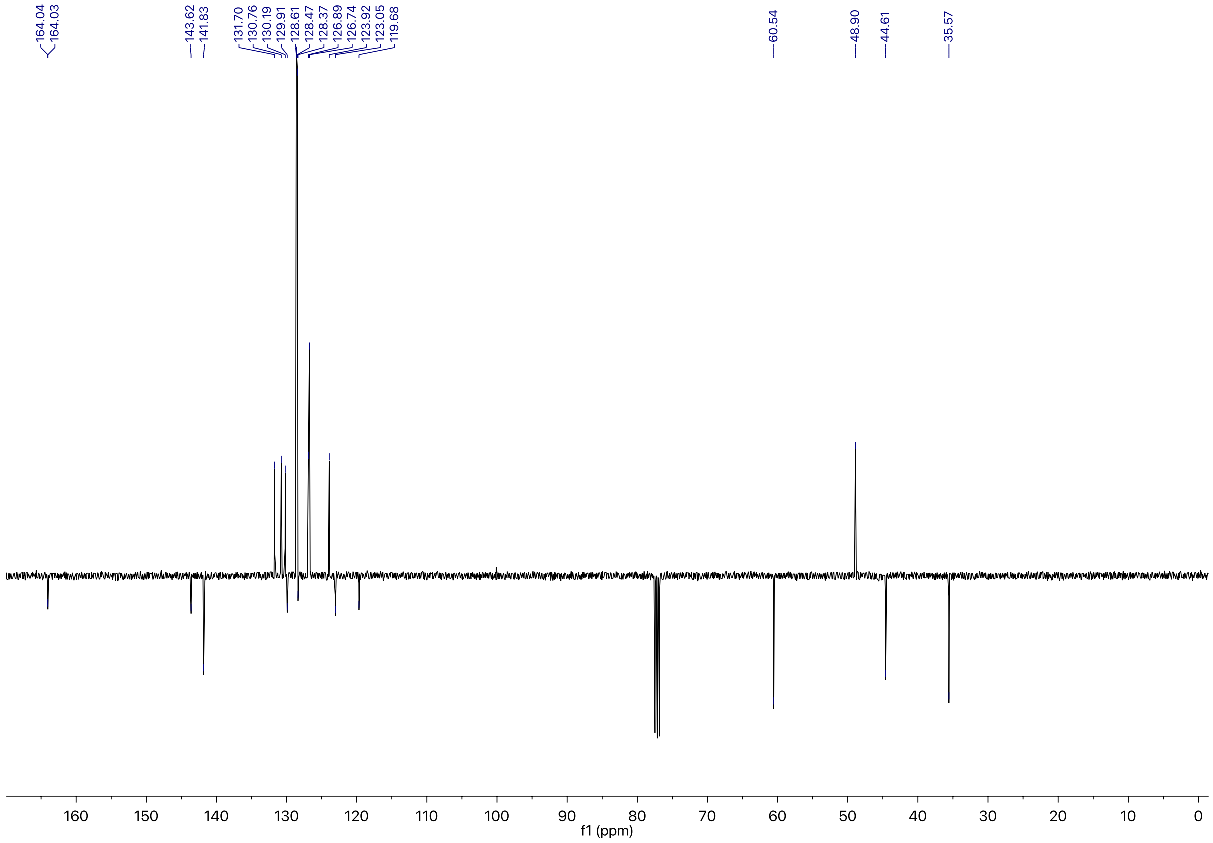

Figure S31 JMOD NMR ($CDCl_3$, 101 MHz, 298 K) of **S12**.

6-((2-azidoethyl)thio)-2-(2,2-diphenylethyl)-1H-benzo[de]isoquinoline-1,3(2H)-dione (S13)

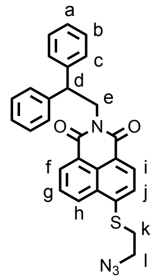

Alcohol **S12** (0.13 g, 0.28 mmol, 1 equiv.) and triphenylphosphine (0.22 g, 0.83 mmol, 3 equiv.) were combined in a sealed flask which was purged with N_2. THF (12 mL) was added, and the stirring solution was cooled to 0 °C. DEAD (0.13 mL, 0.83 mmol, 3 equiv.) was added to the reaction, followed by diphenyl phosphoryl azide (0.19 mL, 0.83 mmol, 3 equiv.), and stirred for 15 min. The reaction was then stirred at r.t. for 2 h, and then concentrated *in vacuo*. The resulting oil was purified by flash column chromatography, Petrol/ CH_2Cl_2 1:1 to CH_2Cl_2 to CH_2Cl_2/EtOAc 8:2 to give azide **S13** as an orange foam (0.095 g, 72%). ^{1}H NMR ($CDCl_3$, 400 MHz, 298 K) δ 8.57-8.49 (m, 2H, H_f and H_h), 8.39 (d, J = 7.8, 1H, H_i), 7.71 (dd, J = 7.5, 8.2, 1H, H_g), 7.57 (d, J = 7.8, 1H, H_j), 7.40-7.31 (m, 4H, H_c), 7.27-7.18 (m, 4H, H_b), 7.16-7.10 (m, 2H, H_a), 4.85-4.80 (m, 3H, H_d and H_e), 3.58 (t, J = 6.7, 2H, H_l), 3.30 (t, J = 6.7, 2H, H_k).^{13}C NMR ($CDCl_3$, 101 MHz, 298 K) δ 164.2, 164.2, 142.1, 134.2, 131.4, 129.8, 128.7, 128.4, 126.6, 125.8, 125.3, 123.3, 120.6, 111.6, 104.7, 100.1, 50.1, 49.0, 44.4, 42.7. IR: (ν_{max}/cm^{-1}) 3505, 2950, 2351,

2101, 1705, 1669, 1590, 1379, 1345, 1228. M.p. (°C) 45-48. HRMS (ESI+) m/z = 501.1358 $[M+Na]^+$ (calc. for $C_{28}H_{22}N_4NaO_2S$ 501.1356). UV: λ_{max}(MeCN)/nm (ε / $mol^{-1}cm^{-1}dm^3$) 381 (6093).

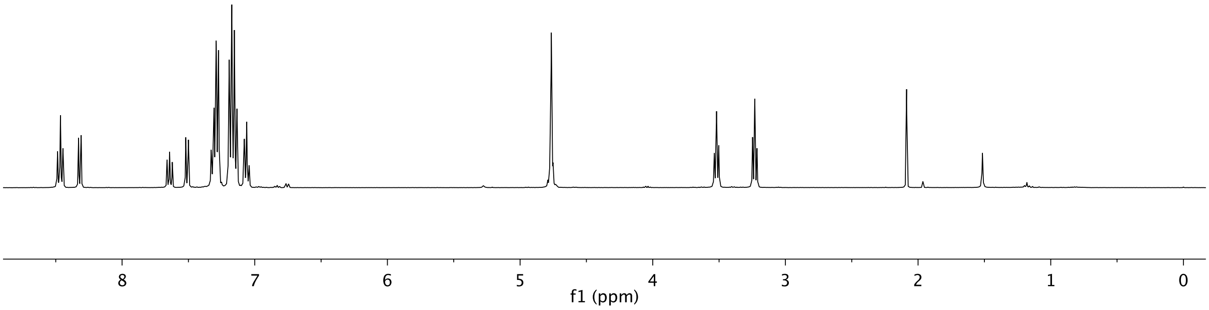

Figure S32 ^{1}H NMR ($CDCl_3$, 400 MHz, 298 K) of **S13**.

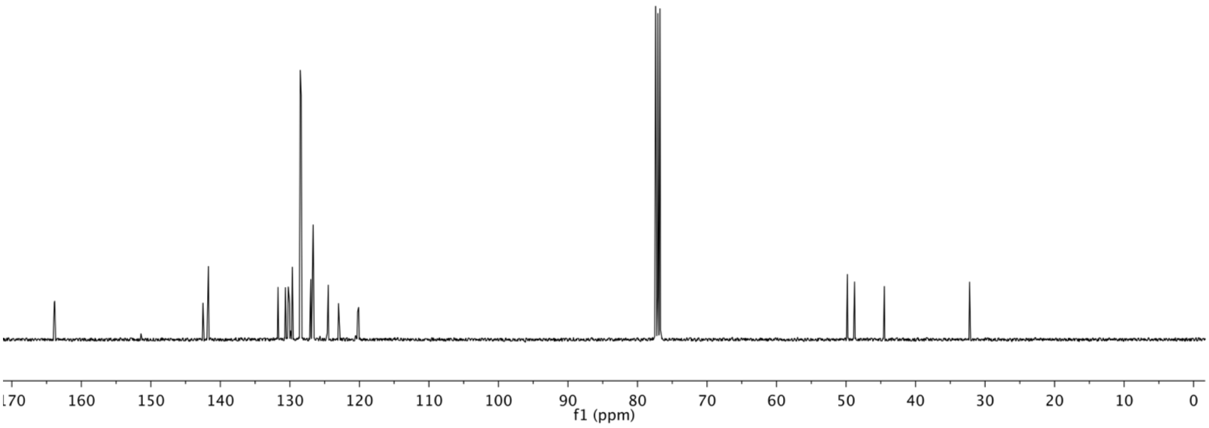

Figure S33 ^{13}C NMR ($CDCl_3$, 101 MHz, 298 K) of **S13**.

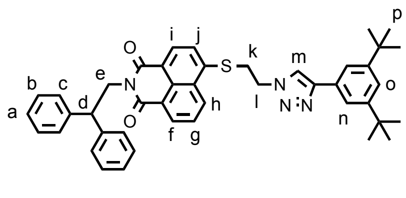

Axle S14

Azide **S13** (45 mg, 0.093 mmol), alkyne **2** (20 mg, 0.093 mmol) and $[Cu(MeCN)_4]PF_6$ (8.7 mg, 0.023 mmol) were weighed dry into a sealed flask and purged with N_2. CH_2Cl_2 (1 mL) was added, followed by DIPEA (1 drop) and the mixture stirred at r.t. for 4 h. The solvent was diluted with CH_2Cl_2 (4 mL) and washed with 16% aqueous EDTA tetrasodium-saturated ammonia solution (5 mL). The organic layer was retained and the aqueous layer extracted twice further with CH_2Cl_2. The organic extracts were combined, dried over $MgSO_4$, filtered and dried *in vacuo*. The crude mixture was purified by flash column chromatography (1:1 Petrol/CH_2Cl_2 to 10% MeCN/1:1 Petrol-CH_2Cl_2) to yield the

product **S14** as a pale yellow residue (62 mg, 96%). ^{1}H NMR ($CDCl_3$, 400 MHz, 298 K) δ 8.53 (d, *J* = 7.2, 1H, H_f), 8.51 (d, *J* = 8.5, 1H, H_h), 8.42 (d, *J* = 7.8, 1H, H_i), 7.73 (s, 1H, H_m) 7.71 (dd, *J* = 8.1, 7.3, 1H, H_g), 7.65 (d, *J* = 7.8, 2H, H_j), 7.92 (d, *J* = 1.7, 2H, H_n), 7.43 (t, *J* = 1.7, 1H, H_o), 7.39-7.31 (m, 4H, H_c), 7.26-7.20 (m, 4H, H_b), 7.17-7.13 (m, 2H, H_a), 4.86-4.82 (m, 3H, H_d and H_e), 4.69 (t, *J* = 6.7, 2H, H_l), 3.70 (t, *J* = 6.7, 2H, H_k), 1.28 (s, 18H, H_p). ^{13}C NMR ($CDCl_3$, 101 MHz, 298 K) δ 163.9, 163.8, 151.6, 149.0 (*via* HMBC analysis), 141.8, 141.5, 131.9, 130.8, 130.4, 130.3, 129.5, 128.6, 128.5 (overlap with 1C_q *via* HMBC analysis), 127.3, 126.8, 125.3, 123.3, 122.8, 120.8, 120.3, 120.2, 49.0, 48.9, 44.6, 35.1, 33.1, 31.6. IR: (ν_{max}/cm^{-1}) 2970, 2901, 2839, 2353, 1711, 1656, 1569, 1202, 1186, 1112, 1093. HRMS (EI) m/z = 693.3255 $[M+H]^+$ (calc. for $C_{44}H_{45}N_4O_2S$ 693.3258). UV: λ_{max}(MeCN)/nm (ε / mol^{-1}cm^{-1}dm^3) 378 (12228).

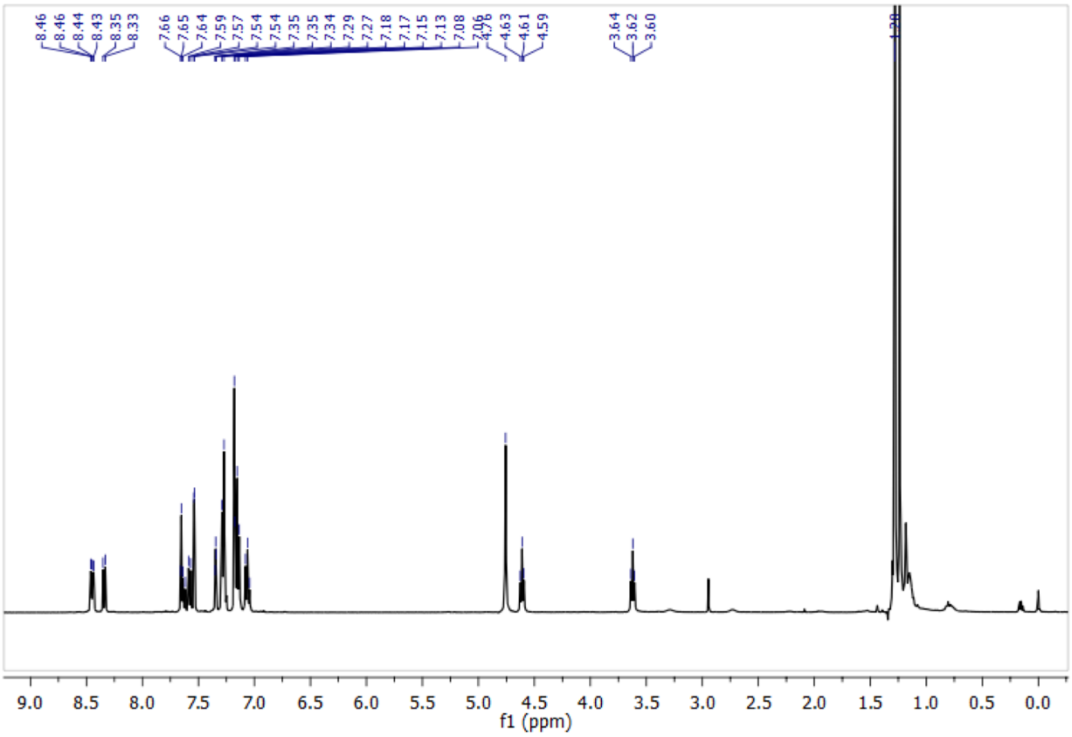

Figure S34 ^{1}H NMR ($CDCl_3$, 400 MHz, 298 K) of **S14**.

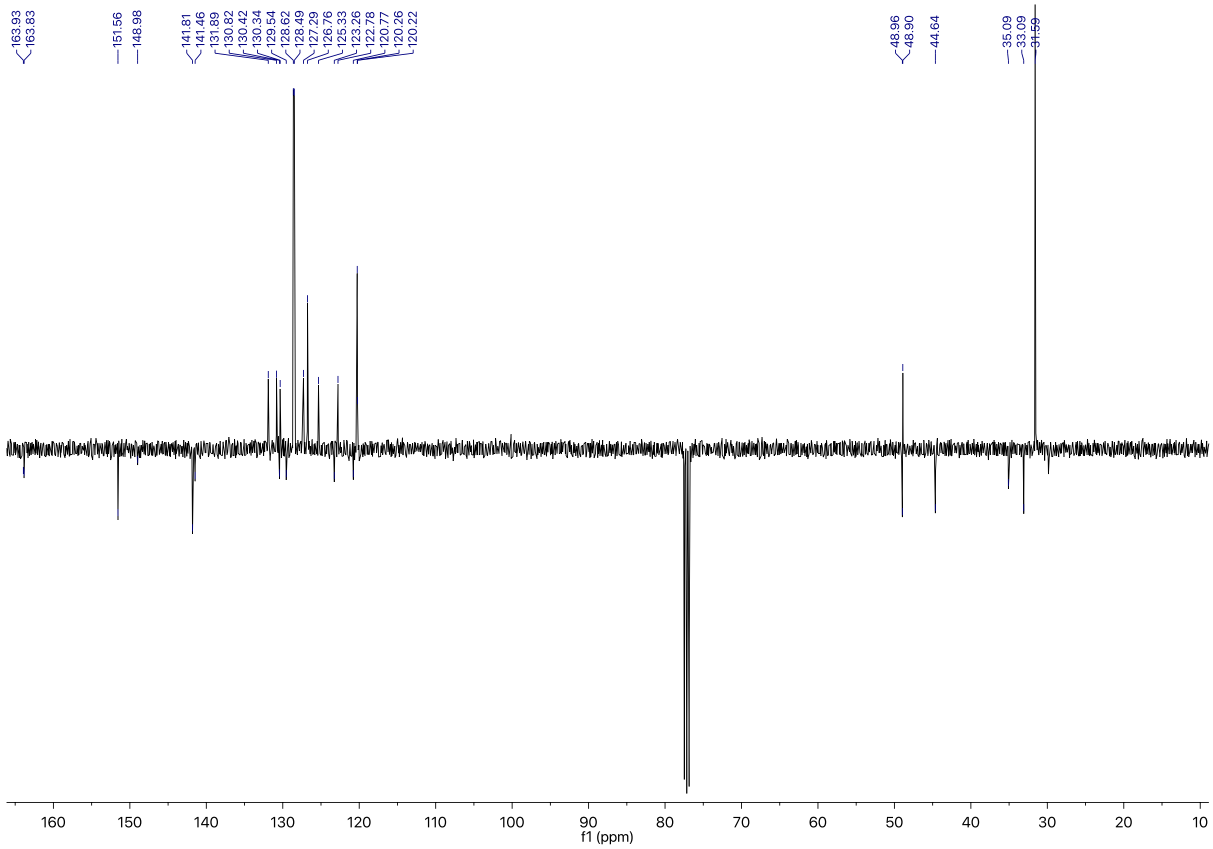

Figure S35 JMOD NMR ($CDCl_3$, 101 MHz, 298 K) of **S14**.

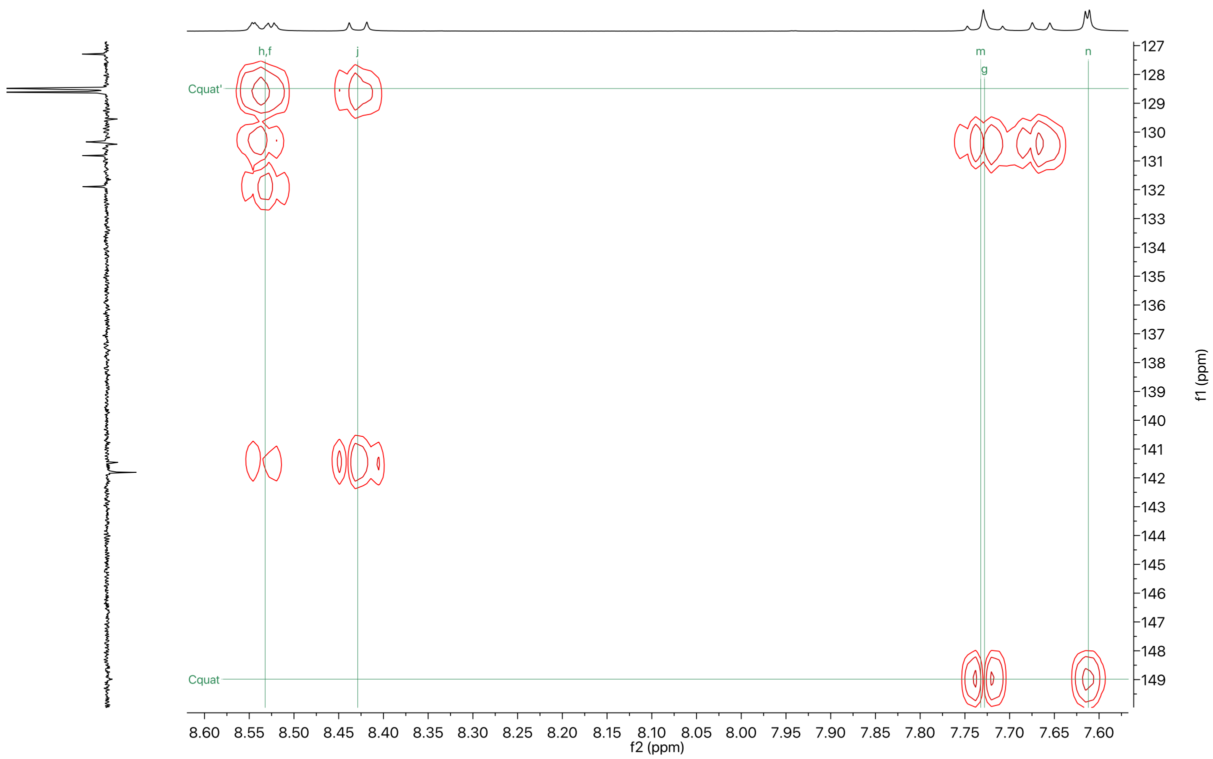

Figure 36 Partial HMBC NMR ($CDCl_3$, 400 MHz, 298 K) of **S14**.

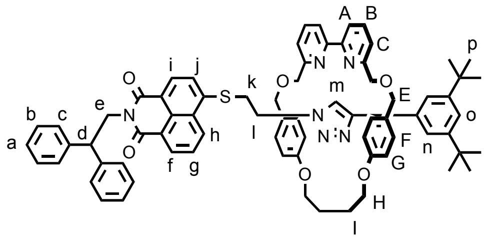

Rotaxane 7

General procedure was employed with macrocycle **1** (0.25 g, 0.52 mmol) acetylene **2** (0.11 g, 0.52 mmol), azide **S13** (0.25 g, 0. 52 mmol), $[Cu(MeCN)_4]PF_6$ (0.18 g, 0.50 mmol), and DIPEA (90 µL, 0.52 mmol) in EtOH (25 mL). Purification as noted gave rotaxane **7** as a pale yellow foam (0.52 g, 86%). ^{1}H NMR ($CDCl_3$, 400 MHz, 298 K) δ 9.41 (s, 1H, H_m), 8.43 (d, *J* = 7.2, 1H, H_f), 8.33 (d, *J* = 8.3, 1H, H_h), 7.76 (d, *J* = 7.8, 1H, H_i), 7.67-7.61 (m, 3H, H_g and H_B), 7.61-7.59 (m, 2H, two of H_C/H_A), 7.47-7.42 (m, 4H, H_n and two of H_C/H_A), 7.41-7.37 (m, 4H, H_c), 7.27 (t, *J* = 1.6, 1H, H_o), 7.27-7.22 (m, 4H, H_b), 7.17-7.11 (m, 2H, H_a), 6.90 (d, *J* = 7.8, 1H, H_j), 6.82 (d, *J* = 8.5, 4H, H_F), 6.71 (d, *J* = 8.5, 4H, H_G), 4.87-4.83 (m, 3H, H_d and H_e), 4.62 (d, *J* = 11.8, 2H, two of H_E), 4.58-4.50 (m, 2H, two of H_H), 4.21 (d, *J* = 11.8, 2H, two of H_E), 4.14-4.07 (m, 2H, two of H_H), 4.09 (d, *J* = 12.2, 2H, two of H_D), 4.01 (d, *J* = 12.2, 2H, two of H_D), 3.18-3.12 (m, 2H, H_l), 2.38-2.23 (m, 2H, H_k), 2.30-2.19 (m, 2H, two of H_I), 2.06-1.94 (m, 2H, two of H_I), 1.85 (s, 18H, H_p). ^{13}C NMR ($CDCl_3$, 101 MHz, 298 K) δ 164.0, 163.8, 159.0, 158.7, 155.6, 151.1, 147.5, 142.7, 141.7, 137.4, 131.3, 130.6, 130.5, 129.7, 129.5, 128.8, 128.5, 128.4, 127.9, 126.6, 126.5, 125.3, 125.3, 125.2, 122.8, 122.6, 121.7, 121.6, 120.7, 120.1, 120.0, 120.0, 119.8, 119.2, 114.9, 72.9, 70.8, 66.4, 48.8, 47.5, 44.4, 34.8, 31.5, 31.3, 29.4, 24.7. IR: (ν_{max}/cm^{-1}) 2970, 2901, 2839, 2353, 1711, 1656, 1569, 1202, 1186, 1112, 1093. M.p. (°C) 50-52. HRMS (EI) m/z = 1175.5479 $[M+H]^+$ (calc. for $C_{74}H_{74}N_6O_6S$ 1175.5463). UV: λ_{max}(MeCN)/nm (ε / mol^{-1}cm^{-1}dm^3) 381 (10674).

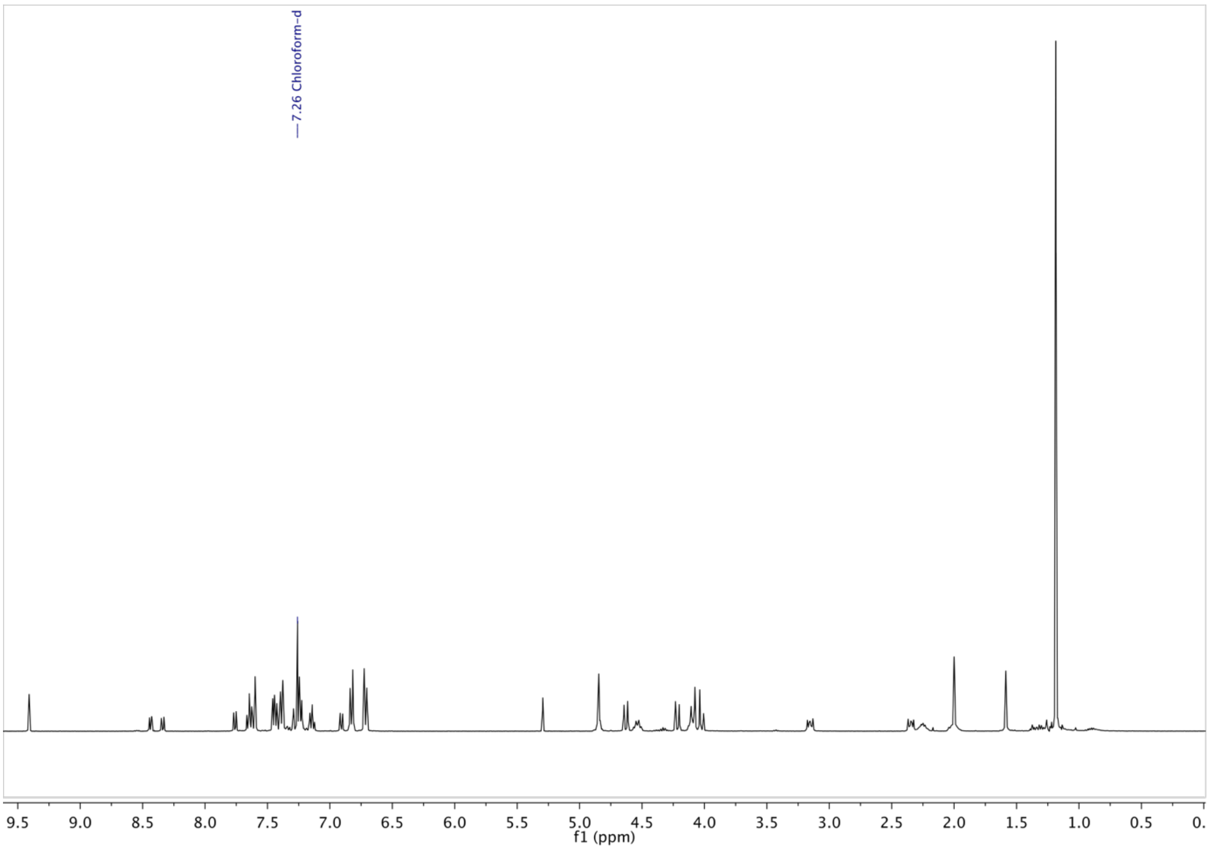

Figure S37 ^{1}H NMR ($CDCl_3$, 400 MHz, 298 K) of **7**.

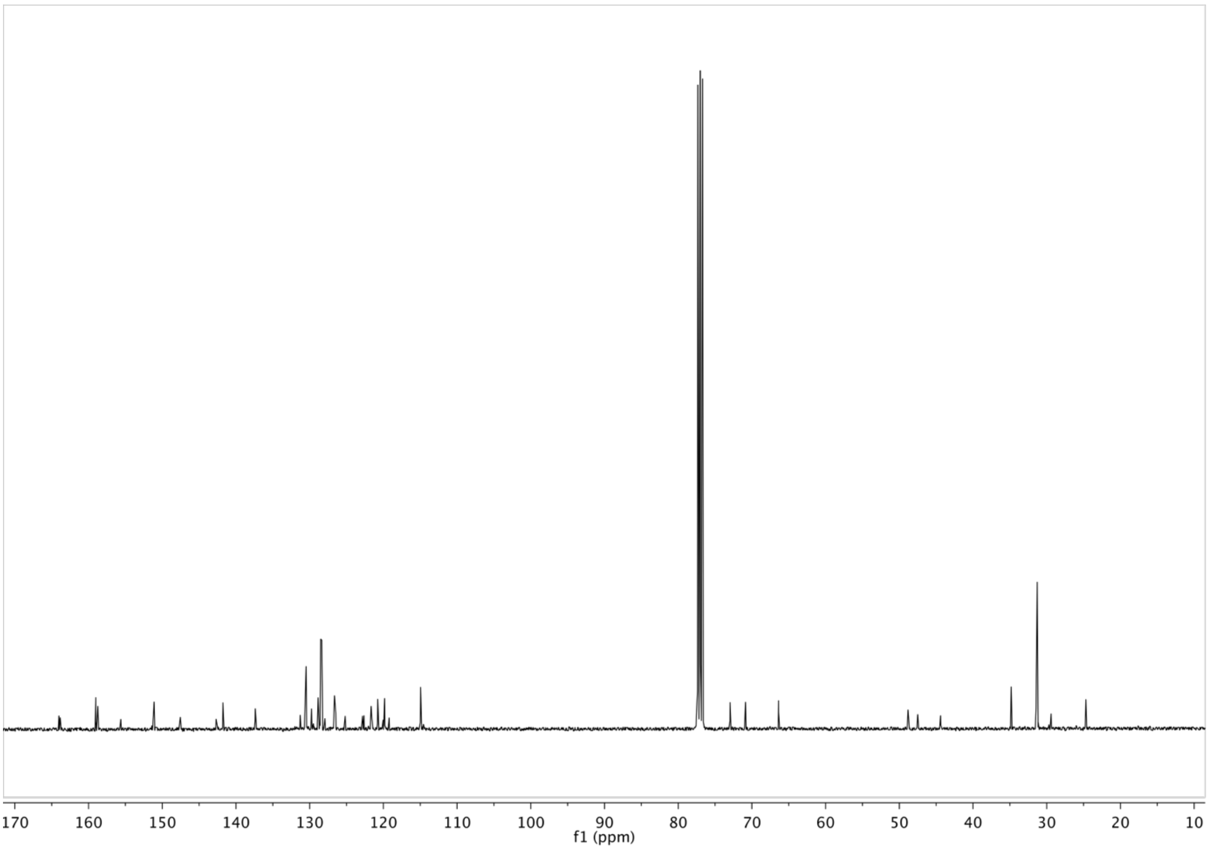

Figure S38 ^{13}C NMR ($CDCl_3$, 101 MHz, 298 K) of **7**.

7. NMR stack plots

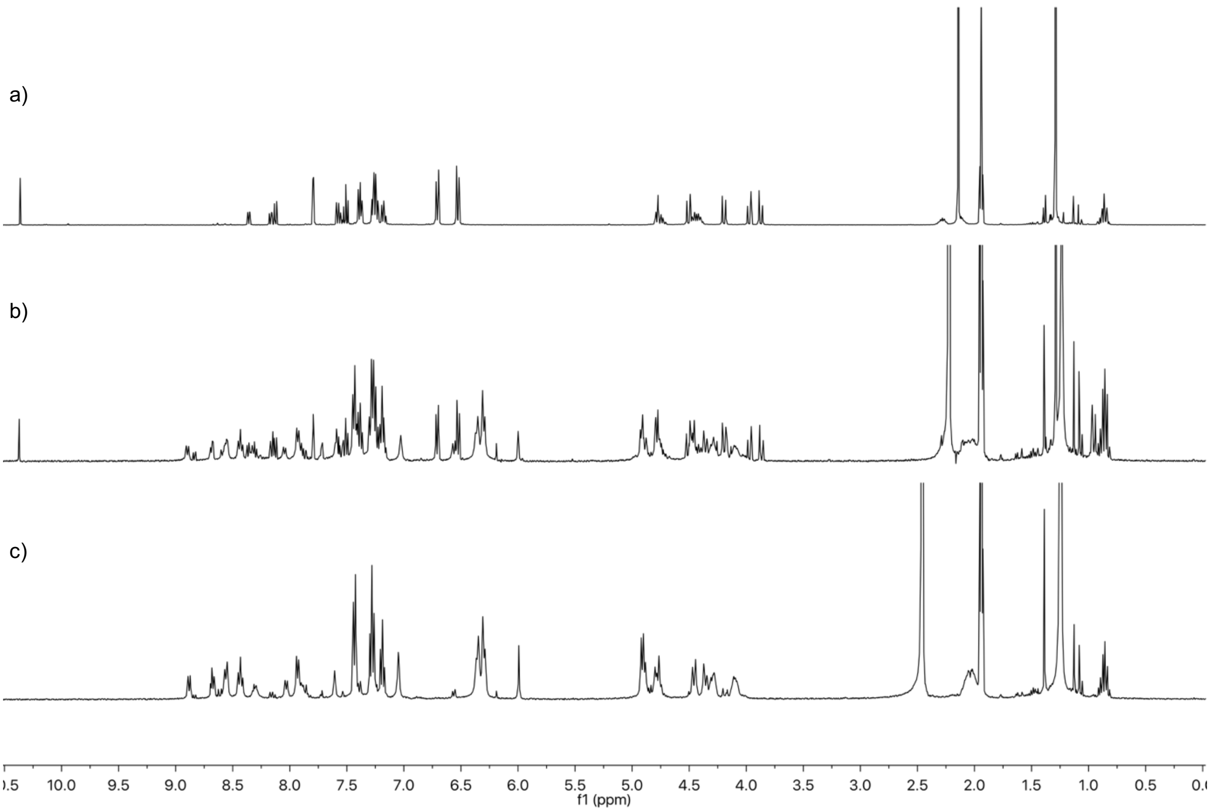

Figure S39 ^{1}H NMR (CD_3CN, 400 MHz, 298 K) of (a) **4**, (b) **4** + $ZnClO_4.6H_2O$ (0.5 equiv.), (c) **4** + $ZnClO_4.6H_2O$ (1 equiv.).

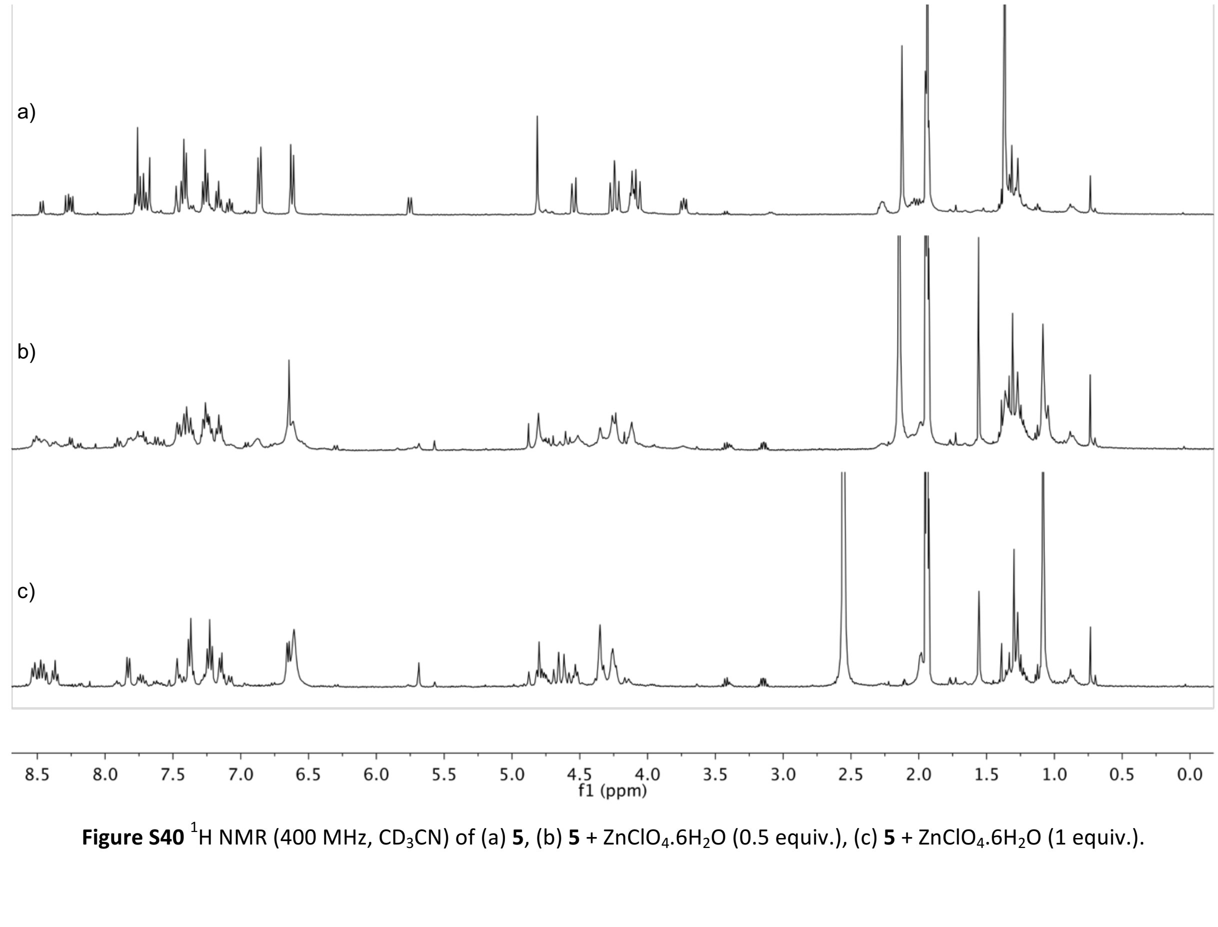

Figure S40 ^{1}H NMR (400 MHz, CD_3CN) of (a) **5**, (b) **5** + $ZnClO_4.6H_2O$ (0.5 equiv.), (c) **5** + $ZnClO_4.6H_2O$ (1 equiv.).

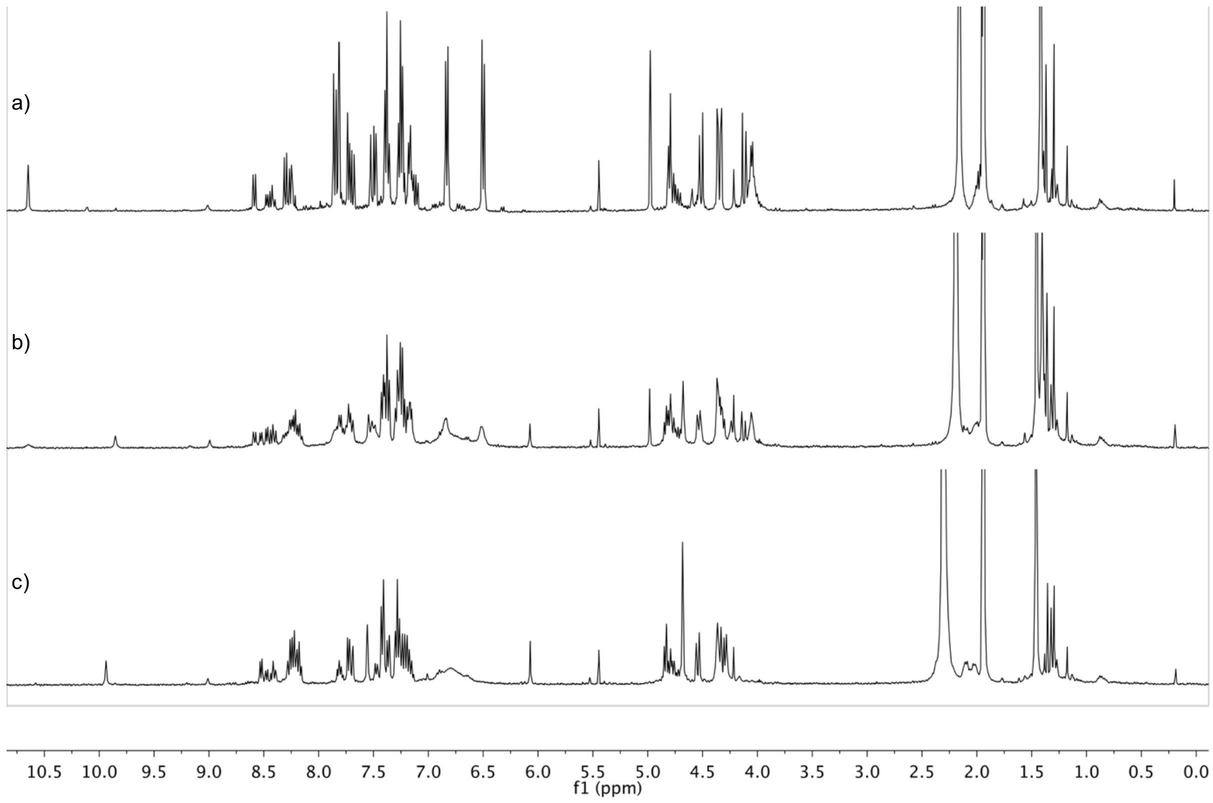

Figure S41 ^{1}H NMR (CD_3CN, 400 MHz, 298 K) of (a) **6**, (b) **6** + $ZnClO_4.6H_2O$ (0.5 equiv.), (c) **6** + $ZnClO_4.6H_2O$ (1 equiv.).

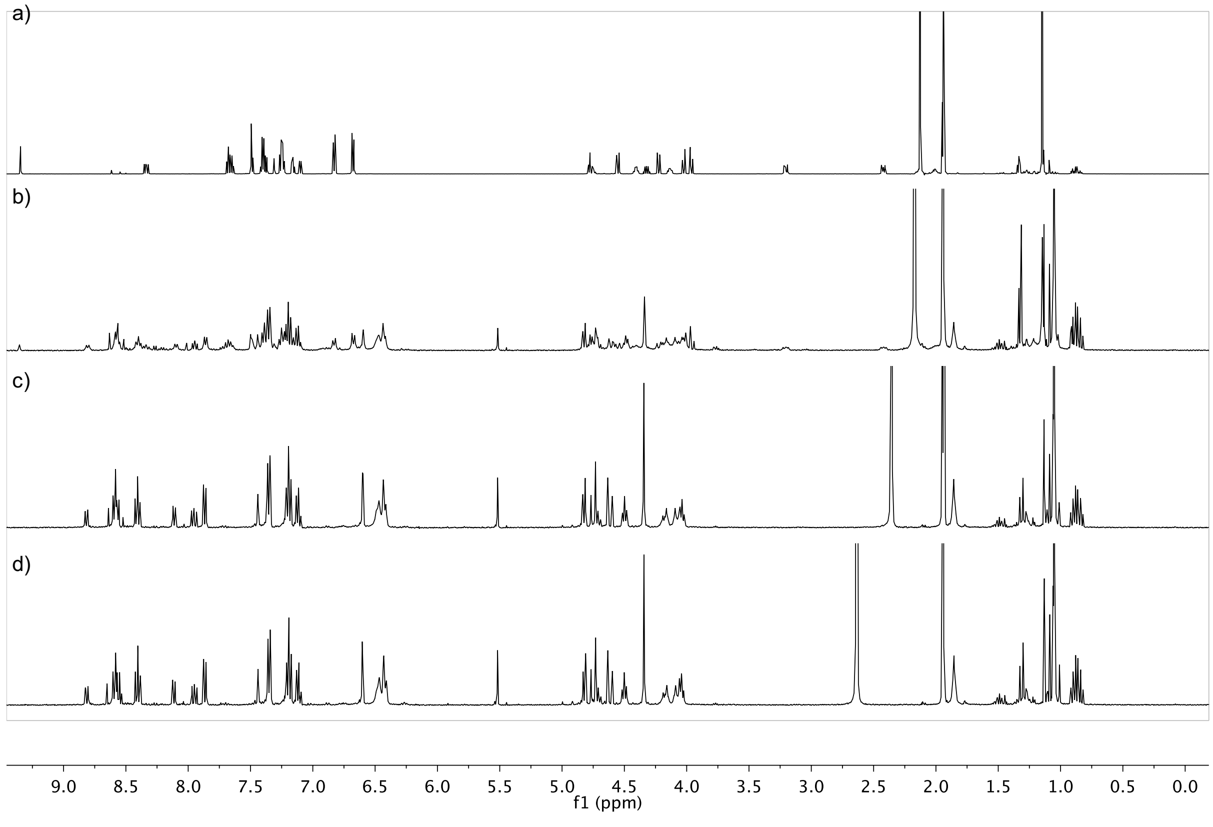

Figure S42 ^{1}H NMR (CD_3CN, 400 MHz, 298 K) of (a) **7**, (b) **7** + $ZnClO_4.6H_2O$ (0.5 equiv.), (c) **7** + $ZnClO_4.6H_2O$ (1 equiv.) and (d) **7** + $ZnClO_4.6H_2O$ (2 equiv.).

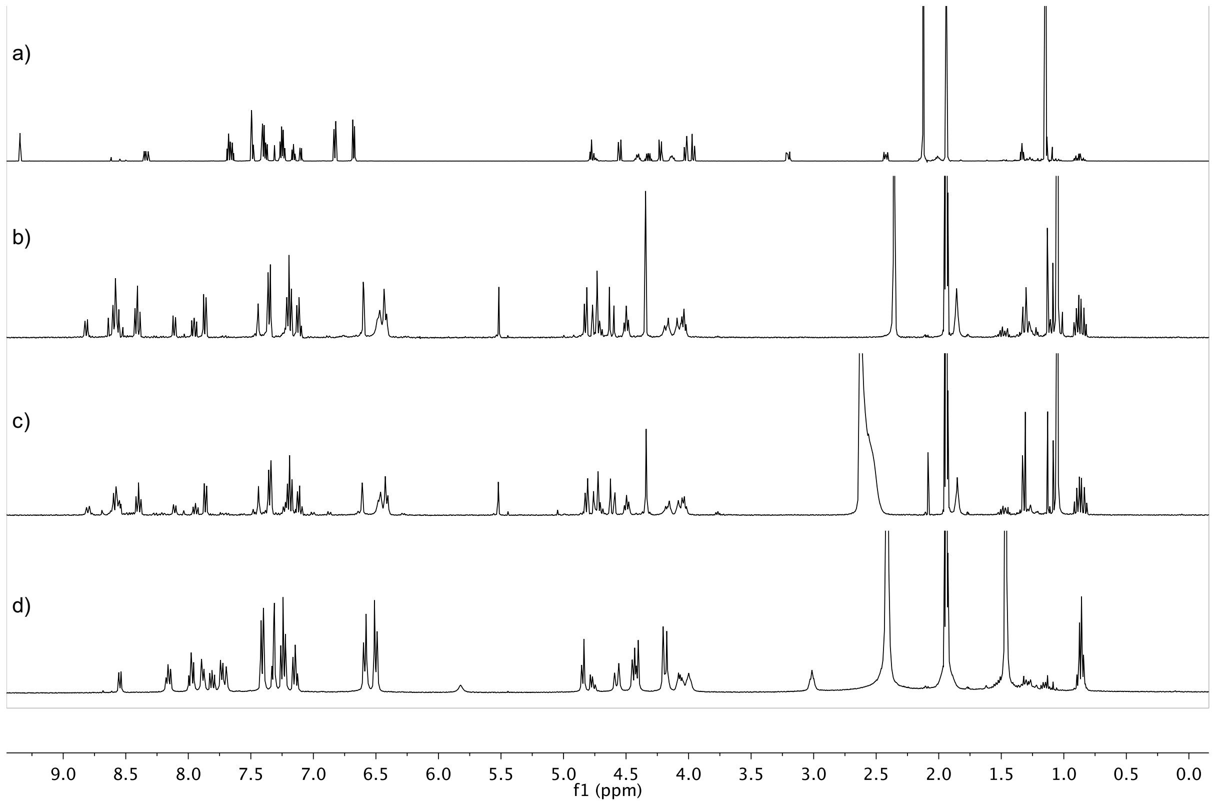

Figure S43 ^{1}H NMR (CD_3CN, 400 MHz, 298 K) of (a) **7**, (b) $[Zn(\mathbf{7})]^{2+}$, (c) **7** + $Zn(ClO_4)_2.6H_2O$ (1 equiv.) + $Cd(ClO_4)_2.6H_2O$ (2 equiv.) and (d) $[Cd(\mathbf{7})]^{2+}$.

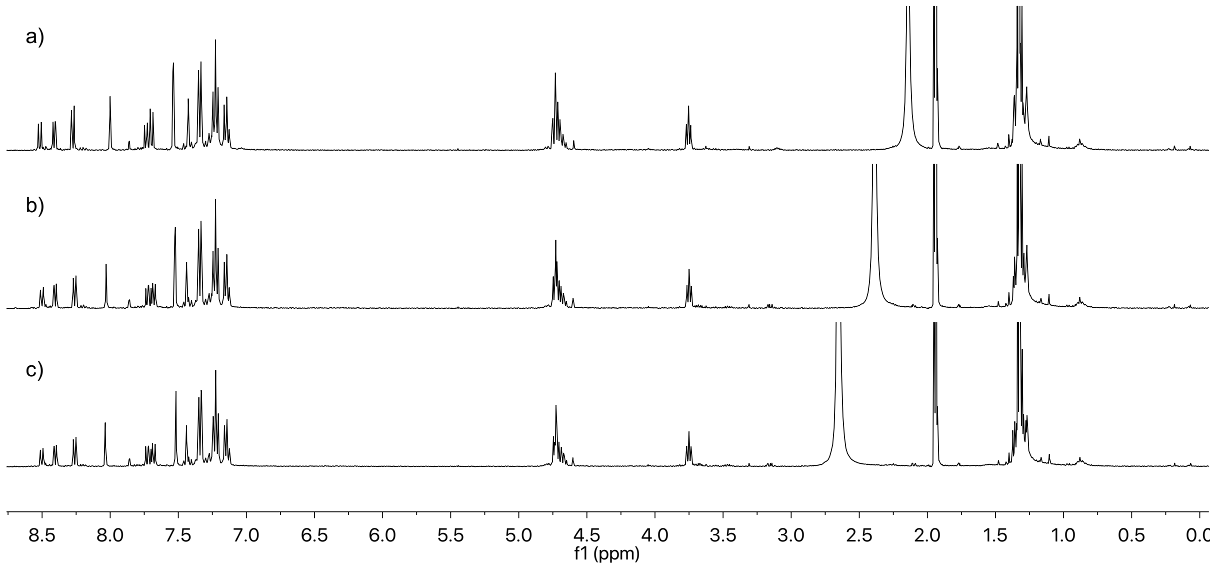

Figure S44 ^{1}H NMR (CD_3CN, 400 MHz, 298 K) of (a) **S14**, (b) **S14** + $Zn(ClO_4)_2.6H_2O$ (0.5 equiv.) and (c) **S14** + $Zn(ClO_4)_2.6H_2O$ (1 equiv.)

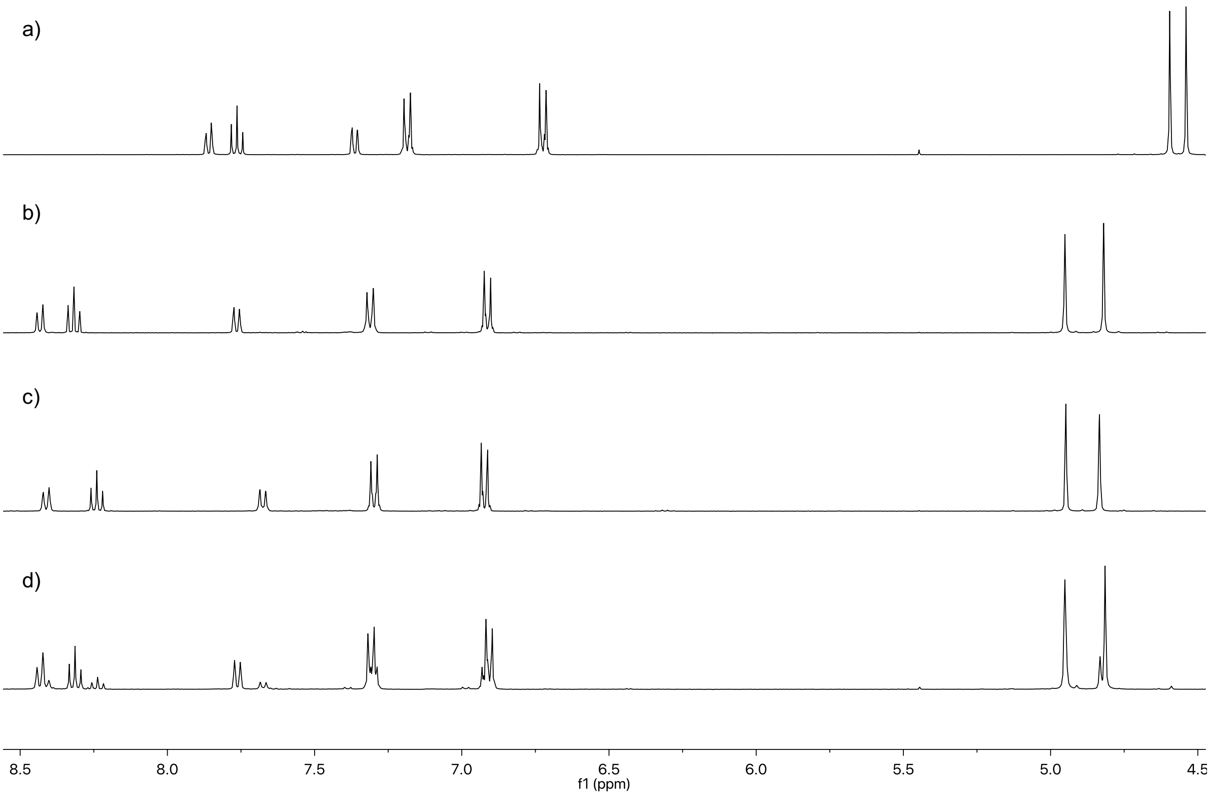

Figure S45 ^{1}H NMR (CD_3CN, 400 MHz, 298 K) of (a) **1**, (b) $[Zn(\mathbf{1})]^{2+}$, (c) $[Cd(\mathbf{1})]^{2+}$ and (d) **1** + $Zn(ClO_4)_2.6H_2O$ (1 equiv.) + $Cd(ClO_4)_2.6H_2O$ (1 equiv.).

8. UV-vis / Fluorescence titrations

Procedure: A 100 µM stock solution of the receptor was accurately prepared in the solvent of interest for the study using a volumetric flask. Solutions of metals with 100 µM of receptors to be titrated were prepared in separate 2.5 ml vials, and 1000 µL of the receptor solution were added using pipette (Eppendorf). The concentration of metal in the guest solutions was made 50 times that of the host (i.e. 5 mM). After each addition, the resulting solution was shaken vigorously and the absorbance/emission were recorded. UV-Vis data was recorded using a Varian Cary 4000 UV-Vis Spectrophotometer. Fluorescence titrations were carried out in parallel to the UV/Vis absorption measurements using a Cary Eclipse Fluorescence Spectrometer. Temperature control was provided by a Varian Cary PCB 150 Water Peltier System. The absorbance was recorded from 250 nm to 800 nm. Titrations were performed in triplicate to give K_d values. To determine association constants for the receptor-metal complexes, global analysis of the absorbance/emission data was carried out using a nonlinear least-squares curve fitting procedure with Origin 2016 software using the 1:1 global fitting model equation.

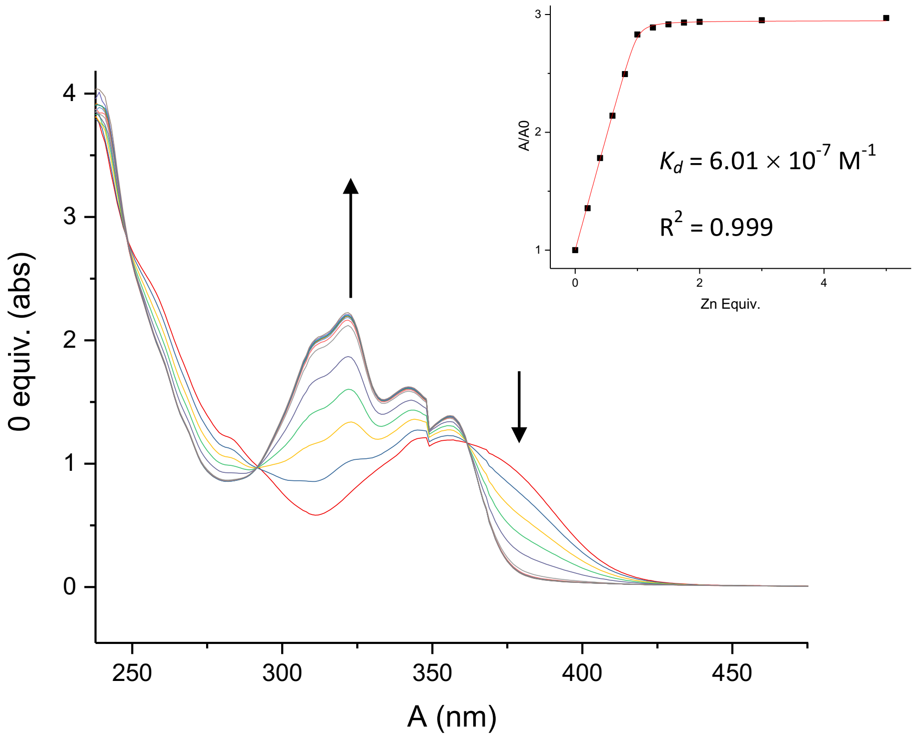

Figure S46 UV-Vis titration of **4** with $Zn(ClO_4)_2.6H_2O$ (0-5 equiv.) in MeCN, C = 100 µM. Asterisk shows the artefact from the spectrometer.

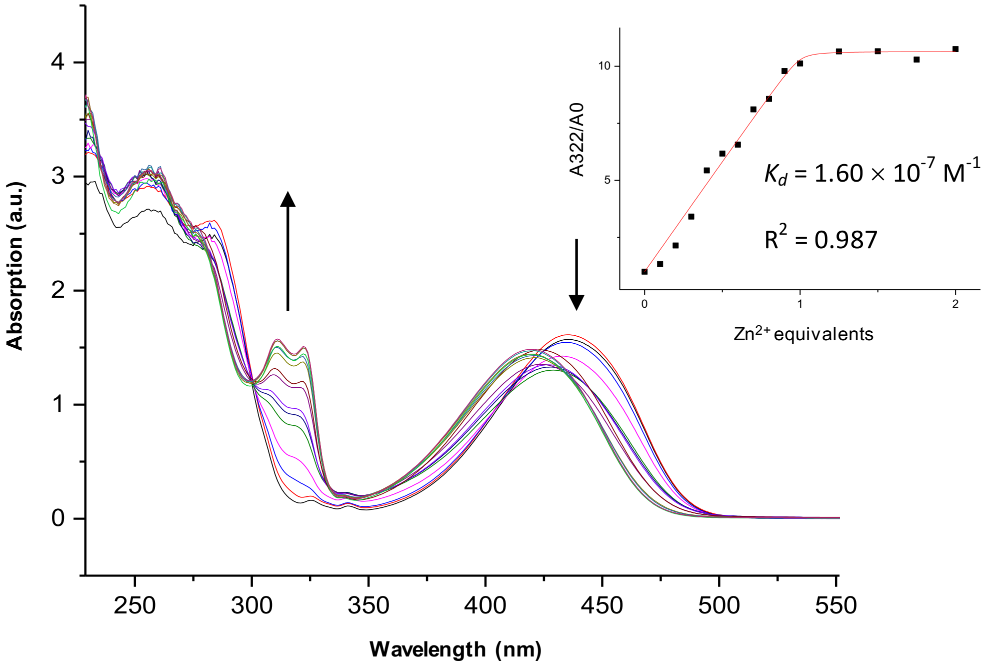

Figure S47 UV-Vis titration of **5** with $Zn(ClO_4)_2.6H_2O$ (0-2 equiv.) in MeCN, C = 100 µM.

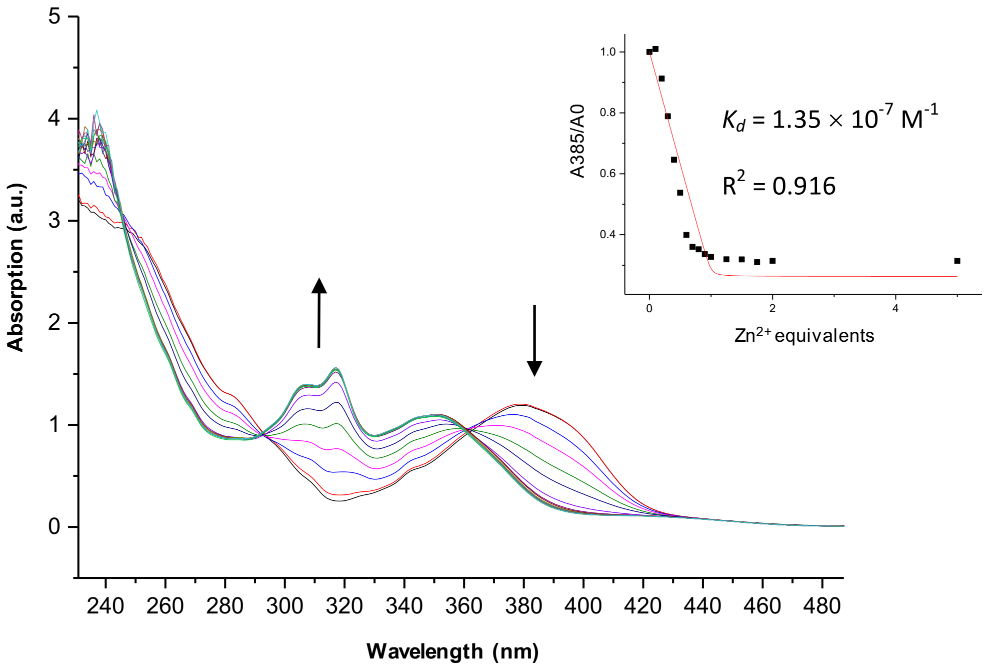

Figure S48 UV-Vis titration of **6** with $Zn(ClO_4)_2.6H_2O$ (0-5 equiv.) in MeCN, C = 100 µM.

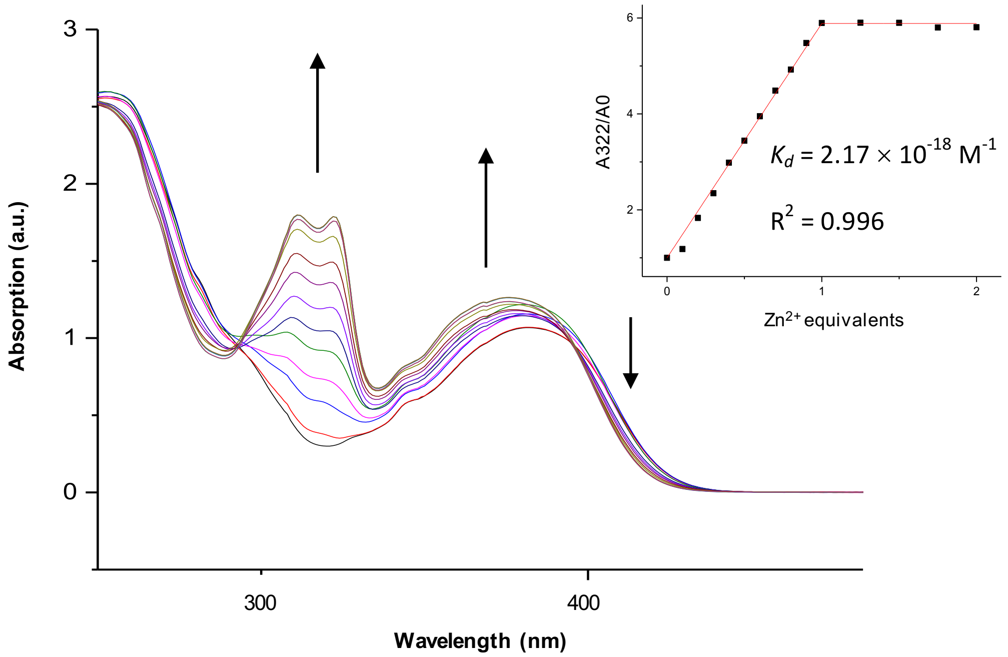

Figure S49 UV-Vis titration of **7** with $Zn(ClO_4)_2.6H_2O$ (0-2 equiv.) in MeCN, C = 100 µM.

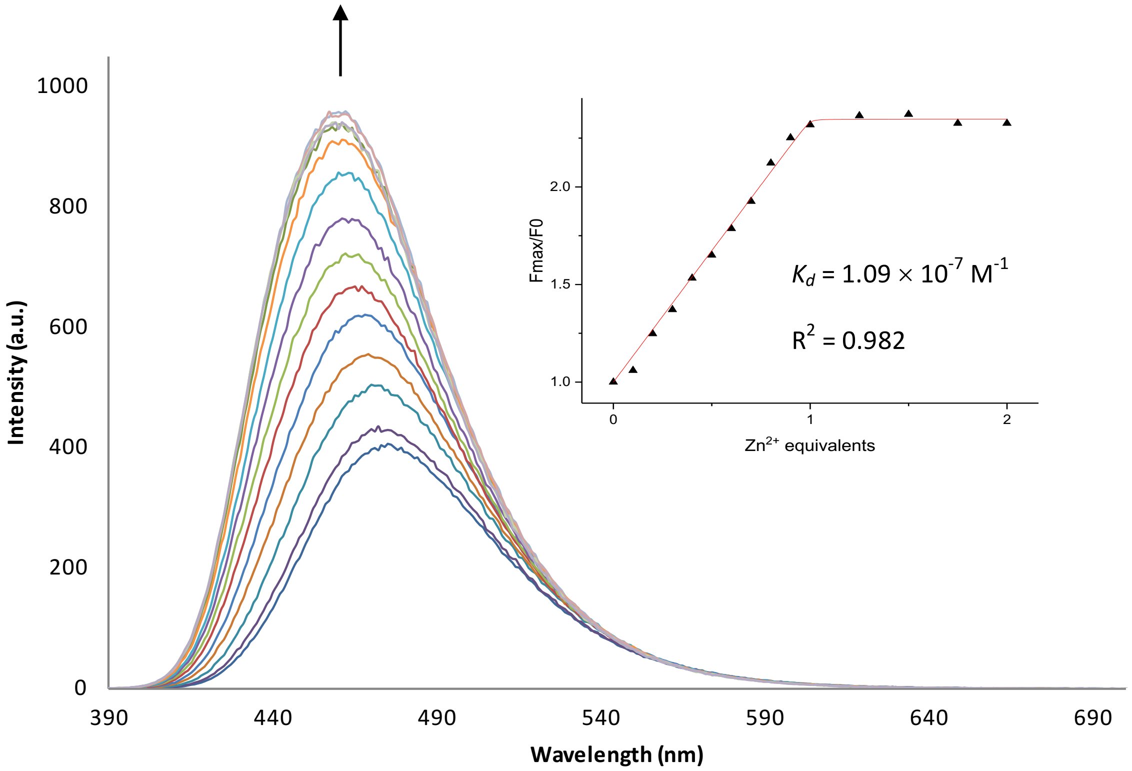

Figure S50 Fluorescence titration of **7** with $Zn(ClO_4)_2.6H_2O$ (0-2 equiv.) in MeCN, C = 100 µM, λ_{ex} = 389 nm.

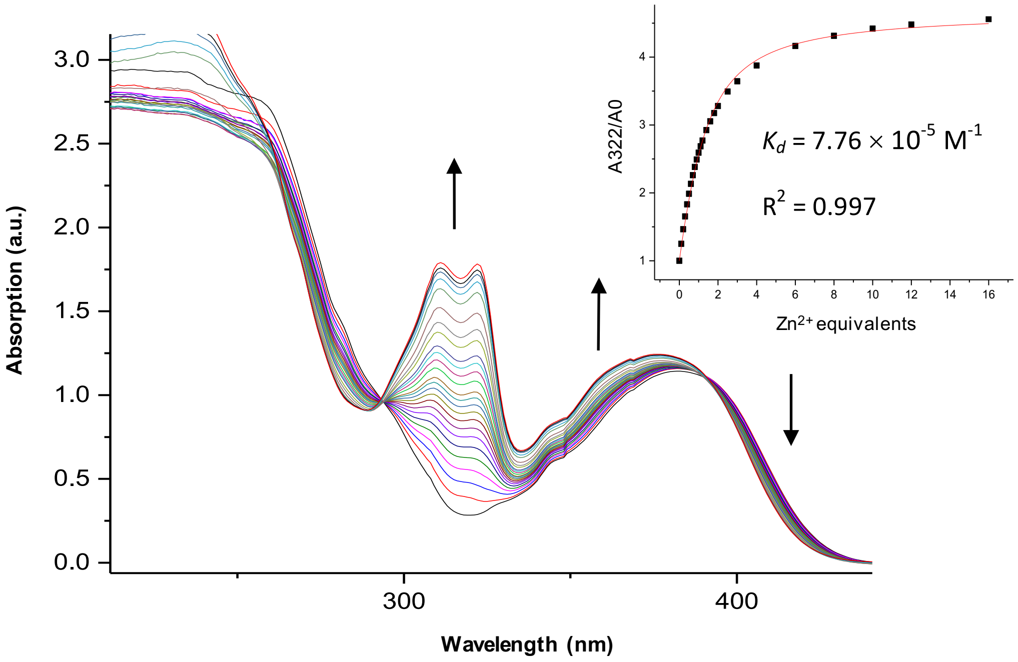

Figure S51 UV-Vis titration of **7** with $Zn(ClO_4)_2.6H_2O$ (0-16 equiv.) in MeCN/H_2O 2%, C = 100 µM.

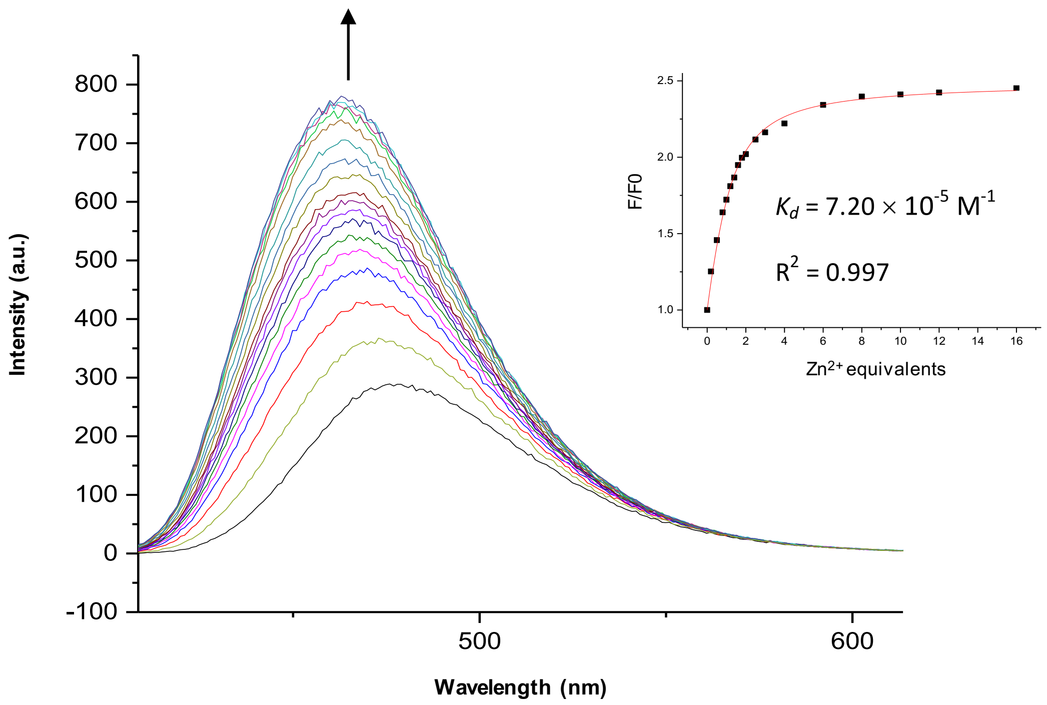

Figure S52 Fluorescence titration of **7** with $Zn(ClO_4)_2.6H_2O$ (0-16 equiv.) in MeCN/H_2O 2%, C = 100 µM, λ_{ex} = 389 nm.

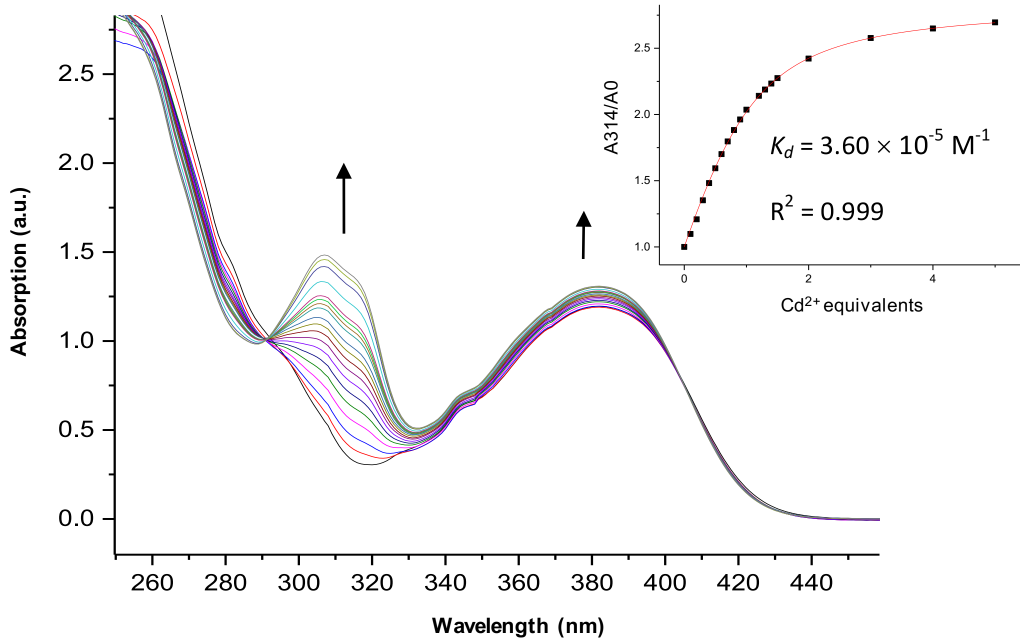

Figure S53 UV-Vis titration of **7** with $Cd(ClO_4)_2.6H_2O$ (0-5 equiv.) in MeCN, C = 100 µM.

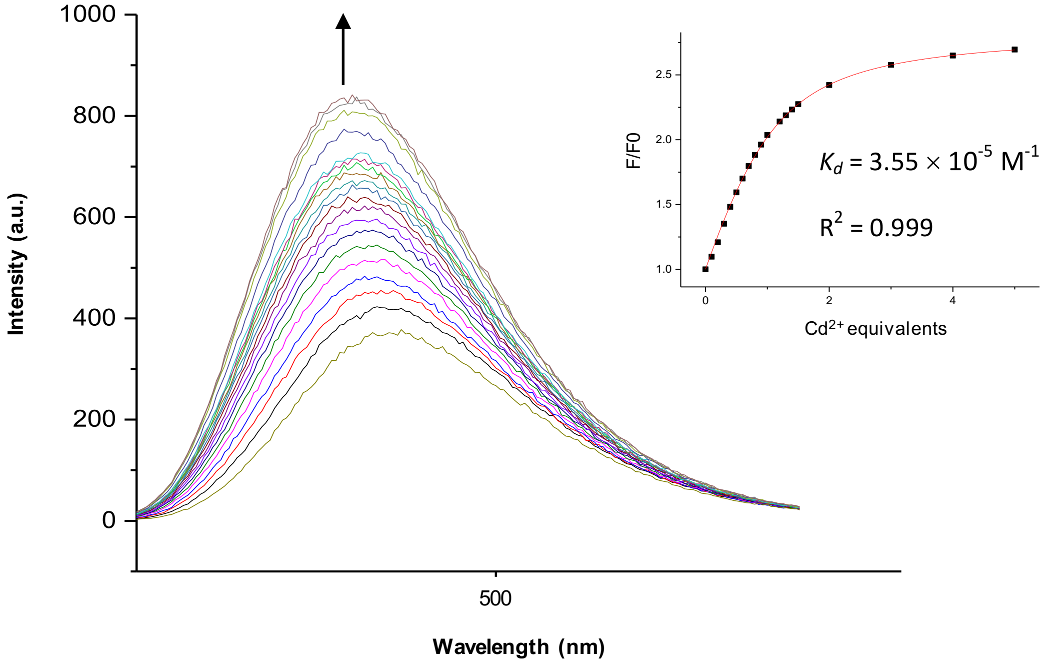

Figure S54 Fluorescence titration of **7** with $Cd(ClO_4)_2.6H_2O$ (0-5 equiv.) in MeCN, C = 100 μM, λ_{ex} = 389 nm.

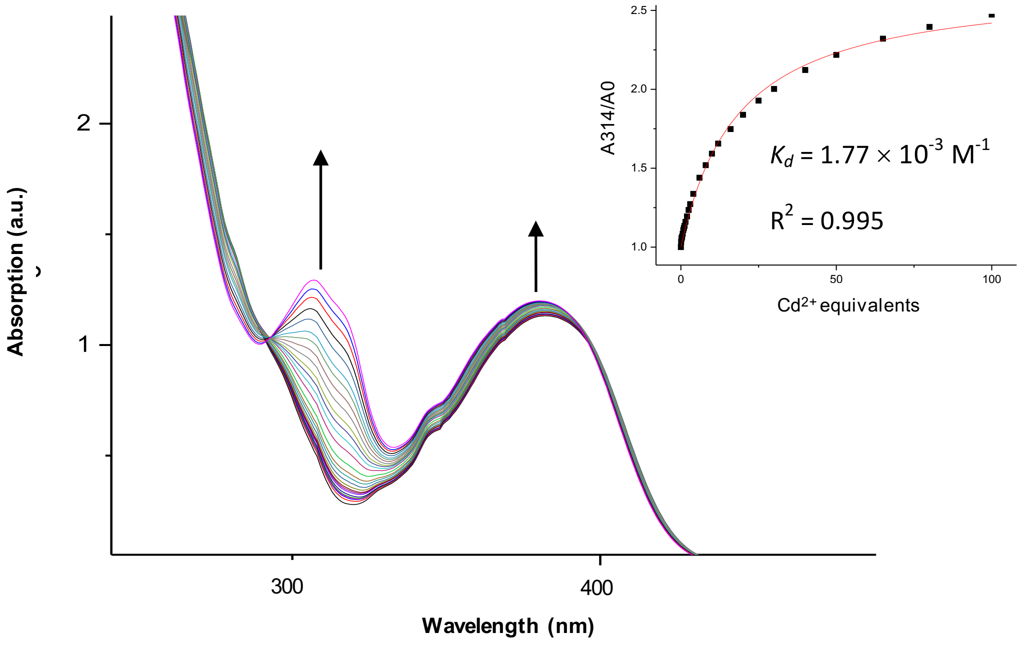

Figure S55 UV-Vis titration of **7** with $Cd(ClO_4)_2.6H_2O$ (0-100 equiv.) in MeCN/H_2O 2%, C = 100 μM.

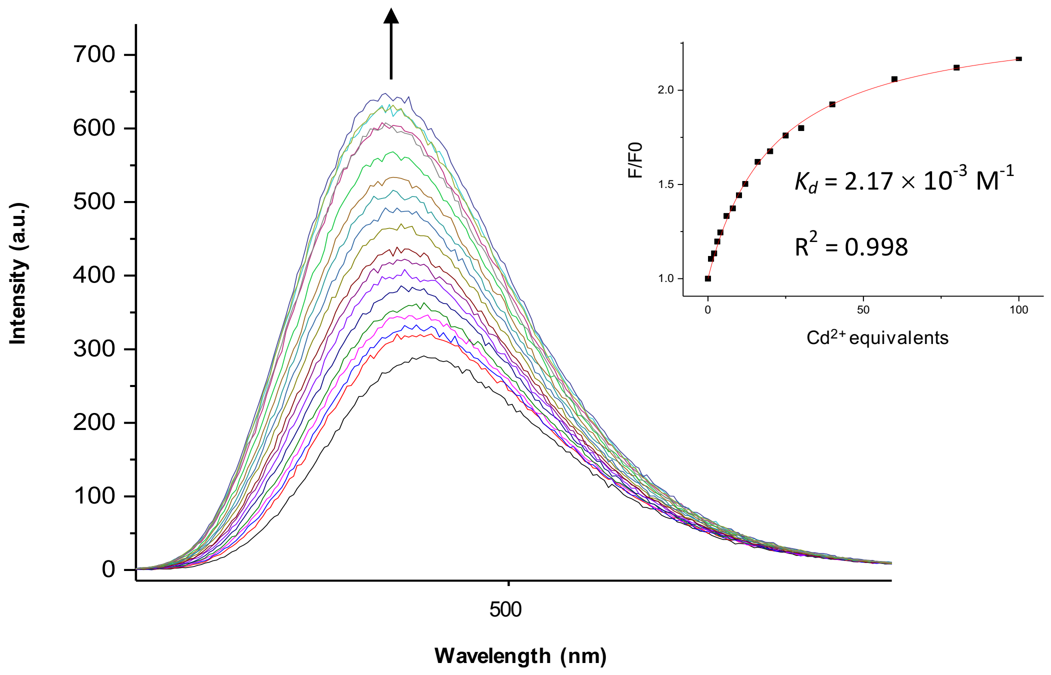

Figure S56 Fluorescence titration of **7** with $Cd(ClO_4)_2.6H_2O$ (0-100 equiv.) in MeCN/H_2O 2%, C = 100 μM, λ_{ex} = 389 nm.

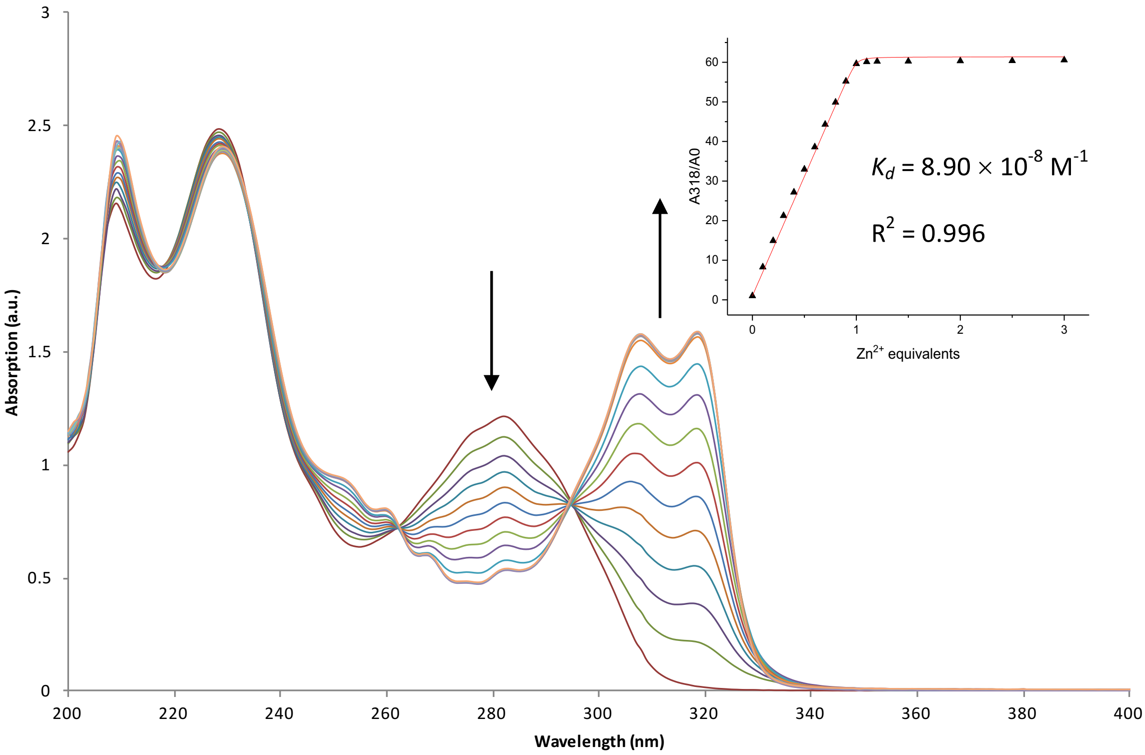

Figure S57 UV-Vis titration of macrocycle **1** with $Zn(ClO_4)_2.6H_2O$ (0-3 equiv.) in MeCN, C = 100 μM.

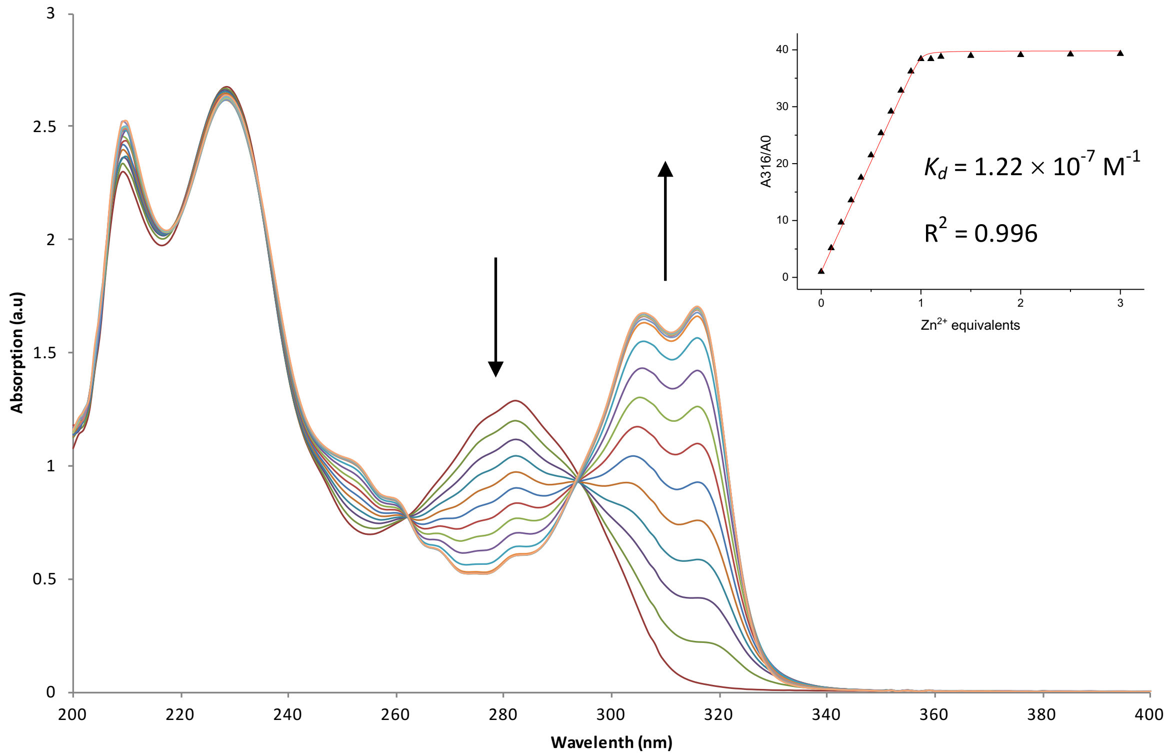

Figure S58 UV-Vis titration of macrocycle **1** with $Zn(ClO_4)_2.6H_2O$ in MeCN/H_2O 2%, C = 100 μM.

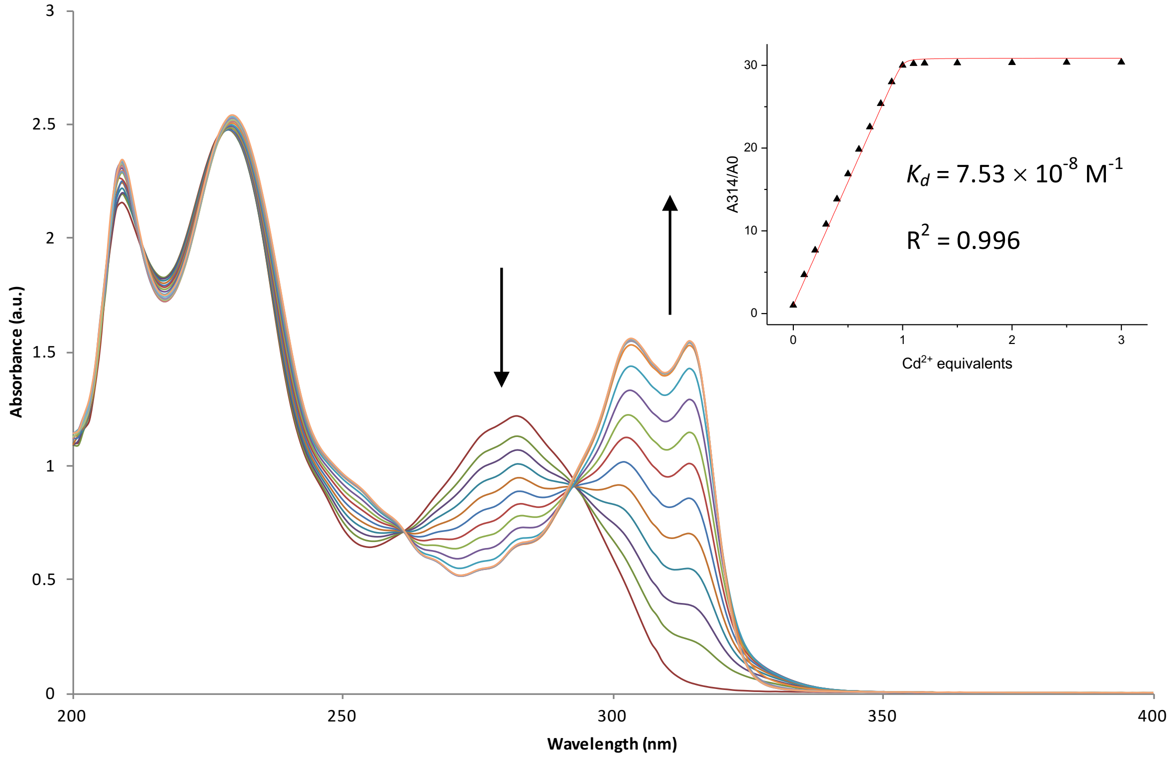

Figure S59 UV-Vis titration of macrocycle **1** with $Cd(ClO_4)_2.6H_2O$ (0-3 equiv.) in MeCN, C = 100 μM.

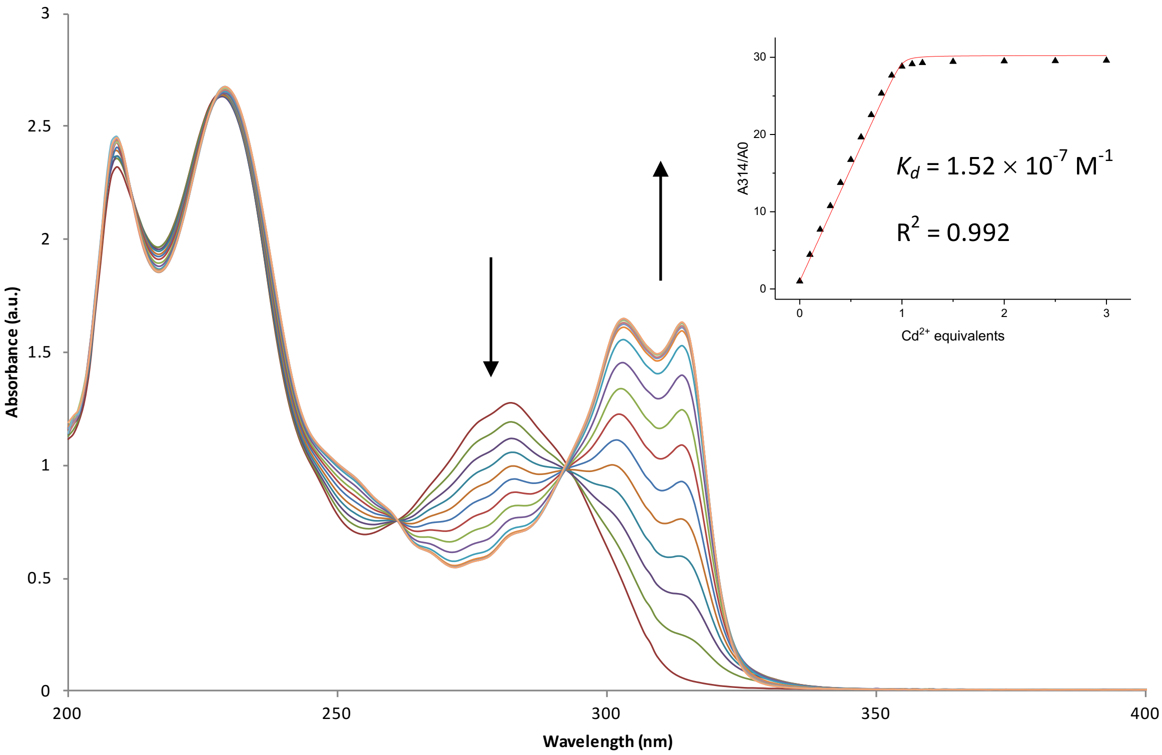

Figure S60 UV-Vis titration of macrocycle **1** with $Cd(ClO_4)_2.6H_2O$ in MeCN/H_2O, C = 100 µM.

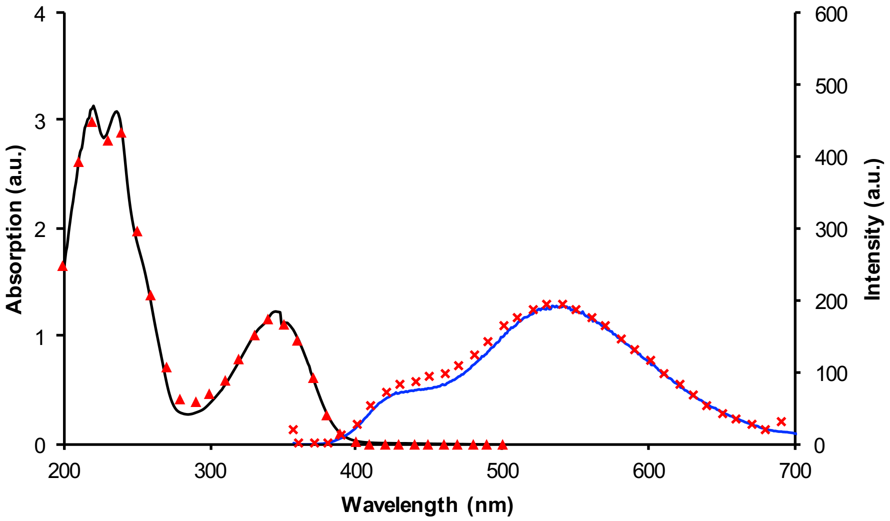

Figure S61 UV-Vis spectra of axle **S3** (black) and in the presence of 5 equiv. of $Zn(ClO_4)_2.6H_2O$ (red triangles). Fluorescence spectra of axle **S3** (blue) and in the presence of 5 equiv. of $Zn(ClO_4)_2.6H_2O$ (red crosses). MeCN, C = 100 µM.

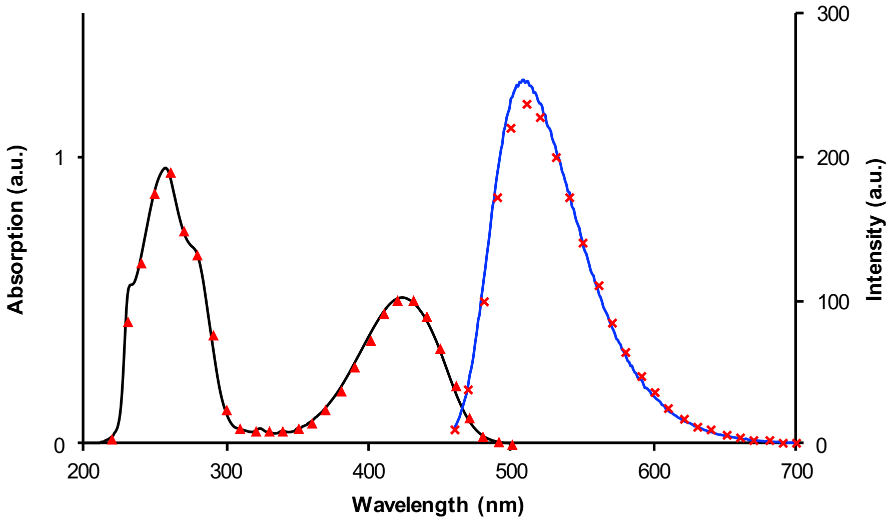

Figure S62 UV-Vis spectra of axle **S6** on its own (black) and in the presence of 5 equiv. of $Zn(ClO_4)_2.6H_2O$ (red triangles). Fluorescence spectra of axle **S6** on its own (blue) and in the presence of 5 equiv. of $Zn(ClO_4)_2.6H_2O$ (red crosses). MeCN, C = 100 μM.

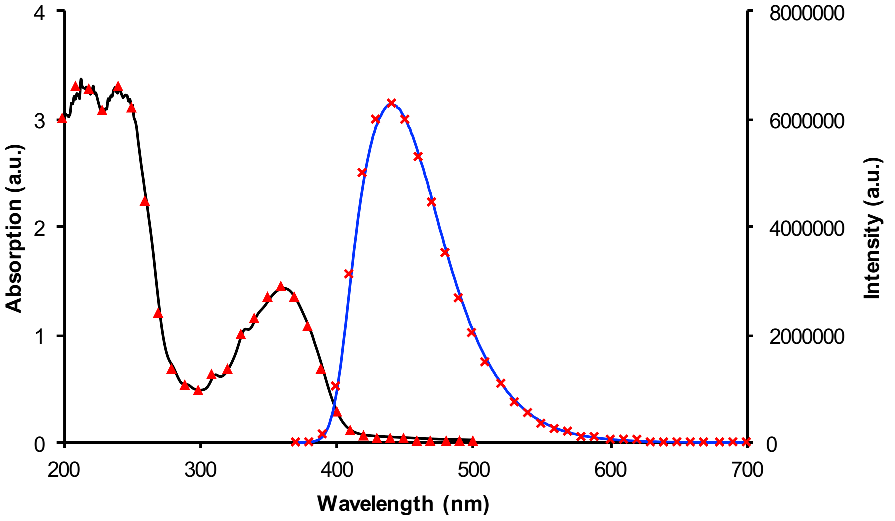

Figure S63 UV-Vis spectra of axle **S11** on its own (black) and in the presence of 5 equiv. of $Zn(ClO_4)_2.6H_2O$ (red triangles). Fluorescence spectra of axle **S11** on its own (blue) and in the presence of 5 equiv. of $Zn(ClO_4)_2.6H_2O$ (red crosses). MeCN, C = 100 μM.

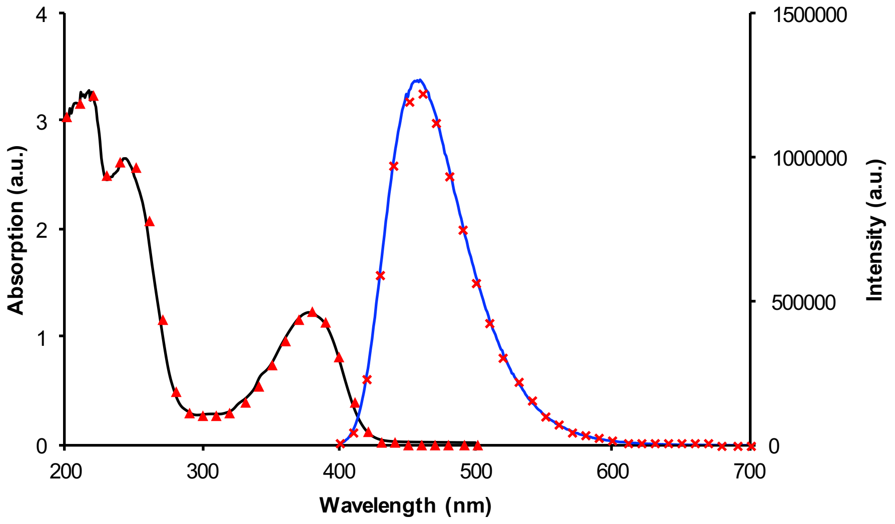

Figure S64 UV-Vis spectra of axle **S14** on its own (black) and in the presence of 5 equiv. of $Zn(ClO_4)_2.6H_2O$ (red triangles). Fluorescence spectra of axle **S14** on its own (blue) and in the presence of 5 equiv. of $Zn(ClO_4)_2.6H_2O$ (red crosses). MeCN, C = 100 µM.

9. X-ray Data

For both **7** and [Zn(**7**)]$^{2+}$, crystals were grown by slow evaporation of MeCN solution. Data were collected at 100 K using a Rigaku 007 HF diffractometer equipped with a Saturn 944+ enhanced sensitivity detector. Cell determination and data collection were done using CrystalClear-SM Expert 3.1; data reduction, cell refinement and absorption correction were performed with CrysalisPro. The structure was solved using SUPERFLIP and refined against F_2 using anisotropic thermal displacement parameters for all non-hydrogen atoms using WINGX and software packages within. Hydrogen atoms were placed in calculated positions and refined using a riding model.

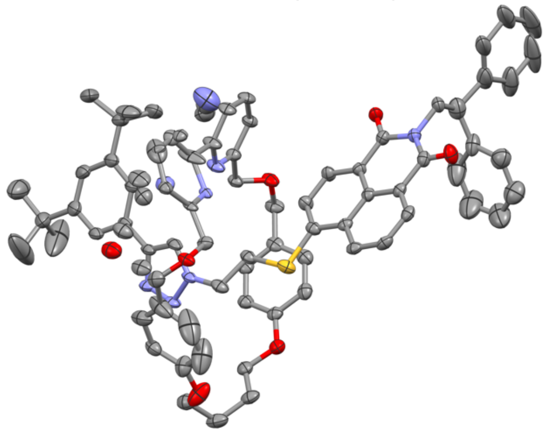

Figure S65 Ellipsoid plot of the asymmetric unit of **7**. Ellipsoids are shown at the 50% probability level. Hydrogen atoms have been omitted for clarity.

Table S2. 2 Crystal data and structure refinement for cu_jp4_srot_2.

Identification code	cu_jp4_srot_2
Empirical formula	$C_{76}H_{77}N_7O_6S$
Formula weight	1216.50
Temperature/K	293(2)

Crystal system	monoclinic
Space group	$P2_1/c$
a/Å	10.4843(2)
b/Å	16.0918(4)
c/Å	40.1750(10)
α/°	90
β/°	90.865(2)
γ/°	90
Volume/Å^3	6777.2(3)
Z	4
ρ_{calc}g/cm^3	1.192
μ/mm^{-1}	0.879
F(000)	2584.0
Crystal size/mm^3	0.300 × 0.100 × 0.090
Radiation	CuKα (λ = 1.54184)
2Θ range for data collection/°	7.038 to 117.868
Index ranges	$-11 \le h \le 11$, $-17 \le k \le 17$, $-44 \le l \le 34$
Reflections collected	38747
Independent reflections	9659 [R_{int} = 0.0482, R_{sigma} = 0.0307]
Data/restraints/parameters	9659/24/890
Goodness-of-fit on F^2	1.062
Final R indexes [I>=2σ (I)]	R_1 = 0.0889, wR_2 = 0.2409
Final R indexes [all data]	R_1 = 0.1030, wR_2 = 0.2547
Largest diff. peak/hole / e Å^{-3}	1.56/-0.58

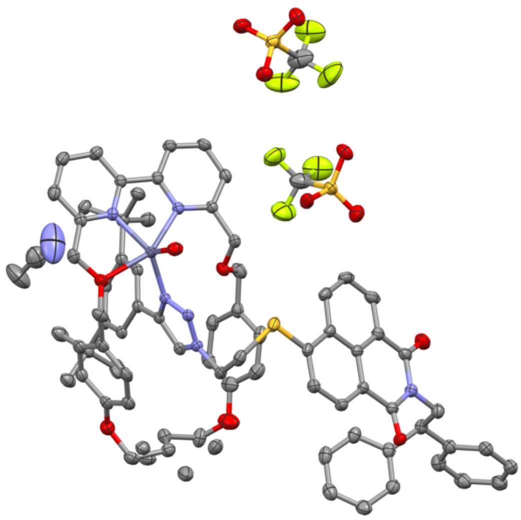

Figure S66 Ellipsoid plot of the asymmetric unit of [Zn(**7**)](OTf)$_2$. Ellipsoids are shown at the 50% probability level. Hydrogen atoms have been omitted for clarity.

Table S2 Crystal data and structure refinement for 2016md_III_095_100K.

Identification code	2016md_III_095_100K
Empirical formula	$C_{78}H_{79}F_6N_7O_{13}S_3Zn$
Formula weight	1598.03
Temperature/K	100(2)

Crystal system	triclinic
Space group	P-1
a/Å	15.1517(4)
b/Å	16.8905(4)
c/Å	17.4286(4)
α/°	111.628(2)
β/°	95.9721(19)
γ/°	111.463(2)
Volume/Å^3	3708.71(17)
Z	2
ρ_{calc}g/cm^3	1.431
μ/mm^{-1}	0.500
F(000)	1664.0
Crystal size/mm^3	0.100 × 0.060 × 0.050
Radiation	MoKα (λ = 0.71073)
2Θ range for data collection/°	3.006 to 64.312
Index ranges	-22 ≤ h ≤ 21, -25 ≤ k ≤ 25, -26 ≤ l ≤ 24
Reflections collected	94990
Independent reflections	24283 [R_{int} = 0.0572, R_{sigma} = 0.0631]
Data/restraints/parameters	24283/0/1017
Goodness-of-fit on F^2	1.015
Final R indexes [I>=2σ (I)]	R_1 = 0.0644, wR_2 = 0.1609
Final R indexes [all data]	R_1 = 0.1113, wR_2 = 0.1898
Largest diff. peak/hole / e Å^{-3}	1.60/-0.94

10. Response of rotaxane S15 to metal ions

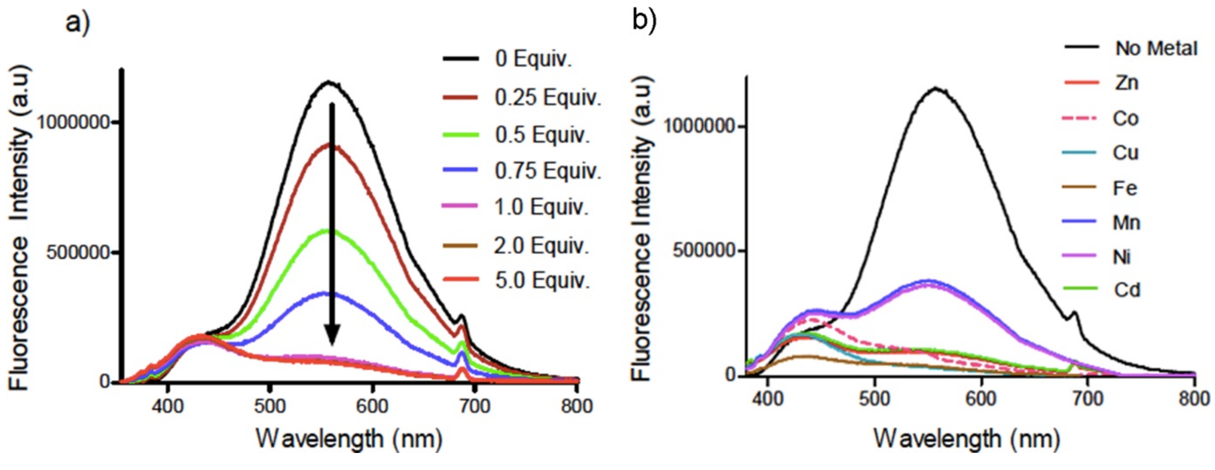

Figure S67 a) Emission profile of rotaxane **S15** (MeCN, 100 μM, λ_{ex} = 343 nm) a) in the presence of varying equivalents of $Zn(ClO_4)_2.6H_2O$, b) in the presence of $M(ClO_4)_2$ (5 equiv.).

11. References

[1] J. E. M. Lewis, R. J. Bordoli, M. Denis, C. J. Fletcher, M. Galli, E. A. Neal, E. M. Rochette, S. M. Goldup, *Chem. Sci.*, **2016**, *7*, 3154–3161.

[2] R. S. Stoll, M. V. Peters, A. Kuhn, S. Heiles, R. Goddard, M. Bühl, C. M. Thiele, S. Hecht, *J. Am. Chem. Soc.*, **2009**, *131*, 357-367.

[3] H. Lahlali, K. Jobe, M. Watkinson, S. M. Goldup, *Angew. Chem. Int. Ed.* **2011**, *50*, 4151–4155.
